# RANBP9 and RANBP10 cooperate in regulating non-small cell lung cancer proliferation

**DOI:** 10.1186/s13046-025-03491-8

**Published:** 2025-08-29

**Authors:** Arturo Orlacchio, Yasuko Kajimura, Lara Rizzotto, Anna Tessari, Shimaa H. A. Soliman, Rosa Visone, Liwen Zhang, Brian Fries, Lino Tessarollo, Joseph Amann, David P. Carbone, Alessia Lodi, Amer Ahmed, Ruggiero Gorgoglione, Giuseppe Fiermonte, Mike Freitas, Dario Palmieri, Jacob Kaufman, Vincenzo Coppola

**Affiliations:** 1 Department of Cancer Biology and Genetics, College of Medicine, Arthur G. James Comprehensive Cancer Center (OSUCCC), The Ohio State Universityand , Columbus, OH USA; 2 NYU Grossman School of Medicine, NYU Langone Health, New York, NY 10016 USA; 3 Division of Hematology, Diabetes, Metabolism and Endocrinology, Yamaguchi University Hospital, Yamaguchi, Japan; 4 Genome Editing Shared Resource, The Ohio State University and OSUCCC, Columbus, OH 43210 USA; 5 Current address: Simpson Querrey Institute for Epigenetics, Department of Biochemistry and Molecular Genetics, Feinberg School of Medicine, Northwestern University, Chicago, IL 60611 USA; 6 Center for Advanced Studies and Technology (CAST), and, Department of Medical, Oral and Biotechnological Sciences, G. d’Annunzio University, Chieti, 66100 Italy; 7 Proteomic Shared Resource, The Ohio State University and OSUCCC, Columbus, OH 43210 USA; 8 Present Address: Neural Development Section, Mouse Cancer Genetics Program, NCI/Center for Cancer Research, NIH, Frederick, MD 21702 USA; 9 Division of Medical Oncology, Ohio State Wexner Medical Center, The Ohio State University and OSUCCC, Columbus, OH 43210 USA; 10 Pelotonia Institute for Immuno-Oncology, OSUCCC, The Ohio State University, Columbus, OH 43210 USA; 11 Department of Nutritional Sciences, College of Natural Sciences, University of Texas at Austin, Austin, TX 78712 USA; 12 Department of Biosciences, Biotechnology and Environment, University of Bari, Bari, 70125 Italy; 13 Department of Medicine and Surgery, LUM University, Casamassima, 70010 Italy

**Keywords:** Lung cancer, Non-small cell lung cancer, NSCLC, CTLH complex, GID complex, RANBP9, RANBPM, SCORPIN, ARMC8, GID4, GID8, TWA1, MAEA, MKLN1, RANBP10, RMND5A, RMND5B, WDR26, YPEL5

## Abstract

**Background:**

RANBP9 and RANBP10, also called Scorpins, are essential components of the C-terminal to LisH (CTLH) complex, an evolutionarily conserved poorly investigated multisubunit E3 ligase. Their role in non-small cell lung cancer (NSCLC) is unknown.

**Methods:**

In this study, first we used stable loss-of function and overexpression inducible cell lines to investigate the ability of either RANBP9 or RANBP10 to form their own functional CTLH complex. Then, we probed lysates from patient tumors and analyzed data from publicly available repositories to investigate the expression of RANBP9 and RANBP10. Finally, we used inducible cell lines in vitro to recapitulate the expression observed in patients and investigate the changes of the proteome and the ubiquitylome associated with either RANBP9 or RANBP10 in NSCLC.

**Results:**

Here, we show that the two Scorpins are both expressed in NSCLC cells and that either of them can independently support the formation of the CTLH complex. Short-term experiments revealed that the RANBP9 and RANBP10 proteins balance each other in terms of expression, and the acute overexpression of one or the other results in significant reshaping of the NSCLC cell proteome and ubiquitylome. A higher RANBP9/RANBP10 ratio is associated with greater proliferation in both NSCLC cell lines and patients. Acute increased expression of RANBP10 slows NSCLC cell proliferation and decreases the level of proliferation-associated proteins, including key players in DNA replication.

**Conclusions:**

We present evidence that the Scorpins act as partial antagonists and work together as one sophisticated rheostat to modulate the CTLH complex ubiquitylation output, which regulates cell proliferation and other key biological processes in NSCLC. These results suggest that the two Scorpins can be considered as targets for the treatment of NSCLC.

**Supplementary Information:**

The online version contains supplementary material available at 10.1186/s13046-025-03491-8.

## Introduction

The scaffold protein containing a C-terminal to LisH (CTLH) domain named RAN Binding Protein 9 (RANBP9; also known as RANBPM) is involved in cell homeostasis, survival, proliferation, adhesion, and migration [1–5]. Its increased expression promotes the signaling of several receptor tyrosine kinases (RTKs), including major players in non-small cell lung cancer (NSCLC) tumorigenesis [6–11].

In NSCLC cells subjected to DNA damage, RANBP9 is not only a target but also an enabler of the ATM (Ataxia Telangiectasia Mutated) protein kinase [12, 13]. RANBP9 protein expression is high in advanced NSCLC, and patients expressing higher levels of RANBP9 protein have worse outcomes when treated with platinum-based regimens, which is in line with the hypothesis that RANBP9 is protective of cancer cells when subjected to DNA damage [14]. Hence, targeting RANBP9 could be a valid strategy to treat NSCLC in combination with other therapeutic modalities [15].

RANBP9 operates in the context of the ubiquitously expressed unconventional multisubunit E3 ligase CTLH complex, where it constitutes the inner core and tightly binds to GID8 (Glucose-Induced degradation Deficient 8) [3, 16–18]. This multisubunit enzyme is emerging as a central regulator of mammalian cell metabolism and regulates key bioenergetic signaling nodes, such as AMP-activated protein kinase (AMPK) and mammalian target of rapamycin (mTOR) [19–22]. Currently, there are 11 known members of the CTLH complex. In addition to RANBP9 and GID8, other established core members are ARMC8 (Armadillo Repeat Containing protein 8), MAEA (Macrophage Erythroblast Attacher E3 ligase), RMND5A and RMND5B (Required in Meiotic Division 5 A and B). On the other hand, GID4 (Glucose-Induced degradation Deficient 4), WDR26 (WD Repeat containing protein 26), and YPEL5 (Yippee Like protein 5) are peripheral members [2, 23]. Recent biochemical and structural studies have shown that MAEA and RMND5 bind to a RANBP9-GID8-ARMC8 core and, together, provide E3 enzymatic activity [24]. Finally, MKLN1 (Muskelin 1) has been shown to be not only a peripheral member but also a substrate of the complex, and its levels can be used as a proxy for CTLH enzymatic activity [17, 20].

Although the CTLH complex was initially characterized as a heterodecameric aggregate, it is becoming increasingly clear that its proteins can assemble in different configurations that include or exclude selected core and peripheral members. For example, RMND5A and RMND5B are mutually exclusive [3]. In addition, the two splicing isoforms of ARMC8, ARMC8α and ARMC8β, can determine the inclusion (ARMC8α) or exclusion (ARMC8β) of GID4, the first and only known substrate receptor until recently [19, 25]. The presence of RANBP9, GID8, RMND5A, and WDR26 together has been shown to promote proliferation [3, 25].

The topological features of the CTLH E3 ligase have been inferred from studies in *S. cerevisiae,* where its counterpart, the GID (Glucose-Induced degradation Deficient) complex, responds to metabolic stress, modulates mitochondrial functions and participates in the disposal of enzymes necessary for gluconeogenesis [5, 26–28]. Elegant structural work has shown that the GID complex can assemble into “supra-molecular” oval-shaped rings with a diameter equivalent to the length of the proteasome and quickly ubiquitylate multimeric metabolic enzymes in the central cavity [29]. Yeast Gid1 is essential for the assembly of the complex and tightly binds Gid8. Together, Gid1 and Gid8 provide the inner core on which the structure is built, and their proteins stabilize each other [30]. Deletion of Gid1 disrupts the formation of the complex [26, 30]. Therefore, it has been hypothesized that ablation of RANBP9 disrupts the formation of the whole CTLH complex [31]. However, the eleventh and understudied member of the CTLH complex is a highly similar RANBP9 paralog called RANBP10, which presents the same domains that are essential for aggregation into the CTLH complex [3, 13, 32–34]. RANBP9 and RANBP10 are also called Scorpins (Spry-containing Ran-binding proteins) to distinguish them from other RAN-binding proteins that are involved mainly in nuclear‒cytoplasmic shuttling [34]. RANBP9 is markedly expressed in highly proliferative precursors, whereas RANBP10 expression becomes predominant in more differentiated cells during erythroid maturation. Moreover, both RANBP9 and RANBP10 can form their own CTLH complex [35]. However, the reciprocal dynamics governing the expression of Scorpins in the same cell type are not known. Although RANBP10 is ubiquitously coexpressed with RANBP9 and consistently coimmunoprecipitates with other CTLH proteins both in humans and in mice, the function of RANBP10 is not well understood in general [3, 23, 32, 36]. Apart from a potential tumor-promoting role in glioblastoma [37], the relevance of RANBP10 in cancer has not been addressed.

Taking into consideration evidence from different model systems, we reasoned that RANBP9 and RANBP10 need to be studied together to fully understand their biological roles because they are both the result of duplication of the ancestral yeast Gid1 gene [13, 23]. Phenotypic and structural similarities, as well as differences, indicate that these genes might have only partially overlapping functions and are likely to cross-regulate each other [13].

Here, we present data that show how the two Scorpins work in concert and how their modulatory role on the CTLH complex ubiquitylation output depends on the ratio of their amount. The predominant expression of RANBP9 over RANBP10 in NSCLC correlates with increased proliferation in both NSCLC cell lines and patients, where increased expression of RANBP10 resulted in a reduction in cell proliferation and in the level of proteins involved in cell proliferation. In vitro, RANBP9 expression increased glycolysis.

Here, we reveal that the two Scorpins constitute a unique sophisticated rheostat that modulates the expression and ubiquitylation of a variety of proteins that participate in fundamental NSCLC oncogenic processes, such as cell proliferation and a coordinated adjustment of metabolism.

## Materials and methods

### Generation of cell lines and KOs

A549 and H460 NSCLC cell lines (see supplementary info) were purchased from ATCC and cultured in RPMI 1640 medium (Gibco™) supplemented with 10% FBS (Gibco™) and MycoZap™ Plus-CL (Lonza).

sgRNAs were designed at http://crispr.mit.edu (see supplementary info). Briefly, for each guide, two DNA primers were designed and then annealed and phosphorylated using T4 polynucleotide kinase (NEB). Phosphorylated sgRNAs were then cloned and inserted into the vectors pSpCas9(BB)−2A-GFP (5’ prime sgRNA) and pSpCas9(BB)−2A-Puro (3’ prime sgRNA) via the BbsI restriction enzyme (NEB) and the Rapid DNA Dephos & Ligation Kit (ROCHE). Following the transformation of the ligase reaction plasmid DNA into DH5α competent cells (Thermo Fisher Scientific), the plasmid DNA was purified via endotoxin-free Maxiprep kits (QIAGEN). NSCLC cell lines (~ 1 × 10^6^) were transfected with both sgRNA-containing vectors via Lipofectamine™ 3000 Transfection Reagent (Thermo Fisher Scientific) according to the manufacturer’s specifications, and after 48 h, single cells were sorted for GFP expression via a BD FACSAria™ II Cell Sorter. Scorpin WT or Scorpin DKO A549 cells were transduced via a 4D-Nucleofector X Unit (Lonza) according to the manufacturer’s optimized protocol. Briefly, 2 × 10^5 cells were resuspended in 20 µl of SF buffer and electroporated via the nucleofection program CM-130 with 0.1 μg of AAVS1-TRE3G-EGFP, AAVS1-TRE3G-RANBP9/AAVS1-TRE3G-RANBP9-2xV5 or AAVS1-TRE3G-RANBP10 AAVS1-TRE3G-RANBP10-2xHA in the presence of 0.1 μg of pX330-U6-Chimeric_BB-CBh-hSpCas9-hGem (1/110)-AAVS1 gRNA. The latter allows CRISPR/Cas9-mediated integration of inducible plasmids into the AAVS1 safe harbor site of the human genome. Forty-eight hours post-transfection, puromycin (1 µg/mL) was applied to enrich the cells with successful transgene integration. The AAVS1-TRE3G and pX330-U6-Chimeric_BB-CBh-hSpCas9-hGem (1/110) base plasmids for safe-harbor integration of the inducible system were acquired from Addgene (see also Extended Material) [38, 39].

### Preparation of MEF cell cultures

All the animal studies were conducted in accordance with a protocol approved by the Institutional Animal Care and Use Committee (IACUC) of Ohio State University. Embryos from RanPBP9, RanBP10 KO and Double KO mice (see also Extended Material) were isolated at E12.5. After the heads, tails, limbs, and most of the internal organs were removed, the embryo carcasses were minced, passed through a 70 µm cell strainer, and then seeded into 10 cm cell culture dishes in 10 mL of high-glucose DMEM supplemented with 15% FBS, 1% penicillin‒streptomycin (Gibco™) and 0.01% 2-mercaptoethanol (Sigma-Aldrich). Primary MEFs were cultured in 3% oxygen and passaged two or three times to obtain a morphologically homogenous culture. Immortalized MEFs were generated by transfecting early-passage primary cells with 2 µg of Large-T antigen-expressing plasmid via Lipofectamine™ 3000. The cells were then cultured in normal oxygen (20%) and passaged for an additional 5 generations to obtain a stable population.

### Western blot

The cells and tumor tissues were homogenized on ice in NP-40 buffer supplemented with Halt protease and phosphatase inhibitor cocktail (Thermo Fisher Scientific). The protein concentration was determined via the use of the Bio-Rad protein assay dye (Bio-Rad). Western blot analysis was performed using 30 to 50 μg of protein run on Mini-PROTEAN TGX precast gels (Bio-Rad).

Primary antibodies (see also Extended Material) were used at a dilution of 1:1,000 in 5% milk in TBS-T. Signals were detected with HRP-conjugated secondary antibodies and the chemiluminescence substrate SuperSignal West Pico PLUS or Femto (Thermo Fisher Scientific). Equivalent loading among samples was confirmed with an anti-vinculin antibody (Cell Signaling). Images were acquired via the KwikQuant Digital Western Blot Detection System (Kindle Biosciences, LLC). Protein expression levels were quantified via optical densitometry via ImageJ software version 1.53t (https://imagej.nih.gov/ij/) and KwikQuant Image Analyzer 5.4 (https://kindlebio.com/12-downloads).

### RNA extraction and real-time qPCR

The cells and tumor tissues were homogenized on ice in TRIzol™ reagent (Invitrogen™), and RNA was extracted according to the manufacturer’s guidelines. Following extraction, 1 µg of total RNA was then treated with DNase and converted into cDNA via the Maxima First Strand cDNA Synthesis Kit for RT‒qPCR with dsDNase (Thermo Fisher Scientific).

For real-time qPCR, 10–15 ng of cDNA was amplified via TaqMan™ Fast Advanced Master Mix. Samples were amplified simultaneously in triplicate in one assay run via TaqMan probes specific for (RANBP9, RANBP10, GID8, WDR26, ARMC8, RMND5A, RMND5B, YPEL5, MAEA and MKLN1). OAZ1 and GAPDH were used as endogenous controls for human tumor samples and cell lines, respectively. Analysis was performed with GraphPad Prism 10.0 software via the Δ-ct method (see also Extended Material).

### Proteomics

#### Cell lysis

The cell pellets were washed with PBS three times before being resuspended in 5% SDS buffer in 50 mM TEAB (triethylammonium bicarbonate) solution. The samples were then vortexed briefly before sonication by using a Bioruptor® Pico (Diagenode, Denville, NJ) following the manufacturer’s suggested protocol. Briefly, the sonication temperature was set at 4 °C; the sonication cycle was set at 30 s on and 30 s off. A total of 20 cycles were performed for the cell pellets. After sonication, the samples were centrifuged at 20,000 × g for 15 min at 4 °C to remove any remaining insoluble material. The protein concentrations were measured via a Qubit fluorometer (Thermo Fisher Scientific).

#### S-trap digestion

Proteins were digested with trypsin via S traps (Protifi, Fairport, NY). For the TMT-labeled samples, 200 µg of each protein sample was subjected to trypsin digestion on an S-trap microcolumn (K02-micro); for the ubiquitylation enrichment samples, 2 mg of each protein sample was digested on an S-trap midi column (C02-Midi). Briefly, samples were reduced with DTT and alkylated with iodoacetamide before the addition of 12% phosphoric acid (to a final volume of 1.2%). The proteins were then diluted with S-Trap binding buffer (MeOH:TEAB, 90:10 v/v) at a 1:6 (sample:S-Trap binding buffer, v/v) ratio. The sample was then applied to the S-Trap column and washed three times with S-Trap binding buffer 4 times. Sequencing grade trypsin (Promega) dissolved in 50 mM TEAB was added to the sample at a 1:50 (trypsin:protein, w:w) ratio for TMT-labeled samples or a 1:200 ratio for ubiquitylated enrichment samples. The sample was incubated overnight at 37 °C and eluted sequentially with 50 mM TEAB, 0.2% formic acid, and 0.2% formic acid in 50% acetonitrile. The samples were pooled and dried in a centrifuge concentrator for further use.

#### TMT labeling

One hundred micrograms of peptides in 50 mM TEAB solution were labeled per the manufacturer’s instructions (A44522, Thermo Fisher Scientific). The TMT-labeled peptides were then pooled together and fractionated via a Pierce high-pH reversed-phase peptide fractionation kit (Cat# 84,868, Thermo Fisher Scientific). Peptides eluted with 5, 10, 12.5, 15, 17.5, 20, 22.5, 25 and 50% acetonitrile/triethylamine (0.1%) solutions were collected and analyzed on a Fusion Orbitrap mass spectrometer.

#### Ubiquitylation enrichment

The dried peptides were resuspended in PTMScan HS IAP Bind Buffer and placed on ice. PTMScan HS Magnetic Immunoaffinity Beads were washed four times with ice-cold 1X PBS. The dissolved peptides were combined with the washed magnetic beads and incubated on a tabletop mixer at 4 °C for six hours. After incubation, magnetic beads with bound peptides were placed on a magnetic separator, and unmodified peptides were removed. The magnetic beads were washed four times with chilled HS IAP Wash Buffer, followed by two washes with chilled LC–MS Grade H_2_O. Ubiquitylated peptides were eluted twice by the addition of 0.15% TFA with gentle agitation for 10 min. Ubiquitylated peptides were placed into glass vials and dried prior to LC–MS analysis.

#### Nano-LC/MS/MS analysis

Nanoliquid chromatography-nanospray tandem mass spectrometry (nano-LC‒MS/MS) for protein identification was performed on a Thermo Scientific orbitrap Fusion mass spectrometer equipped with an EASY-Spray™ Sources operated in positive ion mode. The samples were separated on an easy spray nanocolumn (Pepmap™ RSLC, C18 3 µ 100 A, 75 µm X250 mm Thermo Scientific) via a 2D RSLC HPLC system from Thermo Scientific. Mobile phase A was 0.1% formic acid in water, and acetonitrile (with 0.1% formic acid) was used as mobile phase B. The flow rate was set at 300 nL/min. For the TMT samples, a 3-h gradient was used after the samples were desalted via a trap column. For ubiquitylated peptides, samples were directly loaded and separated on an easy spray nanocolumn bypassing the desalting column, and a 1-h gradient was used for analysis.

MS/MS data were acquired with a spray voltage of 1.95 kV, and a capillary temperature of 305 °C was used. The scan sequence of the mass spectrometer was based on the preview mode data-dependent TopSpeed™ method. To achieve high mass accuracy MS determination, the full scan was performed in FT mode, and the resolution was set at 120,000 with internal mass calibration. For TMT-labeled samples, FT mode (resolution set at 50,000) was used for MS2 data acquisition to ensure that mass tags that differ by only one N15 and C13 can be well resolved for accurate quantitation. For ubiquitinated samples, MSn was performed via HCD in ion trap (IT) mode to ensure the highest signal intensity of the MSn spectra. Three FAIMS compensation voltages (cv = −50, −65 and −80v) were used for data acquisition. The AGC target ion number for the FT full scan was set at 4 × 10^5^ ions, the maximum ion injection time was set at 50 ms, and the microscan number was set at 1. The HCD collision energy was set at 32%. The AGC target ion number for the ion trap MSn scan was set at 3.0E4 ions, the maximum ion injection time was set at 35 ms, and the microscan number was set at 1. Dynamic exclusion is enabled with a repeat count of 1 within 60 s and a low mass width and high mass width of 10 ppm. Data analysis and quantitation were performed via MASCOT on Proteome Discoverer following workflows recommended by Thermo.

### Lactate production measurement

#### Tracer treatment, metabolites extraction

A549 i-Scr (control), A549 i-BP9-2xV5, and A549 i-BP10-2xHA were seeded at 2 × 10^5^ cells/well and induced on the next day with 10 ng/ml doxycycline (Sigma-Aldrich, Cat# D9891). After 24 h Cells were provided with tracer medium DMEM/F-12 (USBiologica Cat# D9807-10) lacking glucose and pyruvate, supplemented with a homemade mixture of all amino acids at concentrations equivalent to those in commercial DMEM/F-12, along with 5 mM [U-13C]-glucose and 2 mM glutamine for 6 h. Then metabolites were extracted as previously described [40]. Briefly, the medium was removed, cells were placed on ice, and washed with ice-cold 0.9% NaCl. Cells were flashed with 500 µl of methanol, scraped and collected into Eppendorf. 500 µl of chloroform and 400 µl of milli-Q water was added. The mixture was then vortexed for 30 s and centrifuged at 14,000 r.p.m. for 15 min at 4 °C. The upper phase was recovered, dried using a SpeedVac and stored at −80 °C until derivatization. At the beginning and end of the extraction, norvaline and norleucine were added as internal standards to evaluate the efficiency of the extraction and derivatization, respectively.

#### Metabolite derivatization

Dried sample were dissolved in 50 µl of methoxamine reagent (Sigma-Aldrich, Cat# M6524) and sonicated for 10 min. Samples were then kept at 40 °C for 90 min, then at 70 °C for 30 min after the addition of 70 μl of MTBSTFA + 1% TBDMCS (Thermo Fisher Scientific, Cat# TS-48920) [41].

#### GS-MS measurement and analysis

A Waters Micromass Quattro Micro GC triple quadrupole mass spectrometer, directly coupled to an Agilent 6890 N gas chromatograph, was employed to analyze the samples. Samples (1 µl) were injected in split mode (5:1 ratio) into the gas chromatograph and separated using a fused silica DB-5MS + DG capillary column (30 m, 0.25 mm inner diameter, 0.25 µm stationary phase thickness). The injector temperature was set to 240 °C. High purity helium was used as the carrier gas at a constant flow rate of 1 mL min − 1. The column temperature was initially kept at 70 °C for 2 min, ramped up first to 140 °C at 3 °C min − 1, then to 150 °C at 1 °C min − 1, and finally to 280 °C at 3 °C min − 1. The interface and ion source temperatures were 280 and 240 °C, respectively.

Data were acquired in full scan mode and processed using MassLynx software (v. 4.0). The integrated peak areas for m/z 261, 262, 263, and 264, corresponding to the lactate isotopologues m0, m1, m2, and m3, respectively, were extracted [41]. The observed MIDs were corrected for natural abundance of stable isotopes by FluxFix to generate corrected MIDs [42].

#### Statistical analysis

GraphPad prism 5 Software was used to conduct the statistical analysis. Data are presented as the mean ± SD of 5 independent experiments and are assessed with unpaired, two-tailed Student’s *t*-test. GC–MS analysis was carried out in a blinded manner. *P* < 0.05 was considered statistically significant and *P* values are indicated in the figure.

## Results

### The combined deletion of Scorpins causes the disappearance of GID8 and the functional inactivation of the CTLH complex

**Fig. 1:**
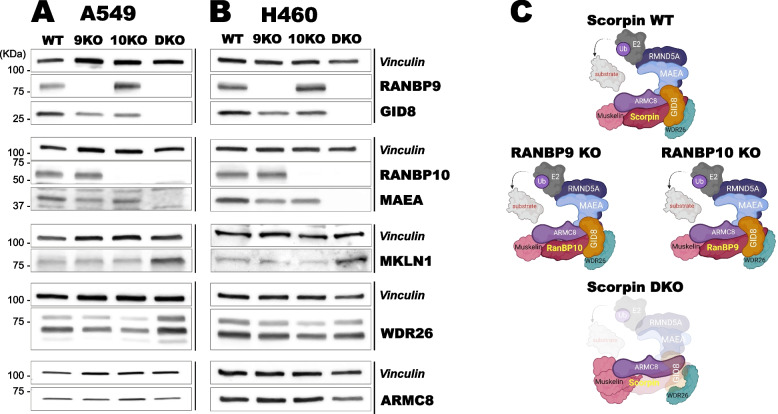
The combined deletion of Scorpins disrupts the CTLH complex in NSCLC cells. **A**, **B** CRISPR/Cas9 was used to generate A549 (**A**) and H460 (**B**) cell lines lacking RANBP9 (9KO), RANBP10 (10KO), or both (DKO). Total cell lysates were analyzed by WB for the presence of the indicated CTLH proteins. The vertical lines on the right side of the panels represent blots from the same gel. Vinculin was used as a loading control for each blot. **C** Illustrations of CTLH complex formation and disruption in Scorpin WT, 9KO, 10KO, and DKO NSCLC cells generated via Biorender (www.biorender.com)

We previously generated RANBP9 shRNA-knockdown and complete knockout (KO) NSCLC cells [12, 43, 44]. However, we did not assess the levels of RANBP10, which is reported to be expressed at lower levels than its paralog. For example, in HEK293T cells, the number of RANBP10 protein copies (7.6 × 10^4^) is estimated to be approximately one-third that of RANBP9 (2.3 × 10^5^) [45]. To better characterize the role of both Scorpins in NSCLC in the context of the CTLH complex, we used CRISPR/Cas9 technology to generate RANBP10 knockout (KO) A549 and H460 cell lines. We also generated NSCLC cells in which both Scorpins were genetically inactivated (double-KO [DKO] cells) (Fig. 1A-1B). When tested for other CTLH members, DKO cells presented nearly complete ablation of GID8 and MAEA, whereas the levels of ARMC8 and WDR26 did not appear to consistently change among the two cell lines and different genotypes. On the other hand, the levels of MKLN1 were consistently elevated in DKO cells (Fig. 1A-1B). The combined Scorpin deletion had similar effects on the levels of GID8 and MAEA in mouse embryonic fibroblasts (MEFs) (Supplementary Fig. 1 A). These cells were derived from embryonic stem cell-generated mice deficient in RANBP9, RANBP10, or both Scorpins together (DKO), indicating that the disappearance of GID8 and MAEA is not dependent on cell type or species and is not caused by the use of CRISPR/Cas9 [46, 47]. MKLN1 levels were increased in DKO MEFs, which was consistent with observations in NSCLC cells, whereas WDR26 levels appeared to be unchanged in Scorpin-mutant MEFs (Supplementary Fig. 1 A).

RT‒PCR evaluation of GID8 and MAEA mRNA levels in both A549 and H460 DKO cells generally revealed a modest increase (less than 25%). The exception was GID8 mRNA, whose expression modestly but significantly decreased in H460 cells. These results indicated that changes in GID8 and MAEA protein levels were not due to changes in the corresponding transcripts (Supplementary Fig. 1B-C), which is in agreement with previous observations in cell lines [17, 35, 48].

Overall, both RANBP9 and RANBP10 can independently stabilize GID8 in mammalian cells, and only their stable combined deletion disrupts the formation of a functional CTLH complex via protein-mediated mechanisms.

### RANBP9 or RANBP10 acute re-expression is sufficient to stabilize GID8 and MAEA and restore CTLH complex formation

**Fig. 2:**
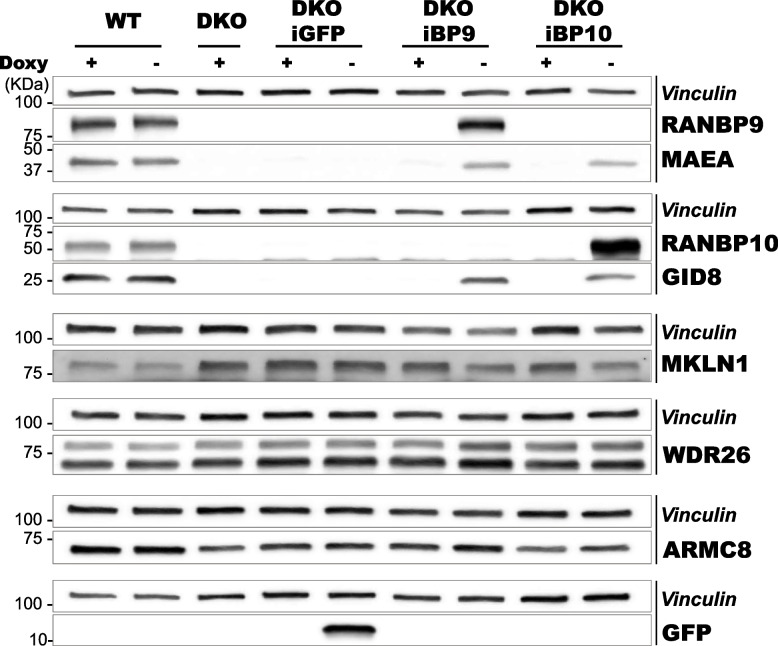
RANBP9 or RANBP10 re-expression is sufficient to stabilize GID8 and MAEA and restore CTLH complex formation. Scorpin DKO A549 cells bearing doxycycline (Doxy)-inducible enhanced GFP (A549 iGFP), RANBP9 (DKO iBP9), or RANBP10 (iBP10) cDNA, together with Scorpin WT A549 cells, were left untreated or exposed to Doxy at 1 mg/mL for 24 h before harvesting. Total cell lysates were probed by WB for the presence of the indicated CTLH proteins. The vertical lines on the right side of the panels group together blots from the same gel. Vinculin was used as a loading control for each blot

To further prove that both RANBP9 and RANBP10 can independently stabilize GID8 and enable the formation of a canonical CTLH complex, we generated A549 DKO cells in which either RANBP9 alone or RANBP10 alone can be re-expressed by a doxycycline (Doxy)-inducible system (Supplementary Fig. 2 A). The re-expression of RANBP9 or RANBP10 resulted in the clear reappearance of GID8 and MAEA, whereas the amount of MKLN1, which is also a substrate of the complex, decreased to levels similar to those of the WT (Fig. 2, Supplementary Fig. 2B) [17, 20]. The de novo expression of GFP had no appreciable effects on the Scorpins or other CTLH proteins. We concluded that in NSCLC cells, the expression of either one of the two Scorpins is sufficient to stabilize its assembly and restore the formation and function of the CTLH complex.

### RANBP9 and RANBP10 cross-regulate each other’s expression

**Fig. 3:**
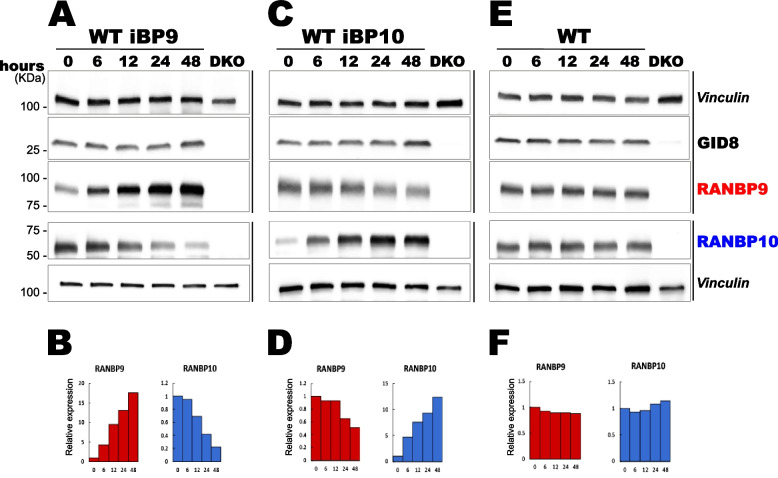
RANBP9 and RANBP10 cross-regulate each other’s expression. *Time course of inducible RANBP9 and RANBP10 expression in Scorpin WT A549 iBP9 and iBP10 cells*. Total cell lysates from Scorpin WT A549 (**E**), iBP9 (**A**), and iBP10 (**C**) cells were probed by WB for the expression of RANBP9 (red), RANBP10 (blue), or GID8 before and after exposure to Doxy at 1 mg/mL for the indicated times. A549 Scorpin DKO cells were used as negative controls for RANBP9 and RANBP10 expression. The vertical lines on the right side of the panels represent blots from the same gel. Vinculin was used as a loading control for each blot. ImageJ version 1.53t (https://imagej.nih.gov/ij/) was used for the quantitation of the RANBP9 and RANBP10 band intensities normalized to the corresponding Vinculin intensity results, as shown in panel (**B**) for A549 iBP9 cells, in panel (**D**) for A549 iBP10 cells, and in panel (**F**) for A549 WT cells

When either RANBP9 shRNA-knockdown or CRISPR-KO NSCLC cells were generated, we previously reported that, in general, the expression of RANBP10 was greater than that in parental cells (Fig. 1A-1B, Supplementary Fig. 1 C) [8, 12, 44]. This observation, together with the high similarity between the two proteins, led us to hypothesize that the two paralogs may functionally compensate for each other’s absence. To further investigate this phenomenon in a short period of time, we engineered A549 parental (Scorpin WT) cells bearing Doxy-inducible safe-harbor gene integration of either RANBP9 or RANBP10, similar to what we previously reported in A549 Scorpin DKO cells (Supplementary Fig. 2 A). Time course experiments revealed that the protein expression of RANBP9 or RANBP10 was consistently upregulated from 6 to 48 h (Fig. 3A-D). When RANBP9 was induced, the amount of RANBP10 protein decreased accordingly (Fig. 3A-B). The reverse was also true. When the expression of RANBP10 was induced, the expression of RANBP9 was proportionally downregulated (Fig. 3C-D). GID8, MAEA, and WDR26 trended toward increased expression with the induction of either RANBP9 or RANBP10 (Fig. 3A-D; Supplementary Fig. 3 A). At the indicated times, Doxy treatment did not cause any appreciable change in Scorpin expression in the A549 parental line (Fig. 3E-F). The strong artificial increase in either the RANBP9 or RANBP10 transcript did not have a significant effect on the transcripts of their paralog or other CTLH complex members (Supplementary Fig. 3 C).

Together with previous observations, these results demonstrate that the expression of the RANBP9 and RANBP10 proteins is cross regulated in NSCLC cells and that their dynamic changes reciprocally affect each other’s protein expression in the short term. Overall, CTLH^BP9^ and CTLH^BP10^ are subject to balanced expression at the protein level.

### GID8 and RANBP9 are overexpressed, whereas RANBP10 is downregulated in NSCLC at both the RNA and protein levels

**Fig. 4:**
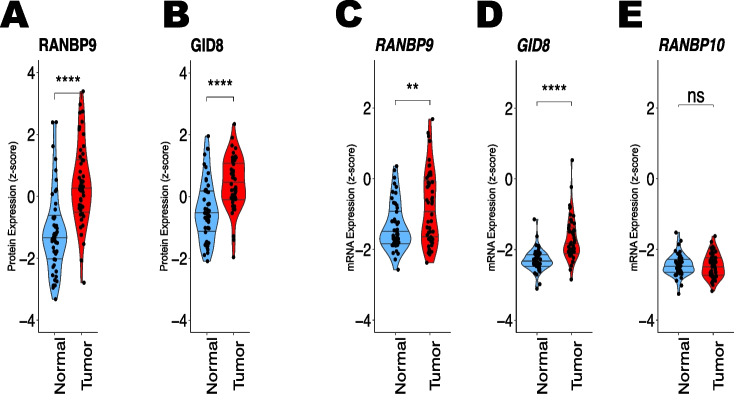
GID8 and RANBP9 are overexpressed, whereas RANBP10 is downregulated in NSCLC. **A**, **B** *GID8 and RANBP9 proteins are overexpressed in 50 NSCLC samples (OSU NSCLC collection).* RANBP9 and GID8 protein expression was quantified by assessing the WB band intensity normalized to that of vinculin in 50 NSCLC tumors with matched normal adjacent tissues, as shown in Supplementary Fig. 4A. **C**-**E** *GID8 and RANBP9 transcripts are overexpressed in 50 NSCLC samples (OSU NSCLC collection).* RNA was extracted from the same 50 frozen NSCLC tumors, and RT‒PCR was performed to measure the amount of RANBP9, GID8, and RANBP10 mRNA. The statistical significance of the differences was assessed via two-way ANOVA using RStudio. **** *p* < 0.001; *** *p* = 0.001; ** *p* = 0.01

The results above demonstrated that RANBP9 and RANBP10 acutely cross-regulate each other’s protein expression in vitro, without being correlated with their transcript levels. Next, we proceeded to establish the relevance of these findings in NSCLC patients. We previously reported that the protein levels of RANBP9 are higher both in NSCLC cells and in patient samples than in their normal counterparts [12, 44]. However, we could not unequivocally determine how pervasive the overexpression of the RANBP9 protein was because of the small number of NSCLC samples we had available at the time [44]. For this study, we acquired a collection of fifty (50) frozen NSCLC samples (T) with matched normal adjacent tissue (N), which included 16 adenocarcinoma (LUAD), 8 squamous carcinoma (LUSQ), 6 carcinoid, and other histotypes (OSU collection; Supplementary Table 1) samples. We measured the expression levels of the Scorpins and their binding partner GID8 via WB and RT‒PCR (Fig. 4A‒E; Supplementary Fig. 4 A; Supplementary Materials). We found that the RANBP9 and GID8 proteins were significantly more abundant in T vs. matched N samples (Fig. 4A-B; Supplementary Fig. 4 A). We also detected significant overexpression of GID8 and RANBP9 transcripts in T compared with N (Fig. 4C-D). On the other hand, we did not find a significant difference in the level of RANBP10 mRNA between the N and T stages (Fig. 4E). Regrettably, the low affinity/specificity of the commercially available antibody did not allow reliable quantitation of the RANBP10 protein. To corroborate our findings concerning the overexpression of RANBP9 and GID8 in NSCLC, we analyzed the level of expression of the CTLH genes in the publicly available TCGA collection (www.cbioportal.org) of lung cancer samples (T) with paired normal adjacent tissue (N) both squamous cell carcinoma (LUSQ) and adenocarcinoma (LUAD) samples. As shown in Supplementary Fig. 4B-C, RANBP9 and GID8 (a.k.a. C20orf11) were significantly higher in T than in N both in LUAD and LUSQ. Interestingly, RANBP10 mRNA levels were significantly lower in the T dataset than in the N dataset in both the LUAD and LUSQ datasets. Overall, the TCGA collection of NSCLC data revealed that RANBP9 and GID8 mRNAs were overexpressed, whereas RANBP10 transcript levels were decreased in both LUAD and LUSQ samples compared with those in T vs N samples.

Collectively, these results show that both the transcripts and proteins of GID8 and RANBP9 are upregulated in tumors of various histotypes in NSCLC patients. On the other hand, RANBP10 mRNA is expressed at low levels.

To further investigate Scorpin expression in NSCLC, we sought to analyze the transcriptomic and proteomic data characterized by the Clinical Proteomic Tumor Analysis Consortium (CPTAC) for lung adenocarcinoma (LUAD) and squamous lung cancer (LUSQ) recently published and available at kb.linkedomics.org [49–51].

**Fig. 5:**
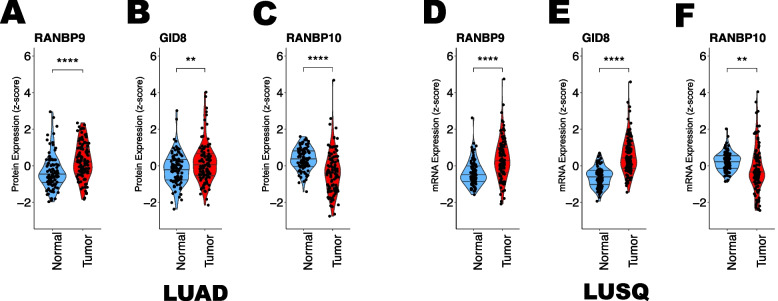
GID8 and RANBP9 are overexpressed, whereas RanBP10 is downregulated in the CPTAC NSCLC collection of NSCLC tumors compared with matched normal adjacent tissue. Quantitation of protein expression from the CPTAC LUAD dataset revealed that both the RANBP9 (**A**) and GID8 (**B**) proteins are significantly overexpressed, whereas the RANBP10 (**C**) protein is significantly downregulated in LUAD tumors compared with normal adjacent tissues. The quantification of protein expression from the LUSQ data collection revealed that both the RANBP9 (**D**) and GID8 (**E**) proteins were significantly overexpressed, whereas the RANBP10 (**F**) protein was significantly downregulated in LUSQ tumors compared with normal adjacent tissues. The reported data were downloaded from https://kb.linkedomics.org/. The statistical analysis was performed via RStudio. The statistical significance of the differences was assessed via two-way ANOVA using RStudio. **** *p* < 0.001; *** *p* = 0.001; ** *p* = 0.01

Consistent with our observations in the OSU collection of NSCLC samples analyzed by WB (Fig. 4A-B; Supplementary Fig. 4 A), the RANBP9 and GID8 proteins were significantly upregulated in both LUAD and LUSQ samples (Fig. 5A, B, D, E). On the other hand, the RANBP10 protein was significantly underexpressed compared with that in normal matched controls (Fig. 5C, F). The expression of mRNAs exhibited highly similar patterns (Supplementary Fig. 5G-L). Although RANBP10 is expressed at lower levels on average, some tumors presented relatively high RANBP10 expression compared with normal controls. These latter cases appear to have correspondingly higher levels of GID8, as indicated by the significant positive correlation between GID8 and RANBP10 in both the LUAD and LUSQ datasets (Supplementary Fig. 5 A, B, D, E). We also determined that in both LUAD and LUSQ, the maximum values of either RANBP9 or RANBP10 were much more strongly correlated with GID8 protein levels than either of the two Scorpins alone were (Supplementary Fig. 5 C, F). The latter result is concordant with the stoichiometric relationship observed in our cell line experiments, suggesting that the predominant expression of one member suppresses the expression of its counterpart and that both RANBP9 and RANBP10 can stabilize the CTLH, protecting GID8 from degradation.

Taken together, these data show that RANBP9 expression dominates RANBP10 expression at both the mRNA and protein levels, indicating that the chronic changes in CTLH protein levels are mediated by adjustments in transcript levels differently from those observed in cell lines in short-term experiments. Moreover, the expression of both RANBP9 and RANBP10 was positively correlated with the GID8 level.

### RANBP9 and RANBP10 correlate with significantly different proteomes in NSCLC patient tumors

**Fig. 6:**
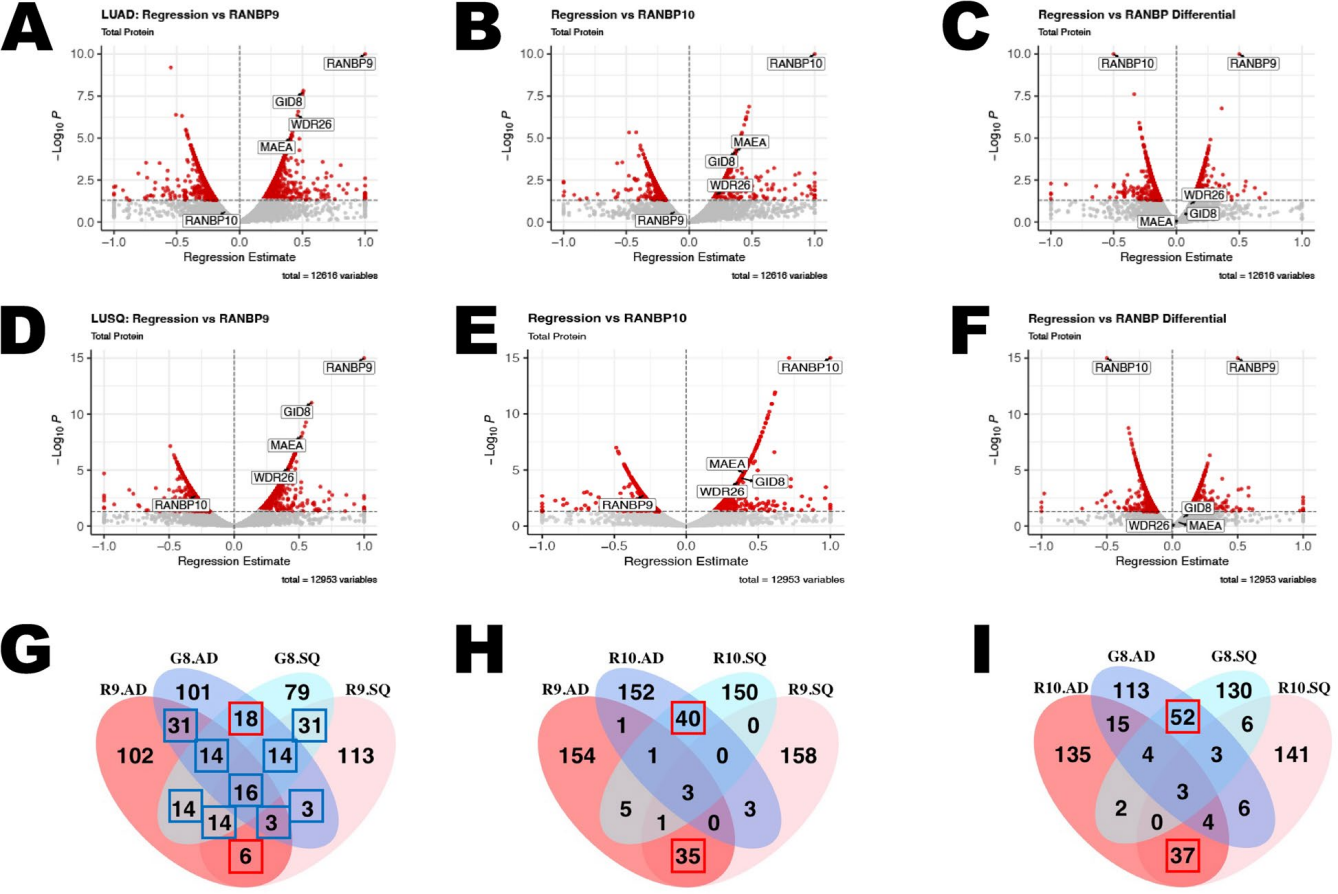
RANBP9 and RANBP10 correlate with significantly different proteomes in NSCLC patient tumors. Volcano plots illustrating regression analysis of proteins related to RANBP9 (**A**, **D**), RANBP10 (**B**, **E**) or DBP expression (differences in Z scores for RANBP9 minus RANBP10) (**C**, **F**) in the LUAD and LUSQ CPTAC collection, respectively. Protein expression was converted to a Z score, and regression analysis was performed in RStudio. Unadjusted log10-transformed *p* values on the y-axis are plotted against regression estimates. Values exceeding the plotted ranges are shown at the corresponding maximum or minimum values. The CTLH family members RANBP9, RANBP10, GID8, MAEA, and WRD26 are labeled. Venn diagrams showing the overlap between the top 200 proteins for associations with RANBP9 vs GID8 (**G**), RANBP9 vs RANBP10 (**H**), and RANBP10 vs GID8 (**I**) for the CPTAC LUAD and LUSQ datasets. For RANBP10 (R10) vs RANBP9 (R9) (**H**) and RANBP10 (R10) vs GID8 (G8) (**I**), the overlapping proteins favor R10.AD ~ R10. SQ (where AD = LUAD and SQ = LUSQ), R9.AD ~ R9. SQ, and G8.AD ~ G8.SQ. The similarity is minimal when R10 ~ G8 or especially R10 ~ R9 are considered. The opposite is observed for RANBP9 vs GID8 (**G**), in which many overlapping proteins are shared between R9 ~ G8 (in blue boxes) both in LUAD and LUSQ, and relatively fewer proteins are unique to R9.AD ~ R9. SQ or G8.AD ~ G8.SQ. In red boxes, proteins that are correlated with each of the three proteins common between the LUAD and LUSQ collections are shown. The statistical analysis was performed via RStudio; the top 200 associated proteins were ranked by *p* value from linear regression with the indicated CTLH member in the respective tumor cohort. The data were downloaded from https://kb.linkedomics.org/

Having established that RANBP9 and GID8 are upregulated while RANBP10 is downregulated or expressed at low levels in NSCLC patients, we next proceeded to assess their correlations with the global proteome in the CPTAC LUAD and LUSQ datasets. The regression analysis of the absolute expression of both Scorpins and their differential expression (RANBP9 minus RANBP10 = delta [DRANBP] expression) revealed that RANBP9 and RANBP10 expression correlated negatively and positively with hundreds of other proteins both in LUAD (Fig. 6A*‒C*) and LUSQ (Fig. 6D‒F; Supplementary Material). As mentioned, both RANBP9 and RANBP10 were positively correlated with GID8 (Fig. 6A, B, D, E; Supplementary Fig. 6A-B) but negatively correlated with each other (Fig. 6A-F). In both LUAD and LUSQ, the list of proteins positively and negatively correlated with RANBP9 was significantly different from that associated with RANBP10. A selection of the top 200 proteins expressed in correlation with one of the three CTLH members in both types of tumors revealed that the overlapping proteins favor the comparisons RANBP10 LUAD vs RANBP10 LUSQ, RANBP9 LUAD vs RANBP9 LUSQ, and GID8 LUAD vs GID8 LUSQ, suggesting strongly concordant effects on tumor biology across distinct tumor histologies (Fig. 6G, I). RANBP9-associated proteins highly overlap with GID8-associated proteins (Fig. 6G, blue boxes), and relatively fewer proteins are unique to one group (Supplementary Material). Conversely, only limited similarity was observed when comparing RANBP10 vs GID8, especially when comparing RANBP9 to RANBP10.

Collectively, these results indicate that RANBP9 and RANBP10 expression in NSCLC correlates with different proteins because they both positively correlate with GID8, thus suggesting a partially different functional role for the two paralogs.

### RANBP9 expression is associated with increased proliferation in NSCLC

To gain biological insights into the proteomes potentially regulated by RANBP9 and RANBP10 in NSCLC, we performed two independent analyses to search for gene sets, pathways, and biological processes. First, we performed gene set enrichment analysis (GSEA) via the LinkedOmics website (kb.linkedomics.org) [49–51], which queries associations with the publicly available WEB-basedGEne SeT AnaLysis Toolkit (WebGestalt: webgestalt.org) (Supplementary Fig. 7A-H). Second, we used the list of proteins that were positively or negatively associated with RANBP9 or RANBP10 in the CPTAC LUSQ and LUAD collections (Supplementary Material) to perform Metascape analyses (https://metascape.org) (Supplementary Fig. 7I‒P) [52].

Both the WebGestalt and the Metascape results indicated that RANBP9 and RANBP10 have strong positive associations with gene sets related to all the different steps of RNA metabolism in both LUSQ and LUAD (Supplementary Fig. 7A-P). The results also revealed that RANBP9 and RANBP10 were negatively correlated with processes related to different aspects of the immune response, both innate and adaptive, together with endocytosis, vesicle and membrane trafficking, and cell adhesion processes (Supplementary Fig. 7I-P). However, both the GSEA and the Metascape analysis also revealed differences between RANBP9 and RANBP10. GSEA revealed that RANBP9 was positively associated with “DNA replication” in both LUSQ and LUAD, whereas RANBP10 was not (Supplementary Fig. 7A-B). In the Metascape analysis, RANBP9 expression was strongly associated with terms related to cell proliferation, such as “cell cycle” (R-HSA-1640170), “DNA metabolic process” (GO:0051052), “mitotic cell cycle” (GO:0000278) or “mitotic cell cycle process” (GO:1,903,047) or “mitotic G2-G2M phases” (R-HSA-453274), “cell cycle checkpoints” (R-HSA-69620), “regulation of cell cycle process” (GO:0010564), “DNA replication” (GO:0006260 and WP466), “regulation of DNA replication” (GO:0007265), and “S-phase” (R-HSA-69242) (Supplementary Fig. 7I-J). RANBP10 was associated with “regulation of cell cycle process” (GO:0010564) in LUAD and with “cell cycle” (R-HSA-1640170) in LUSQ, although the statistical significance of these latter associations was markedly lower.

Collectively, these results show that in NSCLC tumors, both the CTLH^BP9^ and the CTLH^BP10^ configurations are potentially involved in the regulation of biological processes such as multiple steps of RNA metabolism, but they also have distinct preferential associations with other biological processes such as cell proliferation, which was found to be strongly associated with the increased protein ratio RANBP9/RANBP10.

**Fig. 7:**
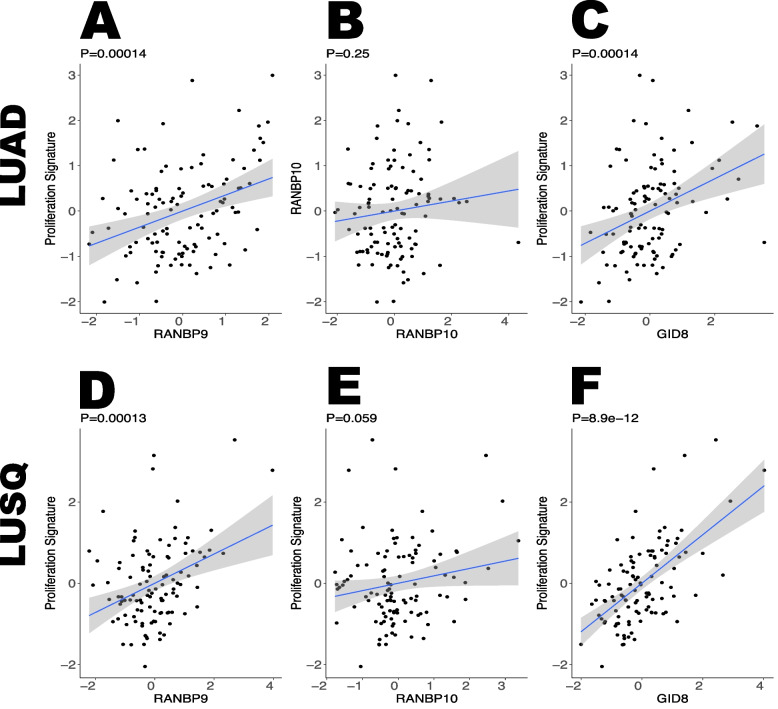
RANBP9 expression is associated with cell proliferation-associated proteins (PPAs) in NSCLC. A 282-protein proliferation signature was used to establish the correlation between RANBP9 and proliferation in LUAD (**A**) and between LUSQ (**D**), RANBP10 in LUAD (**B**) and LUSQ (**E**), and GID8 in LUAD (**C**) and LUSQ (**F**). The statistical analysis was performed via RStudio; the proliferation score was calculated as the mean Z score for the corresponding 282 proteins and was plotted against the CTLH member as indicated. *P* value results from linear regression. The data were downloaded from https://kb.linkedomics.org/

To corroborate the preferential association between cell proliferation and RANBP9 expression in comparison with RANBP10, we selected a recently published protein proliferation signature [53] and analyzed its correlation with the two Scorpins and GID8 in both the CPTAC LUAD and LUSQ datasets (Fig. 7A-F). Even if both RANBP9 and RANBP10 expression was positively correlated with GID8 expression (Fig. 6A, B, D, E; Supplementary Fig. 6A-B), the results clearly revealed that RANBP9 and GID8 expression was positively correlated with the proliferation signature in both LUAD and LUSQ, whereas RANBP10 expression was not (Fig. 7A-F).

Taken together, these results indicate that the CTLH complexes formed by RANBP9 or RANBP10 are associated with a variety of fundamental biological processes and proteins, where a relatively high RANBP9/RANBP10 ratio is positively correlated with proliferation in NSCLC tumors.

### Compared with RANBP9, the acute overexpression of RANBP10 causes different changes in the NSCLC proteome, downregulating several proliferation-associated proteins

**Fig. 8:**
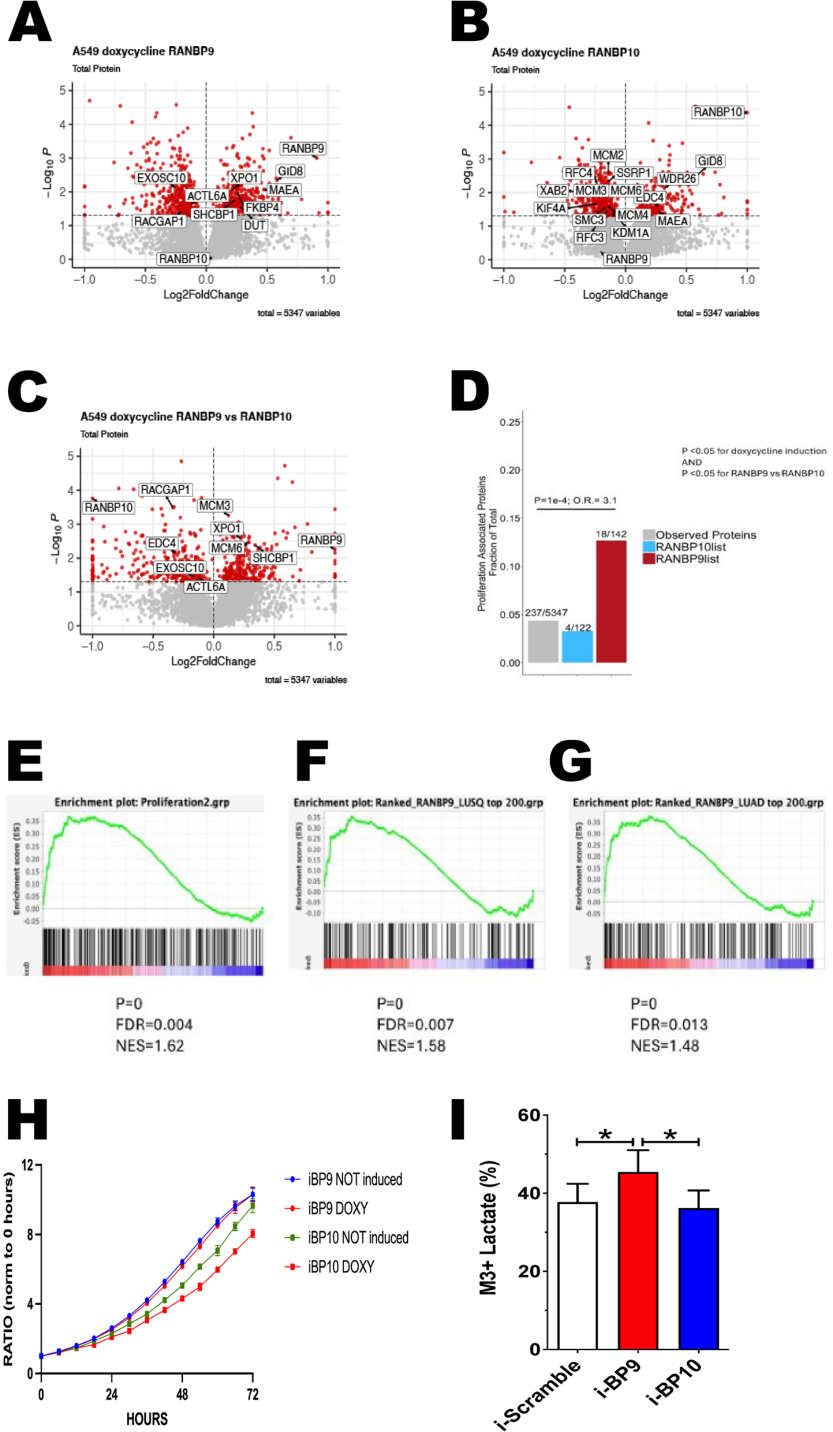
Compared with RANBP9, the acute overexpression of RANBP10 causes different changes in the NSCLC proteome, downregulating several PPAs and reducing cell proliferation. Volcano plots illustrating the proteins whose expression significantly changed in relation to that of RANBP9 (**A**), RANBP10 (**B**), and DBP (**C**) in iA549 cells. Red indicates proteins whose statistical significance is _log10_ *p* ≤ 1.300. CTLH complex members and proliferation-associated proteins are labeled. **D** Bar graph reporting the number of proliferation-associated proteins (PAPs [53]) found in total (gray bar), in the list of proteins positively associated with RANBP10 (blue bar), and in the list of proteins positively associated with RANBP9 (red bar). The results of Fisher’s test revealed a statistically significant increase in the number of PAPs positively associated with RANBP9 (*p* = 0.0001), with an odds ratio (OR) = 3.1. Proteins were considered to be positively associated with RANBP9 or RANBP10 if they were significantly different between the Doxy-treated and control conditions and significantly different in expression vs the opposite Scorpin member in the Doxy-treated condition, each with *P* < 0.05 according to Student’s t test. Proteins were ranked for differential expression of RANBP9 vs RANBP10 induction in A549 cells after Doxy treatment for 24 h, and gene set enrichment analysis was performed for the indicated gene set: EPAP [53]. signature; **F** top 200 proteins associated with RANBP9 in CPTAC LUSQ; **G** top 200 proteins associated with RANBP9 in CPTAC LUAD. P = p value; FDR = false discovery rate; NES = normalized enrichment score. Plots generated via GSEA_4.3.2. **H** Scorpin WT A549 iBP9 (round dots) and iBP10 (square dots) cells were seeded onto 96-well plates and exposed to 1 mg/mL Doxy (red) or not (iBP9 blue and iBP10 green). Cell growth was measured via microscopy-based technology with an IncuCyte™ instrument. Cell growth was normalized to that on day 0, and each dot represents the average of 4 wells. I) RANBP9 but not RANBP10 promotes the conversion of [U-13C]-glucose into [U-13C]-lactate. A549 cells overexpressing either RANBP9 or RANBP10 were grown in DMEM/F-12 supplemented with 5 mM [U-13C]-glucose and 2 mM glutamine. After 6 h of incubation, metabolites were extracted, dried and derivatized before being analyzed by GS-MS. Data are presented as the mean ± SD of 5 independent experiments. The statistical significance is indicated as *P < 0.05

Next, to gain mechanistic insight into the effects of RANBP9 overexpression versus RANBP10 overexpression, we aimed to establish whether the increase in RANBP9 or RANBP10 levels differentially affected iA549 cell proliferation. We previously reported that ablation of RANBP9 in NSCLC cells caused a modest but consistent reduction in cell proliferation [12]. In contrast, the downregulation of RANBP9 in HEK293 cells increased cell proliferation, and the silencing of RANBP10 was found to reduce glioblastoma cell proliferation [37, 54]. However, our A549 WT cell lines, where either RANBP9 (Scorpin WT A549 iBP9) or RANBP10 (Scorpin WT A549 iBP10) are induced without the constitutive ablation of endogenous genes, provide a tool that avoids artifacts due to in vitro cell adaptation previously observed when manipulating CTLH proteins and better mimics human NSCLC tumors [2, 3] (Fig. 3). We treated Scorpin WT iBP9 and iBP10 cells with Doxy for 24 h. Quadruplicate samples were collected and analyzed via tandem mass spectrometry via an isotopic labeling approach (Supplementary Fig. 8 A). For the CPTAC data collection, we considered the effects of RANBP9 induction, RANBP10 induction, and DRANBP separately (Fig. 8A-C).

The overexpression of the two Scorpins caused significant global proteome changes (Fig. 8A-C; Supplementary Material). RANBP9 can affect the expression levels of other proteins both negatively and positively [8, 17, 23, 55–58]. After the induction of RANBP9 in A549 iBP9 cells, 396 and 394 proteins correlated positively and negatively with RANBP9 expression (_log10_ p ≤ 1.3), respectively (Fig. 8A). When RANBP10 was induced in A549 iBP10 cells, 229 and 404 proteins correlated positively and negatively with RANBP10 levels, respectively (_log10_ p ≤ 1.3) (Fig. 8B). In both induced cell lines, the amounts of GID8 and MAEA were positively correlated with either RANBP9 (Fig. 8A) or RANBP10 (Fig. 8B), which is in line with our previous results. When considering the expression of DRANBP, 288 proteins were found to be positively correlated with a higher DRANBP, and 293 proteins were positively correlated with a lower DRANBP _(log10_ p ≤ 1.3) (Fig. 8C; Supplementary Material). These results are in line with the observations in the NSCLC CPTAC data, where the expression of the two paralogs correlated with only partially overlapping enriched proteomes, while both correlated positively with GID8 and other CTLH members (Fig. 6A-I).

Proteins that were differentially expressed when RANBP9 was overexpressed were significantly similar to RANBP9-associated proteins in both LUSQ and LUAD (Fig. 8D). This similarity was in part due to an enrichment of proliferation-associated proteins (Fig. 8A-C, E–G), in agreement with the positive association of RANBP9 expression with proliferation observed in CPTAC patient tumors. However, our experiments also revealed that the overexpression of RANBP10 clearly downregulated the expression of proliferation-associated proteins (PPAs) (Fig. 8B; Supplementary Fig. 8B-C). RANBP9 loss of function or downregulation in NSCLC cells causes a modest but consistent reduction in cell proliferation [12, 44]. Therefore, we investigated the effect of RANBP10 overexpression on iA549 cell proliferation. We again used iA549 WT iBP9 and iBP10 cells treated with or without Doxy and monitored cell growth for three days. While the overexpression of RANBP9 did not significantly affect cell growth, the overexpression of RANBP10 caused a modest but significant decrease in the growth rate, which became evident after 24 h (Fig. 8H). These results indicate that in iA549 cells, an increase in RANBP10 (a lower RANBP9/RANBP10 ratio) has a “braking effect” on cell proliferation.

NSCLC is considered a high glycolytic tumor [59]. Due to the hypothesized involvement of RANBP9 in the regulation of glycolysis [55], we also performed experiments aimed at measuring the production of lactate derived from [U-13C]-glucose. The overexpression of RANBP9 in A549 caused a 19% increase of lactate deriving from glucose in comparison with the inducible scramble control. The overexpression of RANBP10 did not cause any change (Fig. 8I). This result indicate that the increase of RanBP9 in NSCLC not only is functional to cell proliferation, but also promotes glycolysis.

Collectively, these results show that the artificial overexpression of the two Scorpins modulates distinct groups of proteins. Moreover, the overexpression of the CTLH^BP10^ complex downregulates PPAs, leading to a measurable decrease in the growth rate. These observations are also consistent with the association of RANBP9 with an increased proliferative phenotype in CPTAC NSCLC tumors and decreased proliferation when RANBP9 is silenced or ablated.

### Compared with RANBP9, the acute overexpression of RANBP10 causes different changes in the A549 ubiquitylome, which includes proliferation-associated proteins

**Fig. 9:**
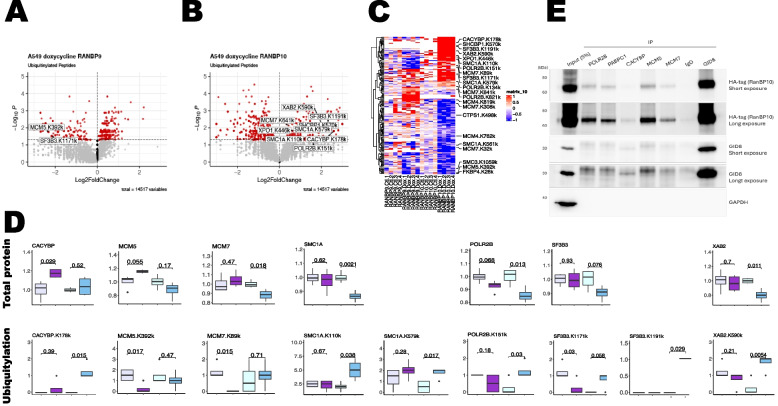
Compared with RANBP9, the overexpression of RANBP10 causes different changes in the NSCLC ubiquitylome, which includes PPAs. Volcano plots illustrating the proteins whose expression significantly changed in relation to that of RANBP9 (**A**) or RANBP10 (**B**). Red indicates proteins whose statistical significance is _log10_ *p* ≤ 1.300. Specific ubiquitylations of PPAs of interest are labeled. **C** Heatmap depicting ubiquitylations (*p* = 0.05) observed in proteins differentially expressed between iA549 iBP9 and iBP10 cells (DBP *p* = 0.05 and increased under RANBP9 conditions) upon treatment with Doxy for 12 h. Peptides corresponding to PAPs [53] are labeled. **D** Illustrative plots of total protein changes (top panels) and their specific ubiquitylations (bottom panels) observed with the opposite pattern of seven selected PPAs. Plots were generated via RStudio. **E** PAPs coimmunoprecipitate with RANBP10. HA-tagged RANBP10 expression was induced using Doxy at 10 ng/mL. After 24 h 1 mg of total cell lysate was immunoprecipitated by primary antibodies to pull-down POLR2B, PABPC1, CACYBP, MCM5, and MCM7. Rabbit IgG pull-down was used as negative control and GID8 pull-down was used as positive control. The presence of RANBP10 was detected by HRP-conjugated anti-HA. GAPDH is used as loading control

All observations made thus far in NSCLC patients and cell lines have indicated that the CTLH^BP9^ and CTLH^BP10^ complexes coexist in a tightly regulated balance and that the functional effects of these two CTLH configurations are different, especially when considering cell proliferation. Next, we aimed to identify potential mechanistic candidates to guide future studies by assessing the effects of Scorpin manipulation on the A549 ubiquitylome. We repeated the Scorpin WT iA549 iBP9 and iBP10 cell induction and collected samples after proteasomal inhibition with MG132. Quadruplicate samples were collected and processed to enrich for ubiquitylated peptides with KGG remnants as proxies for ubiquitylation (Supplementary Fig. 9 A). We found that RANBP9 overexpression was positively correlated with 453 KGGs and negatively correlated with 436 KGGs (_log10_ p ≤ 1.3). On the other hand, the induction of RANBP10 was positively correlated with 1,765 KGGs and negatively correlated with 598 peptides with KGGs (Fig. 9A-C; Supplementary Material). Considering the expression of DRANBP, 1,559 genes were positively correlated with it, and 1,611 genes were negatively correlated with it (Fig. 9C).

These results clearly demonstrated that the upregulation of RANBP9 has significantly different effects on reshaping the ubiquitylome of iA549 cells than the increase in RANBP10 does.

We found that proteins displaying more than one KGG peptide, including RANBP9 and RANBP10, were significantly enriched upon induction with the Scorpins themselves (Supplementary Material). We constructed lists of ubiquitylated proteins associated either positively or negatively with RANBP9 or RANBP10 (Supplementary Material) and performed a Metascape analysis to assess which gene sets, pathways, and biological processes were potentially perturbed upon manipulation of the Scorpin expression levels (Supplementary Fig. 9B-E).

“Metabolism of RNA” (R-HSA-8953854) was one of the most significantly enriched terms in all 4 different groups, further confirming that the two Scorpins participate together in the regulation of the transcriptome. The term “cell cycle” (R-HSA-164070) was among the top two enriched terms in the list compiled with ubiquitylated proteins associated either positively or negatively with RANBP10 (Supplementary Fig. 9D-E). Importantly, the same “cell cycle” (R-HSA-164070) term was not significantly enriched in the Metascape analysis of ubiquitylated proteins positively or negatively associated with RANBP9 (Supplementary Fig. 9B-C). However, other terms related to cell proliferation, such as “cell cycle, mitotic” (R-HSA-69278″) and “mitotic cell cycle process”, were significantly enriched with the proteins negatively associated with RANBP9.

Overall, these results indicate that a change in the balance between CTLH^BP9^ and CTLH^BP10^ at 24 h results in hundreds of ubiquitylation changes in NSCLC cells, potentially fine-tuning a wide variety of key biological processes, including RNA processing and cell proliferation.

Analysis of the ubiquitylome revealed that several PAPs had significantly different ubiquitylations when RANBP9 or RANBP10 was overexpressed in iA549 cells (Fig. 9A-C). To prioritize candidates most likely to be functionally relevant, we focused on the intersection of differentially ubiquitylated peptides whose total protein expression was also significantly altered by altered Scorpin expression. To broaden the list of putative candidates, we used a less stringent statistical cutoff for the total protein associations, requiring both a difference in Doxy induction with *p* < 0.1 and a difference in the RANBP9 > RANBP10 effect with *P* < 0.1; for the ubiquitylation effect, *p* < 0.05 was used for Doxy vs the control in either RANBP9 or RANBP10 induction.

As in our earlier results (Fig. 8D), a higher ratio of RANBP9 > RANBP10 remained significantly enriched for PAPs with the use of relaxed statistical cutoffs (Supplementary Fig. 9 F; 27 of 337; odds ratio 1.8, *p* < 0.01). Among the 337 RANBP9-associated proteins, 43 had at least one ubiquitylation site whose expression was increased by RANBP10 induction, and this subset presented the greatest enrichment of proliferation-associated proteins (8 of 43; odds ratio 4.9, *P* < 0.001). These differentially expressed proteins and their corresponding ubiquitylation sites putatively represent candidate substrates that may link the CTLH E3 ubiquitin ligase activity with the regulation of cell growth and proliferation.

Ultimately, we found that seven proliferation-associated proteins displayed significantly different specific ubiquitylation events that could explain the different levels of expression observed in the analysis of the proteome (Fig. 9D). This restricted list included the two members of the replisome MCM5 and MCM7, the calcium binding protein CACYBP1, which regulates replisome functions, the cohesin SMC1A, the core component of the RNA polymerase II POLR2B, the splicing factor SF3B3, and XAB2. Among these, CACYBP1, MCM5, MCM7, SMC1A, and SF3B3 were previously reported to be bona fide CTLH interactors in at least two different studies [36, 55, 60–62]. However, we generated A549 inducible cell lines similar to the ones used above but with the difference that RANBP9 is tagged with V5 and RANBP10 is tagged with HA to validate the interaction of selected targets. Results show that RanBP10-HA is pulled-down by POLR2B, SF3B3, CACYBP1, MCM5, and MCM7 (Fig. 9E). Similar results were obtained with RANBP9-V5 (Supplementary Fig. 9G).

These results demonstrate that the overexpression of RANBP9 or RANBP10 changes the total amount and ubiquitylation pattern of proteins critically involved in cell proliferation, such as members of the replisome and cohesins [63, 64], with prioritized candidates that warrant further mechanistic validation in future studies.

### RANBP9 regulates key oncogenic pathways preferentially at protein or at transcript level

**Fig. 10:**
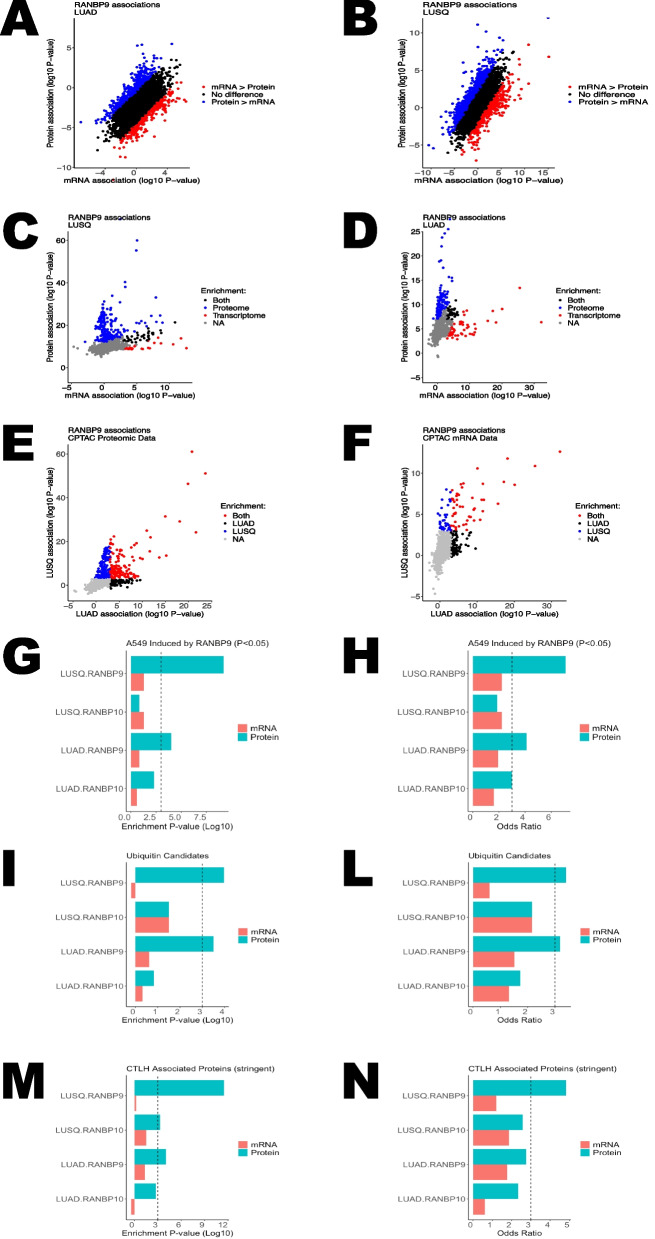
RANBP9 regulates key oncogenic pathways preferentially at protein or at transcript level. Analysis of individual mRNAs vs. proteins from CPTAC LUAD (**A**) and LUSQ (**B**) showing the linear regression vs. RANBP9 protein. The *p*-value of two orders of magnitude established arbitrarily using the numeric difference defines genes having a stronger mRNA (red) or protein (blue) association with RANBP9. Pathway analysis in LUAD (**C**) and LUSQ (**D**) shows that certain pathways have a stronger RANBP9 association with proteomic (blue) rather than transcriptomic (red) data while other have the opposite trend. The comparison of proteomic of LUAD vs LUSQ (**E**) and transcriptomic (**F**) analyses shows a strong concordance between the two different CPTAC lung cancer collections. The enrichment analysis using a list of previously reported putative CTLH-interactors also shows enhanced significance indicated as enrichment value (**G**) or odds ratio (**H**) for RANBP9 associations from proteomic data, with no significant association with mRNA data. Proteins used for this analysis were induced by RANBP9 over RANBP10 in A549 with a *p*-value cutoff of 0.05. A similar analysis using proteins showing differential ubiquitylations (**I**, **L**) or previously reported as putative CTLH interactors in at least two different studies (**M**, **N**) when RANBP9 was induced in comparison to RANBP10 also show an increased significance for RANBP9 associations from proteomic data, with no significant association with the mRNA data expressed as enrichment value (**I**, **M**) or odds ratio (**L**, **N**)

Finally, we aimed to better capture the long term-implications of our findings and to better identify the differential pathways most proximally linked to CTLH functional activity considering that the CTLH complex is intimately involved in ubiquitylation and post-translational regulation of target protein expression. To this aim, first we performed the comparative analysis of transcriptome vs proteome in NSLC patients. Using integrated data from the CPTAC LUAD and LUSQ datasets, we determined the association of mRNA or protein expression compared to RANBP9 protein expression by linear regression, identifying targets with significant skewing in transcriptomic vs proteomic regulation defined as a log10 difference in *P*-value greater than 3.0. Overall, 6.6% of genes in the LUAD dataset and 11.1% of genes in LUSQ met this criteria (Fig. 10A,B). Analysis of association with RANBP10 levels showed fewer skewed relationships (3.2% and 6.6% respectively; Supplementary Fig. 10 A,B).

We next leveraged the Molecular Signatures database to infer functional interpretation of these differences. Enrichment analysis of Hallmark, KEGG, Reactome, and Transcription Factor gene sets were used and applied separately to proteomic vs transcriptomic RANBP9 rank lists, revealing significant distinctions in pathway regulation based on the type of analytic data (Fig. 10* C,D*). These differential effects were observed in both the LUAD and LUSQ datasets; however, proteomic associations in LUAD showed high similarity to those in LUSQ, and mRNA to mRNA comparisons were likewise highly concordant (Fig. 10E,F). The highest ranked pathways (Supplementary Fig. 10G) from the analysis of the proteome data implies a significant association of RANBP9 expression with upregulation of spliceosome and mRNA processing pathways, which was much less evident in the mRNA data. Conversely, the transcriptome data demonstrated a link between RANBP9 and mitotic and G2M checkpoint gene expression. The analysis also showed that RANBP10 expression was not significantly linked to these pathways, because it showed lower enrichment scores for pathways in general, and did not reveal any significant concordance between LUAD and LUSQ datasets (Supplementary Fig. 10 A,F).

Next, we turned our attention to overlap analysis of RANBP9 or RANBP10 associations with user defined genesets derived from our proteomic characterization of A549 derivatives expressing RANBP9 vs RANBP10 under control of a Doxy-inducible promoter, as well as a curated list of genes previously reported as putative CTLH targets or associated genes. Human lung cancer proteins showing positive correlation with RANBP9, for both LUAD and LUSQ cohorts, demonstrated significant positive enrichment scores for RANBP9 associated proteins in A549 (Fig. 10G,H), candidates with differential ubiquitylation (Fig. 10I,L), and literature-defined CTLH associated gene lists (Fig. 10M,N). In each case the enrichment scores favored associations with RANBP9 compared to RANBP10, and were observed for associations derived from proteomic data, but not mRNA.

In particular, we identified SF3B3 and XAB2 as CTLH-regulated candidates within spliceosome and mRNA metabolism pathways. After Doxy induction, an increase in ubiquitylation at K1171 and K1191 (Fig. 9D) was detected only in for RANBP10 induced derivative, and was associated with decrease in total SF3B3 protein expression; similarly, XAB2 showed increase at ubiquitylation at K590 alongside decrease in XAB2 protein expression in the RANBP10 induced derivative (Fig. 9D). Of note, SF3B3 has been previously identified as RANBP9 interactor and a putative functional target of the CTLH complex [60].

These results show how the balance between the CTLH^BP9^ and the CTLH^BP10^ can exert acute protein-mediated effects and long-term regulatory consequences on the transcriptome.

## Discussion

Despite significant advances in patient survival, NSCLC remains the deadliest cancer in the United States [65, 66]. A better understanding of the pathogenesis of the disease is needed to design more efficacious biology-driven treatments [67–69].

The present work illustrates the role of two variants of the CTLH complex, an E3 ligase that is emerging as a central node connecting cell signaling and metabolism, in NSCLC [2, 19–21, 23].

Here, we show that RANBP9 and RANBP10, also called Scorpins [34], work in concert to modulate the ubiquitylation output of the CTLH complex in NSCLC. First, we demonstrated that the CTLH complex coexists in two different configurations, one based on the scaffold provided by RANBP9 (CTLH^BP9^) and the other built on RANBP10 (CTLH^BP10^). Both complexes are expressed in normal lung and NSCLC tumors (Fig. 1A-C, Supplementary Fig. 1B-C, Fig. 4A-E, Fig. 5A-F, Supplementary Fig. 5A-L, Fig. 6A-F). In agreement with previous observations demonstrating the existence of both CTLH^BP9^ and CTLH^BP10^ during erythroid maturation, RANBP9 or RANBP10 were independently sufficient to protect their binding partner GID8 from proteolysis and stabilize the core on which the complex is formed (Fig. 1A-C, Supplementary Fig. 1 A, Fig. 2, Supplementary Fig. 2 A, B) [35].

CTLH^BP9^ and CTLH^BP10^ complexes acutely cross-regulate each other at the protein level. An artificial increase in the amount of RANBP9 caused a decrease in RANBP10, and vice versa, the forced increase in RANBP10 caused a proportional decrease in RANBP9 (Fig. 3A-D; Supplementary Fig. 3 A, B). Notably, upon acute induction of the expression of RANBP9 or RANBP10, the transcripts of the uninduced paralog did not appreciably change within the short period we analyzed (Supplementary Fig. 3 C). Similarly, in single-KO NSCLC cells, the paralog transcript level did not significantly increase, whereas the protein amount did (Supplementary Fig. 1B-C). In Scorpin double-KO (DKO) NSCLC cells, the disappearance of GID8 and MAEA, on the one hand, and the increase in MKLN1 on the other hand, are not in line with the levels of their relative transcripts (Supplementary Fig. 1B-C). It is not uncommon to find a poor correlation between the mRNA and protein levels of CTLH family members and proteins involved in proteostasis in general [12, 23, 70–73]. However, albeit not in all tumors taken singularly, the overall increase in RANBP9 protein in NSCLC masses corresponded to an overall increase in the RANBP9 transcript, and the overall decrease in RANBP10 was also consistently observed at both the protein and mRNA levels (Fig. 4A-D, Fig. 5A-F; Supplementary Fig. 5G-L). Therefore, we can conclude that while acute changes in RANBP9 and RANBP10 cause changes in protein expression, long-term changes in Scorpin mRNA expression are involved in NSCLC tumorigenesis.

Existing evidence indicates that RANBP9 and RANBP10 originated from the duplication of the ancestral yeast gid1 gene and that they can partially compensate for each other’s absence [23, 74, 75]. In contrast, there is evidence that these two proteins may have opposite functions [9, 76]. Hence, the two paralogs should be considered partially antagonistic, similarly to other proteins that originated from evolutionary duplications [77]. In line with this concept, here, we show that the combined targeting of both Scorpins in NSCLC impairs the formation of functional CTLH complexes (Fig. 1A-C; Supplementary Fig. 1 A) [24, 29]. We also show that, compared with RANBP10 induction, the controlled overexpression of RANBP9 in iA549 cells caused changes in the expression and ubiquitylation of a significantly different number of proteins (Fig. 8A-D, Supplementary Fig. 8A-C, Fig. 9A-C, Supplementary Fig. 9B-F). The dynamics observed in vitro (Fig. 3A) were consistent with the observations in CPTAC patients, where RANBP9 was overexpressed, whereas RANBP10 was maintained at low levels (Fig. 6A-F). In vivo and in vitro, the expression of either RANBP9 or RANBP10 was positively correlated with that of other members of the CTLH complex, but the two paralogs were negatively correlated with each other (Fig. 6A-C, Fig. 8A-B). We identified proteins affected by the overexpression of RANBP9 and/or RANBP10 in both iA549 (Fig. 8A-B) and CPTAC patients (Fig. 6A-F). Collectively, our data suggest that, in NSCLC, the increase in RANBP9 together with the decrease in RANBP10 works in concert to determine proteome and ubiquitylome changes that potentially directly and indirectly modulate many other proteins (Supplementary Fig. 7A-P).

The lists of proteins whose total amount or ubiquitylation was altered depending on the expression of the two Scorpins in CPTAC NSCLC patients and in iA549 cells indicate that CTLH^BP9^ and CTLH^BP10^ have the theoretical ability to regulate all aspects of cellular life (Supplementary Fig. 7A-P; Fig. 8A-D; Supplementary Fig. 9B-E) [52]. The iA549 proteome and ubiquitylome consistently showed a strong association of “RNA metabolism” with both RANBP9 and RANBP10 expression, which is in line with the CPTAC data. Both proteins appeared to be involved in the regulation of the transcriptome in vitro (Supplementary Fig. 7A-P; Fig. 8A-C; Supplementary Fig. 9B-E). This finding is in line with previous evidence indicating that RANBP9 is involved in RNA transcription and splicing [60, 78, 79]. However, we also showed that RANBP10 can also modulate the RNAome and that the regulation exerted by Scorpins could be much more pervasive than previously thought (Supplementary Fig. 7A-P).

The GO term “cell cycle” was also strongly associated with a higher BP9/BP10 ratio in the CPTAC LUAD and LUSQ proteomic data (Supplementary Fig. 7I-P). We used an unbiased protein proliferation signature to corroborate this initial finding (Fig. 7A-F) [53]. Given its elevated expression in NSCLC tumors, RANBP9 is expected to be protumorigenic and proproliferative compared with RANBP10. This assumption is consistent with the fact that RANBP9 enhances signaling through receptor tyrosine kinases such as MET (hepatocyte growth factor receptor), whereas RANBP10 ablates that effect and predominantly expresses RANBP9 in highly proliferative progenitor cells, in contrast with the predominance of RANBP10 in differentiated erythroid cells [9, 35, 76]. Therefore, we can hypothesize that increased expression of the CTLH^BP9^ complex favors the stability of proteins that are associated with cell proliferation in NSCLC. However, since RANBP10 has been reported to promote glioblastoma cell growth and RANBP9 silencing can increase proliferation, we can speculate that these effects are likely cell type dependent.

In iA549 cells, the preferential association of the proliferation-associated protein signature with increased RANBP9 expression was consistent with the increased BP9/BP10 ratio observed in CPTAC NSCLC patients (Fig. 8E-G). However, we found that high levels of RANBP10 caused a significant decrease in the expression of selected proliferation-associated proteins (Fig. 8B-C; Supplementary Fig. 8B-C). Moreover, Doxy-treated iBP10 cells exhibited decreased proliferation, which became significant at 48 h, whereas Doxy-treated iBP9 cells were indistinguishable from their noninduced controls (Fig. 8H).

Interestingly, Doxy-treated iBP9 also showed an increased conversion of glucose to lactate (Fig. 8I) revealing that RANBP9 promotes not only proliferation, but also a carbon metabolism favoring production of biomass via aerobic glycolysis (a.k.a. Warburg effect). These results showed that a decreased BP9/BP10 ratio decelerates cell proliferation (Fig. 8H). We also found that the acute overexpression of RANBP9 or RANBP10 significantly changed the ubiquitylome landscape of A549 cells, which included specific ubiquitylations of proliferation-associated proteins (Fig. 9A-B). Therefore, while the decrease in proliferation observed upon RANBP10 overexpression could be explained by the concomitant decrease in RANBP9, the ubiquitylation and decrease in specific proliferation-associated proteins such as MCM5, MCM7, and SMC1A, for example, cannot be easily explained by the decrease in RANBP9.

**Fig. 11:**
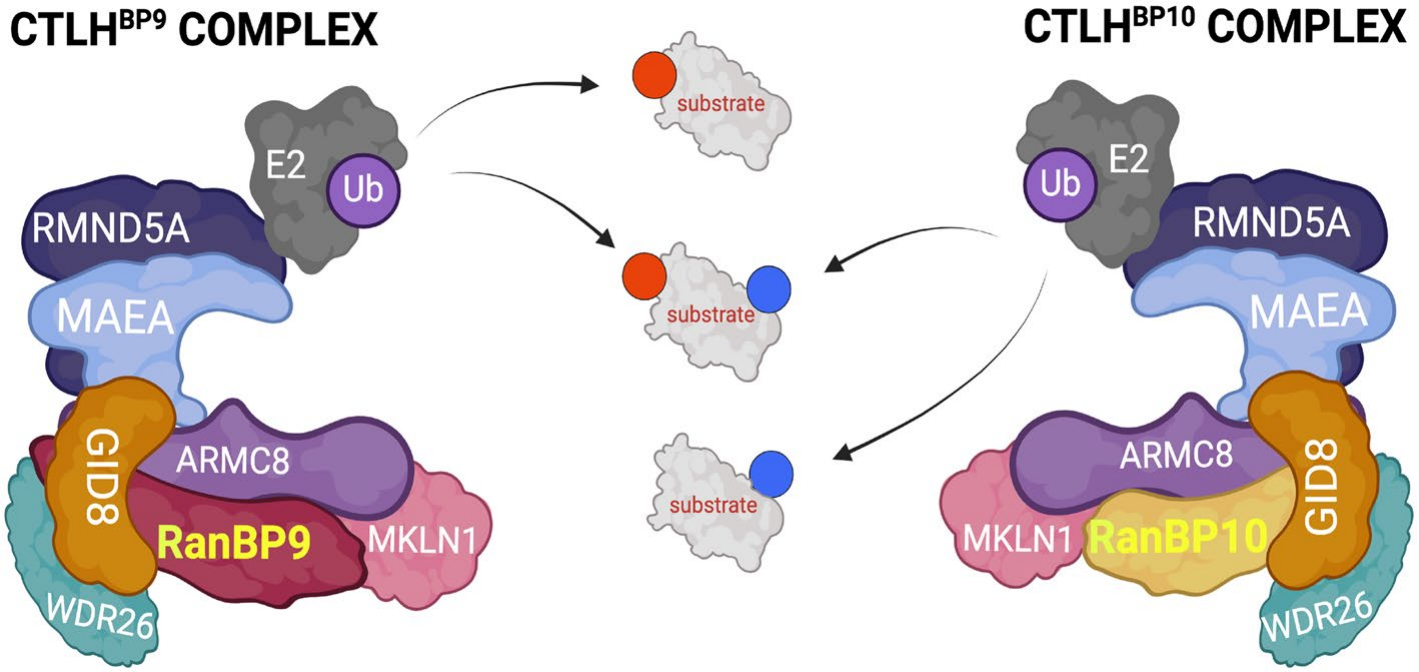
Scorpins cooperate in regulating the CTLH complex ubiquitylation output. Proposed model of the action of the rheostat formed by the two Scorpins. The CTLH^BP9^ and CTLH^BP10^ configurations have both common and selective substrates. The net functional result depends on the molecular role of the specific lysine that is ubiquitylated and/or the cumulative effect of multiple ubiquitylations. The illustration was generated via Biorender (www.biorender.com)

The specific RANBP9-associated lysine ubiquitylations that we observed are largely different from those found to be positively associated with RANBP9 expression in HEK293 cells [55]. In addition, it was found that RANBP9 had a negative impact on glycolysis [55]. However, in that study, RANBP9 was stably silenced, the expression of RANBP10 was not investigated, and differences can be caused by technical and/or cell type-specific factors [55]. Moreover, RANBP9 is generally upregulated in many types of malignancies, which will be in contrast with a negative impact on glycolysis [2]. An in-depth analysis of the observed lysine ubiquitylations suggests a model in which CTLH^BP9^ and CTLH^BP10^ are likely to act on proteins/targets that are CTLH^BP9^ specific, CTLH^BP10^ specific, or common between the two. Several ubiquitylations showed positive or negative associations only with RANBP9 or RANBP10 expression (Fig. 9A-B; Supplementary Material). Interestingly, we found that the ubiquitylation of one or more lysines in some proteins changed when RANBP9 or RANBP10 expression was induced. However, in some cases, the correlation was positive or negative for both Scorpins (concordant), but in other cases, the correlation was opposite (discordant), where the same ubiquitylated lysine was positively correlated with one Scorpin expression but negatively correlated with the other. We also identified cases in which the same protein presented two different ubiquitylated lysine residues, one correlating positively and the other with RANBP9 or RANBP10 negatively (Fig. 9A-C; Supplementary Material). Therefore, we can hypothesize that these proteins whose ubiquitylation changed in more than one residue likely represent proteins on which Scorpins converge to functionally regulate them either in the same or opposite functional direction (Fig. 11). This model can explain how Scorpins have only partially overlapping functions, sometimes having a concordant final effect, and some other times an opposite outcome. This model is also in agreement with previously reported observations showing that the overexpression of RANBP9 increased the stabilization of several binding partners and not their degradation, as expected for an E3 ligase protein. We hypothesize that some proteins undergo degradation upon RANBP9 expression, whereas others are stabilized, and vice versa. A clear example is the proteins that we found to be differentially ubiquitylated on specific lysines that were also previously reported as both putative targets of the CTLH complex and part of the proliferation signature. Our findings and proposed model also explain how the two different configurations of the CTLH complex can have acute effects via immediate ubiquitylation, but also a more stable long-term regulatory impact on the transcriptomic (Fig. 10A-N; Supplementary Fig. 10A-G). This concept is exemplified by the finding that two proliferation associated proteins identified by our study, SF3B3 alongside with XAB2 show a decrease of their protein levels concomitantly with specific ubiquitylations when RanBP10 is acutely overexpressed (Fig. 9D). This last finding underscores the value of performing detailed integration of proteomic data alongside transcriptomic analyses, allowing us to gain insight into functional regulation of post-translational expression patterns, likely through the concerted targeted of key pathway members for proteasomal degradation. Moreover, our findings indicate that RANBP9 and RANBP10 might finely regulate major complexes involved in DNA replication [80–83]. Immunoprecipitation of RANBP10 and RANBP9 confirmed that POLR2B, CACYBP, MCM5 and MCM7 are Scorpin interactors and putative ubiquitylation targets by the CTLH complex (Fig. 9E). Future detailed biochemical studies are needed to validate those ubiquitylations and their functional relevance. However, these observations are supported by previous studies in which proteins involved in DNA replication were reported as putative interactors, including our recent study, which demonstrated that RanBP9 interacts with members of the replisome in normal lungs [36, 55, 60–62].

Our study has several limitations. For example, we cannot conclude that the changes in ubiquitylation observed following altered expression of the CTLH complex are directly caused by changes in the abundance of proteins such as CACYBP, MCM7, and SMC1A. Moreover, we cannot completely exclude the possibility that the effects on protein abundance and ubiquitylation were indirect or that deubiquitylation could be involved [57]. More than one-third of the modified iA549 ubiquitylome that we identified in this study upon Scorpin induction has been previously reported as a putative CTLH complex interactant (Supplementary Material). Therefore, many proteins subject to changes in ubiquitylation are likely not direct targets. In this context, several proteins whose ubiquitylation changes upon RANBP9 or RANBP10 overexpression are E2 or E3 ligases themselves (Supplementary Tables). These findings suggest that the CTLH complex may act indirectly through other ubiquitylation machineries. Evidence supporting this indirect model already exists in yeast, where it was shown that the GID complex can ubiquitylate rsp5 (Reverse Spt-phenotype 5), which is an E3 ligase of the NEDD4 family that in turn regulates vesicular trafficking [84]. If confirmed in mammalian cells, this model would indicate that the CTLH complex has the ability to fine-tune all aspects of cellular life through protein-mediated mechanisms.

## Conclusions

The two Scorpins should be considered as one functional unit that acts as a sophisticated rheostat to modulate the enzymatic output of the CTLH complex. Due to the substantial number of ubiquitylation events that changes when their expression changes, RANBP9 and RANBP10 significantly impact NSCLC pathogenesis, including tumor cell proliferation and metabolism. Since their deletion disrupts the formation of a functional CTLH complex, they should be considered as potential targets for therapy.

## Supplementary Information

Supplementary Material 1. Supplementary Fig. 1. *The combined deletion of Scorpins disrupts the CTLH complex in mouse embryonic fibroblasts*. (A) Mouse embryonic fibroblasts (MEFs) expressing both Scorpins (WT), lacking RanBP9 (9KO), lacking RanBP10 (10KO), or lacking both Scorpins (DKO) were generated from Scorpin WT, BP9 KO, or BP9/BP10 DKO embryos at E12.5 day of gestation. Total cell lysates were analyzed by WB for the presence of the indicated CTLH proteins. The vertical lines on the right side of the panels represent blots from the same gel. Vinculin was used as a loading control for each blot. (B) *GID8, MAEA, and MKLN1 protein changes are not caused by changes in their corresponding transcript levels in A549 cells*. RNA was extracted from the A549 cell line shown in Fig. 1A. Quantitative RT‒PCR was performed in triplicate to measure the transcript levels of CTLH proteins, as illustrated in alphabetical order. (C) *GID8, MAEA, and MKLN1 protein changes are not caused by changes in their corresponding transcript levels in H460 cells*. RNA was extracted from the H460 cell line shown in Fig. 1B. RT‒PCR was performed to measure the transcript levels of CTLH proteins, as illustrated in alphabetical order. The statistical significance of differences between N and T was assessed by two-way ANOVA using GraphPad Prism. * *p* = 0.05; ** *p* = 0.01; *** *p* = 0.001; **** *p* = 0.0001.

Supplementary Material 2. Supplementary Fig. 2. (A) *Schematic representation of the safe-harbor strategy used to engineer Scorpin-inducible A549 cells*. RANBP9 or RANBP10 cDNA was subsequently cloned and inserted into a vector for “safe harbor” site-specific insertion into intron one of the PPP1R2C gene (AAVS1) locus (top panel). The vector contains 2 homology arms (HA 5’, HA 3’, blue), a strong Splicing-Acceptor sequence (SA, red), a promoter-less puromycin resistance gene (PuroR, cyan) followed by polyA sequences (pA), a third-generation tetracycline-responsive element (TRE3G) cassette for the binding of the Tet regulator, a Tet-On™ 3G doxycycline-responsive transcription factor (encoding the rTTA 3G protein, yellow) driven by the CMV early enhancer element, the chicken b-Actin promoter, and the rabbit b-Globin splice acceptor (CAG). After site-specific recombination, the construct is expressed under the control of the PPP1R12C gene. Doxycycline treatment triggers the transcriptional activity of rTTA and leads to RANBP9 or RANBP10 expression (bottom panel). (B) *RANBP9 or RANBP10 re-expression is sufficient to stabilize GID8 and MAEA and restore CTLH complex formation*. *Quantitation of the CTLH protein band intensity in the Scorpin DKO A549 cells is shown in *Fig. 2. ImageJ version 1.53t (https://imagej.nih.gov/ij/) was used to quantify the intensity of the bands presented in the panels in Fig. 2. The absolute values were normalized to the intensity of the vinculin band in each blot. The statistical significance of differences between N and T was assessed by two-way ANOVA using GraphPad Prism. * *p* = 0.05; ** *p* = 0.01; n.s. = not statistically significant.

Supplementary Material 3. Supplementary Fig. 3. (A) *RANBP9 and RANBP10 cross-regulate each other’s expression*. Total cell lysates from Scorpin WT A549, A549 iBP9, and A549 iBP10 cells together with Scorpin DKO A549 cells (used as a negative control) were probed by WB for the expression of the indicated CTLH proteins before and after exposure to Doxy at 1 mg/mL for 24 h. The vertical lines on the right side of the panels represent blots from the same gel. Vinculin was used as a loading control for each blot. ImageJ version 1.53t (https://imagej.nih.gov/ij/) was used for the quantitation of the RANBP9 and RANBP10 band intensities. (B) *Quantification of the CTLH protein band intensity in A549 WT cells shown in Supplementary Fig. 3A*. ImageJ version 1.53t (https://imagej.nih.gov/ij/) was used to quantify the intensity of the bands shown in Fig. 3A. The absolute values were normalized to the intensity of the vinculin band in each blot. The statistical significance of the differences was assessed by two-way ANOVA using GraphPad Prism. * *p* = 0.05; ** *p* = 0.01. (C) *Measurement of CTLH complex transcript levels in Scorpin WT A549 inducible cells shown in Supplementary Fig. 3A*. RNA was extracted from the A549 cell line shown in Supplementary Fig. 3A. RT‒PCR was performed to measure the transcript levels of CTLH proteins as reported in alphabetical order. The statistical significance of the differences was assessed via two-way ANOVA using GraphPad PRISM. * *p* = 0.05; ** *p* = 0.01; *** *p* = 0.001; **** *p* = 0.0001.

Supplementary Material 4. Supplementary Fig. 4. *RANBP9 and GID8 protein expression is significantly upregulated in NSCLC T* vs* N*. (A) Seventeen (17) blots were generated with protein extracts from 50 NSCLC frozen tumors with matched normal adjacent tissues. For each blot, 3 different pairs of tumors (T) and matched controls (N) were probed for the presence of RANBP9, GID8, and vinculin (loading control). Blot number 13 shows only two tumors and matched controls. In lanes one and two, Scorpin WT A549 and A549 BP9 KO (blots 1 to 11) or Scorpin WT and BP9 KO H460 (blots 12 to 17) were used as positive and negative controls, respectively. ImageJ version 1.53t (https://imagej.nih.gov/ij/) was used to quantify the intensity of the bands. The statistical significance of the differences between N and T was assessed via two-way ANOVA. **** = *p* ≤ 0.0001 via RStudio. (B) *GID8 and RANBP9 transcripts are upregulated, whereas RanBP10 mRNA is downregulated in the TCGA NSCLC cohort compared with the matched normal cohort*. Quantitation of mRNA expression in LUAD (B) and LUSQ (C) samples from the TCGA database revealed that both the RANBP9 and GID8 mRNAs are significantly overexpressed, whereas the RANBP10 transcript is significantly downregulated. Data from https://gdac.broadinstitute.org/. **** *p* < 0.0001; ** *p* < 0.01; n.s. = not statistically significant.

Supplementary Material 5. Supplementary Fig. 5. *RANBP9 and GID8 are overexpressed, whereas RanBP10 is downregulated in the CPTAC collection of both LUAD and LUSQ tumors compared with matched normal adjacent tissue*. The analysis of the LUAD and LUSQ CPTAC data shows that both the RANBP9 (A, D) and RANBP10 (B, E) proteins significantly correlate with their binding partner GID8. The correlation between the Scorpins and GID8 becomes particularly significant when the maximal expression of either one of the two (RANBPMAX) is used (C, F), illustrating the stoichiometric relationship of the complex. The dots represent single tumors, and the color indicates the level of expression per the color scale shown. When points are below and to the right of the trendline, this indicates high GID8 expression with low RANBP9 expression, which has ‘red’ expression of RANBP10, and vice versa for RANBP10 vs RANBP9. *P* value from linear regression performed in RStudio. The analysis of the LUAD and LUSQ CPTAC data revealed that both *RANBP9* (G, J) and *GID8* mRNAs (H, K) are upregulated, whereas the *RANBP10* transcript (I, L) is downregulated. The data were downloaded from https://kb.linkedomics.org/. The statistical analysis was performed via RStudio. **** *p* < 0.001; *** *p* = 0.001.

Supplementary Material 6. Supplementary Fig. 6. *RANBP9 and RANBP10 expression was positively correlated with GID8 expression in both the LUAD and LUSQ CPTAC datasets*. Volcano plots illustrating the linear regression analysis of proteins related to GID8 in the LUAD (A) and LUSQ (B) CPTAC data collections. Protein expression was converted to a Z score, and regression analysis was performed in RStudio. Unadjusted log10-transformed *p* values on the y-axis are plotted against regression estimates. Values exceeding the plotted ranges are shown at the corresponding maximum or minimum values. The CTLH family members RANBP9, RANBP10, GID8, MAEA, and WRD26 are labeled.

Supplementary Material 7. Supplementary Fig. 7. *RANBP9 expression is correlated with cell proliferation according to the CPTAC NSCLC data*. The results of the top five gene set enrichment analyses (GSEAs) positively associated with RANBP9 in the CPTAC LUAD (A) and LUSQ (B), positively associated with RANBP10 in the CPTAC LUAD (C) and LUSQ (D), negatively associated with RANBP9 in the CPTAC LUAD (E) and LUSQ (F), and negatively associated with RANBP10 in the CPTAC LUAD (G) and LUSQ (H) datasets were downloaded from the WEB-basedGEne SeT AnaLysis Toolkit (WebGestalt: webgestalt.org). The GSEA results are listed from the top left to the bottom right in descending order of the normalized enrichment score (NES). The LUAD and LUSQ data were used to perform a Metascape analysis for enrichment of Gene Ontology (GO) terms (https://metascape.org; Zhou et al. Nature Comm., 2019) ^52^. Analysis of proteins positively correlated with RANBP9 expression in LUAD (I) and LUSQ (J), with RANBP10 expression in LUAD (K) and LUSQ (L), or negatively correlated with RANBP9 expression in LUAD (M) and LUSQ (N), and negatively correlated with RANBP10 expression in LUAD (O) and LUSQ (P).
*Compared with RANBP9, the overexpression of RANBP10 causes different changes in the NSCLC proteome, downregulating several proliferation-associated proteins.* (A) *Experimental outline of the *in vitro* experiment used to study the proteome upon Scorpin induction*. Scorpin WT iBP9 and iBP10 cell lines were exposed to 1 mg/mL Doxy for 24 h. Quadruplicates of total cell lysates were harvested and processed for analysis via mass spectrometry. (B) Heatmaps showing RANBP9-selective or (C) RANBP10-selective proteins that are differentially expressed after Doxy treatment, corresponding to Fig. 8D. Proteins were considered to be positively associated with RANBP9 or RANBP10 if they were significantly different between the Doxy-treated and control conditions and significantly different in expression vs the opposite Scorpin member in the Doxy-treated condition, each with *P* < 0.05 according to Student’s t test.

Supplementary Material 8. Supplementary Fig. 8. *Compared with RANBP9, the overexpression of RANBP10 causes different changes in the NSCLC ubiquitylome, which includes proliferation-associated proteins.* (A) *Experimental outline of the *in vitro* experiment used to study the ubiquitylome upon Scorpin induction*. Scorpin WT iBP9 and iBP10 cell lines were exposed to 1 mg/mL doxycycline for 24 h and to the proteasome inhibitor MG132 (10 mg/mL) for the last 4 h. Quadruplicates of total cell lysates were harvested and processed for analysis via mass spectrometry. B-E) Metascape analysis of ubiquitylated proteins in iA549 cells associated positively with RANBP9 (B), negatively with RANBP9 (C), positively with RANBP10 (D), and negatively with RANBP10 (E). The analysis was performed at https://metascape.org (Zhou et al. Nature Comm., 2019) ^52^. (F) Bar graph reporting the number of proliferation-associated proteins (PAPs ^53^) found in total (gray bar), in the list of proteins positively associated with RANBP10 (blue bar), and in the list of proteins positively associated with RANBP9 (red bar), setting the statistical threshold at *p* = 0.1. This latter group was further analyzed for the presence of specific PPA ubiquitylations that were increased in positive association with RANBP9 (RANBP9UbiqUp), in negative association with RANBP10 (RANBP10UbiquDown) in negative association with RANBP9 (RANBP9UbiquDown) and in positive association with RANBP10 (RANBP10UbiquUp). Fisher’s test was used to assess the statistical significance of the findings. OR = odds ratio. G) Proliferation associated proteins coimmunoprecipitate with RANBP10. V5-tagged endogenous RANBP9 expression was induced using Doxy at 10 ng/mL. After 24 h 1 mg of total cell lysate was immunoprecipitated by primary antibodies to pull-down POLR2B, PABPC1, CACYBP, MCM5, and MCM7. Rabbit IgG pull-down was used as negative control, and GID8 pull-down was used as positive control. The presence of RANBP9 was detected by HRP-conjugated anti-V5. GAPDH is used as loading control.

Supplementary Material 9. Supplementary Fig. 9. *RANBP9 regulates key oncogenic pathways preferentially at protein or at transcript level*. Analysis of individual mRNAs vs. proteins from CPTAC LUAD (A) and LUSQ (B) showing the linear regression vs. RANBP10 protein. The *p*-value of two orders of magnitude established arbitrarily using the numeric difference defines genes having a stronger mRNA (red) or protein (blue) association with RANBP10. Pathway analysis in LUAD (C) and LUSQ (D) shows that certain pathways have a stronger RANBP10 association with proteomic (blue) rather than transcriptomic (red) data while other have the opposite trend. The comparison of proteomic of LUAD vs LUSQ (E) and transcriptomic (F) analyses fails to show concordance between the two different CPTAC lung cancer collections. G) Key pathways showing the RANBP9 top 5 predominantly mRNA associations and top 5 predominantly proteomic associations. The RANBP9 proteomic associations focus strongly on RNA metabolism including the spliceosome.

Supplementary Material 10.

Supplementary Material 11.

Supplementary Material 12.

Supplementary Material 13.

Supplementary Material 14.

Supplementary Material 15.

Supplementary Material 16.

Supplementary Material 17.

Supplementary Material 18.

## Data Availability

“Data is provided within the manuscript or supplementary information files”. Data is also available upon request.

## Abbreviations

NSCLC: Non-Small Cell Lung Cancer
RANBP9: Ran Binding Protein 9
RANBP10: Ran Binding Protein 10
GID8: Glucose-induced degradation Deficient 8
CTLH: C-Terminal to LisH
CTLH^BP9^: CTLH complex built on RANBP9
CTLH^BP10^: CTLH complex built on RANBP10
SCORPIN: Spry-COntaing Ran binding ProteIN
ARMC8: Armadillo Repeat Containing protein 8
GID4: Glucose-Induced degradation Deficient 4
MAEA: Macrophage Erythroblast Attacher E3 ligase
MKLN1: Muskelin 1
RMND5A: Required in Meiotic Division 5 A
RMND5B: Required in Meiotic Division 5 B
WDR26: WD Repeat containing protein 26
YPEL5: Yippee Like protein 5
KO: Knockout
DKO: Double KO
MEFs: Mouse Embryonic Fibroblasts
Doxy: Doxycycline
LUAD: Lung Adenocarcinoma
LUSQ: Lung Squamous carcinoma
T: Tumor tissue
N: Normal adjacent tissue
WB: Western blot
Co-IP: Co-immunoprecipitation
CPTAC: Clinical Proteomic Tumor Analysis Consortium
PAPs: Proliferation-Associated Proteins
GSEA: Gene Set Enrichment Analysis
GO: Gene ontology
iA549 DKO: Scorpin DKO A549 Doxy-inducible controls
A549 DKO iBP9: Scorpin DKO A549 with Doxy-inducible RANBP9
A549 DKO iBP10: Scorpin DKO A549 with Doxy-inducible RANBP10
iA549 WT: Parental A549 Doxy-inducible controls
A549 WT iBP9: Parental A549 with Doxy-inducible RANBP9
A549 WT iBP10: Parental A549 with Doxy-inducible RANBP10
