# Supplementary material for: RANBP9 and RANBP10 cooperate in regulating non-small cell lung cancer proliferation
Source: J Exp Clin Cancer Res. 2025 Aug 29;44:259. doi: 10.1186/s13046-025-03491-8 (PMC12395873; doi:10.1186/s13046-025-03491-8)
Supplement: Supplementary file 1 — Supplementary Material 1. Supplementary Fig. 1. The combined deletion of Scorpins disrupts the CTLH complex in mouse embryonic fibroblasts. (A) Mouse embryonic fibroblasts (MEFs) expressing both Scorpins (WT), lacking RanBP9 (9KO), lacking RanBP10 (10KO), or lacking both Scorpins (DKO) were generated from Scorpin WT, BP9 KO, or BP9/BP10 DKO embryos at E12.5 day of gestation. Total cell lysates were analyzed by WB for the presence of the indicated CTLH proteins. The vertical lines on the right side of the panels represent blots from the same gel. Vinculin was used as a loading control for each blot. (B) GID8, MAEA, and MKLN1 protein changes are not caused by changes in their corresponding transcript levels in A549 cells. RNA was extracted from the A549 cell line shown in Fig. 1A. Quantitative RT‒PCR was performed in triplicate to measure the transcript levels of CTLH proteins, as illustrated in alphabetical order. (C) GID8, MAEA, and MKLN1 protein changes are not caused by changes in their corresponding transcript levels in H460 cells. RNA was extracted from the H460 cell line shown in Fig. 1B. RT‒PCR was performed to measure the transcript levels of CTLH proteins, as illustrated in alphabetical order. The statistical significance of differences between N and T was assessed by two-way ANOVA using GraphPad Prism. * p = 0.05; ** p = 0.01; *** p = 0.001; **** p = 0.0001. [file 13046_2025_3491_MOESM1_ESM.pdf]

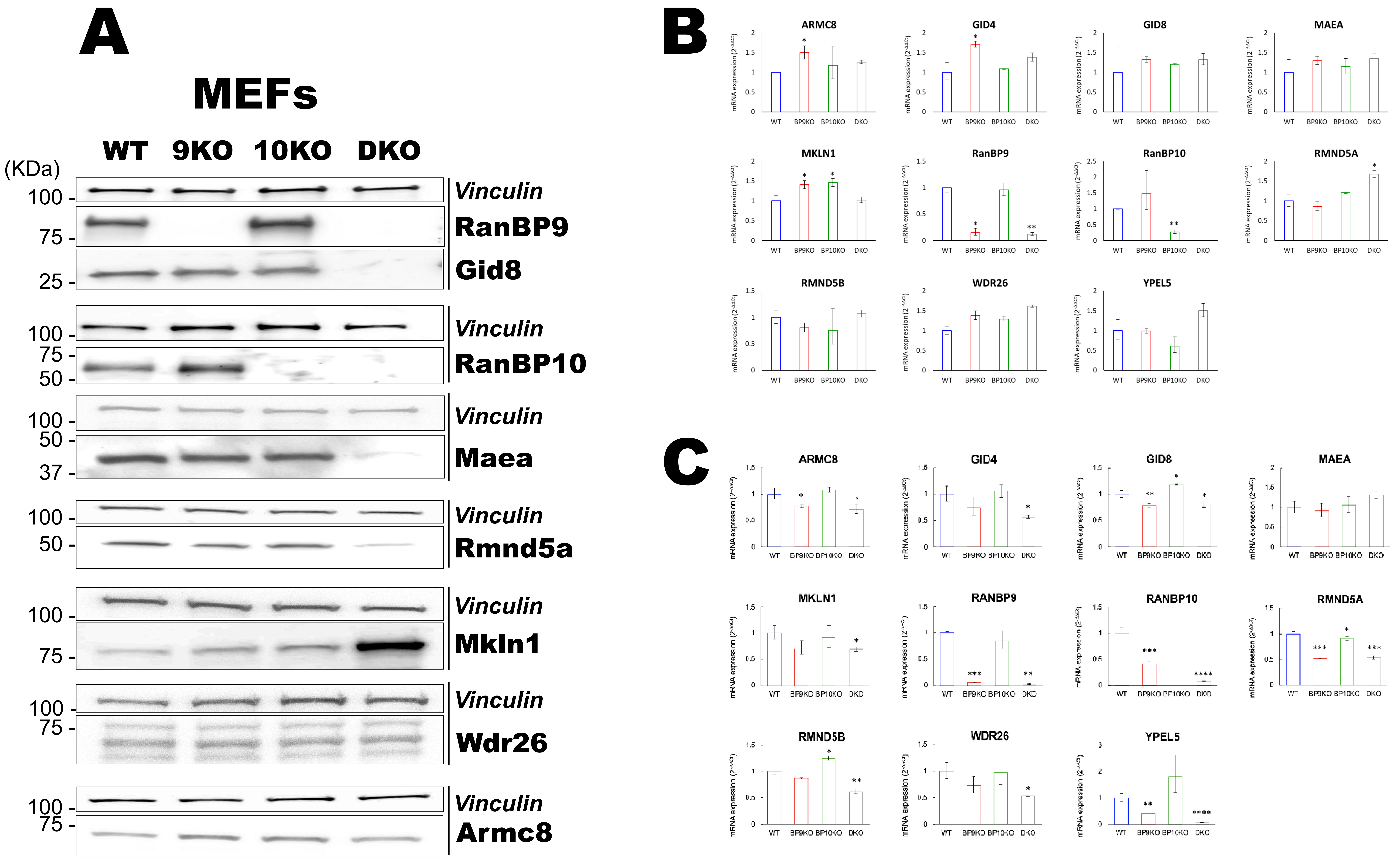
A
MEFs
WT 9KO 10KO DKO
(KDa)
100
75
25
100
75
50
100
50
37
100
50
100
75
100
75
100
75
Vinculin
RanBP9
Gid8
RanBP10
Maea
Rmnd5a
Mkln1
Wdr26
Armc8
B
ARMC8
GID4
GID8
MAEA
MKLN1
RanBP9
RanBP10
RMND5A
RMND5B
WDR26
YPEL5
mRNA expression (2^-ΔΔCt)
WT BP9KO BP10KO DKO
C
ARMC8
GID4
GID8
MAEA
MKLN1
RANBP9
RANBP10
RMND5A
RMND5B
WDR26
YPEL5
WT BP9KO BP10KO DKO
