# Supplementary figures and images for: RANBP9 and RANBP10 cooperate in regulating non-small cell lung cancer proliferation
Source: J Exp Clin Cancer Res. 2025 Aug 29;44:259. doi: 10.1186/s13046-025-03491-8 (PMC12395873; doi:10.1186/s13046-025-03491-8)

A

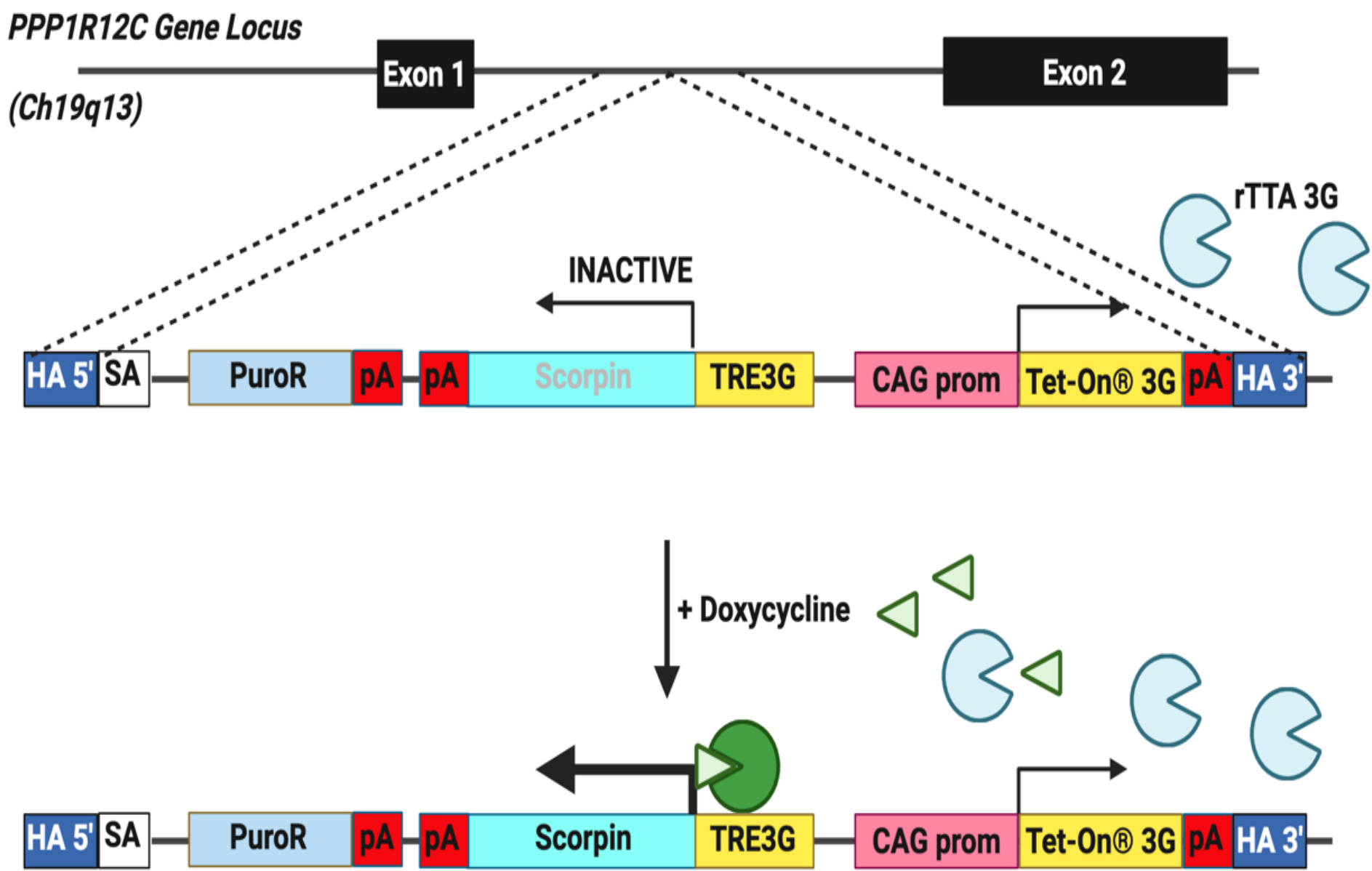

B

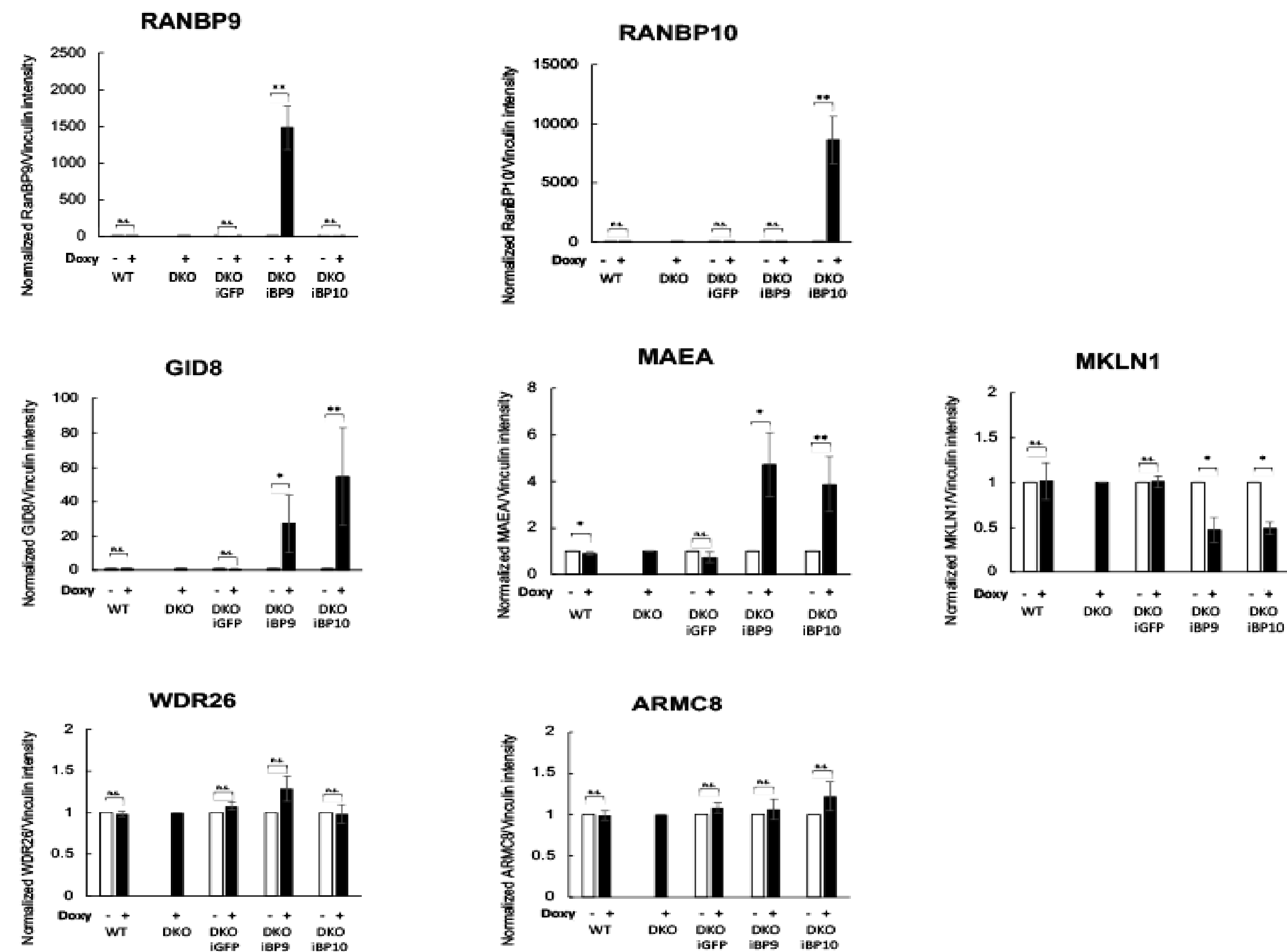

Supplement: Supplementary file 2 — Supplementary Material 2. Supplementary Fig. 2. (A) Schematic representation of the safe-harbor strategy used to engineer Scorpin-inducible A549 cells. RANBP9 or RANBP10 cDNA was subsequently cloned and inserted into a vector for “safe harbor” site-specific insertion into intron one of the PPP1R2C gene (AAVS1) locus (top panel). The vector contains 2 homology arms (HA 5’, HA 3’, blue), a strong Splicing-Acceptor sequence (SA, red), a promoter-less puromycin resistance gene (PuroR, cyan) followed by polyA sequences (pA), a third-generation tetracycline-responsive element (TRE3G) cassette for the binding of the Tet regulator, a Tet-On™ 3G doxycycline-responsive transcription factor (encoding the rTTA 3G protein, yellow) driven by the CMV early enhancer element, the chicken b-Actin promoter, and the rabbit b-Globin splice acceptor (CAG). After site-specific recombination, the construct is expressed under the control of the PPP1R12C gene. Doxycycline treatment triggers the transcriptional activity of rTTA and leads to RANBP9 or RANBP10 expression (bottom panel). (B) RANBP9 or RANBP10 re-expression is sufficient to stabilize GID8 and MAEA and restore CTLH complex formation. Quantitation of the CTLH protein band intensity in the Scorpin DKO A549 cells is shown in Fig. 2. ImageJ version 1.53t (https://imagej.nih.gov/ij/) was used to quantify the intensity of the bands presented in the panels in Fig. 2. The absolute values were normalized to the intensity of the vinculin band in each blot. The statistical significance of differences between N and T was assessed by two-way ANOVA using GraphPad Prism. * p = 0.05; ** p = 0.01; n.s. = not statistically significant. [file 13046_2025_3491_MOESM2_ESM.pdf]

A

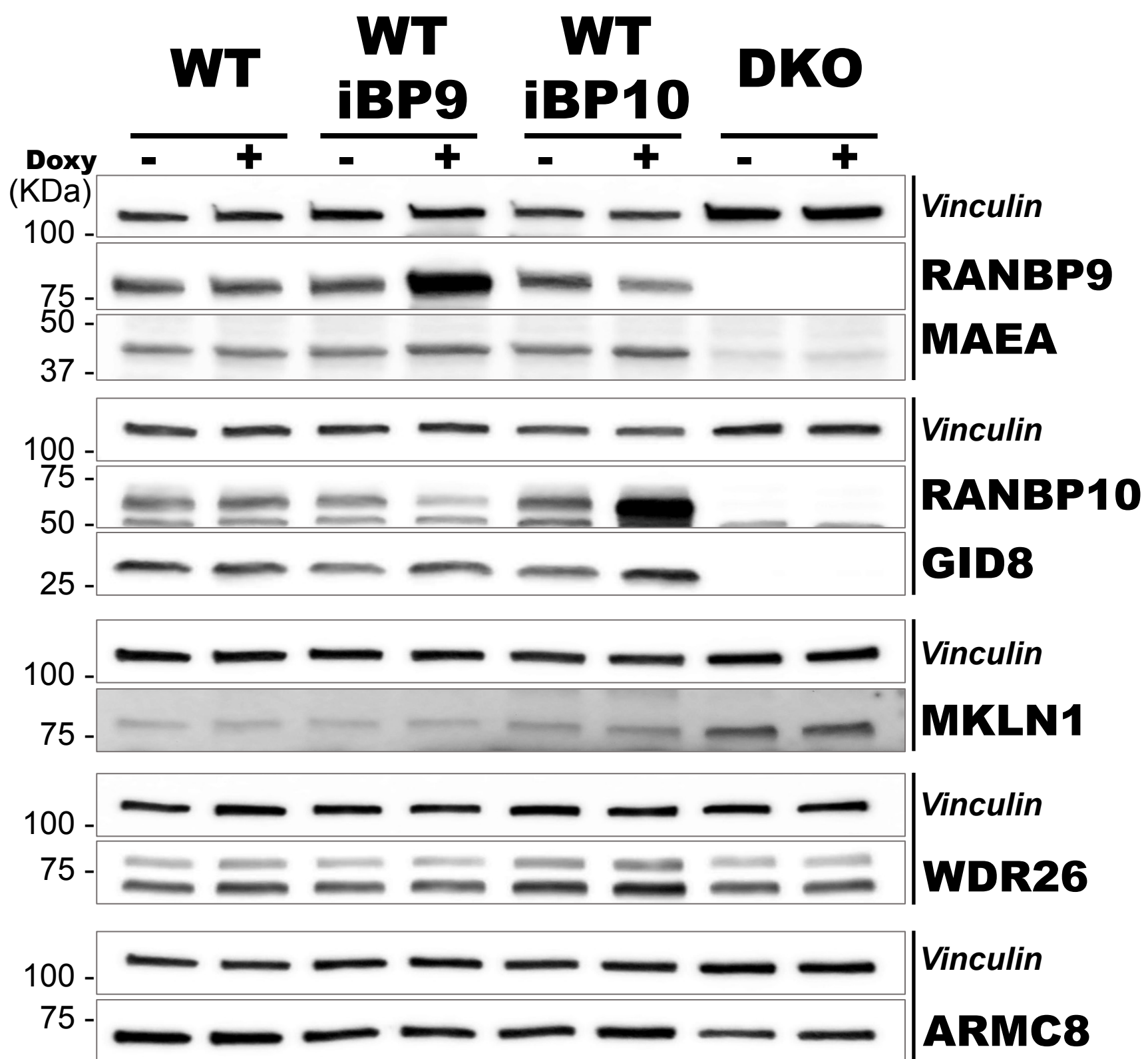

B

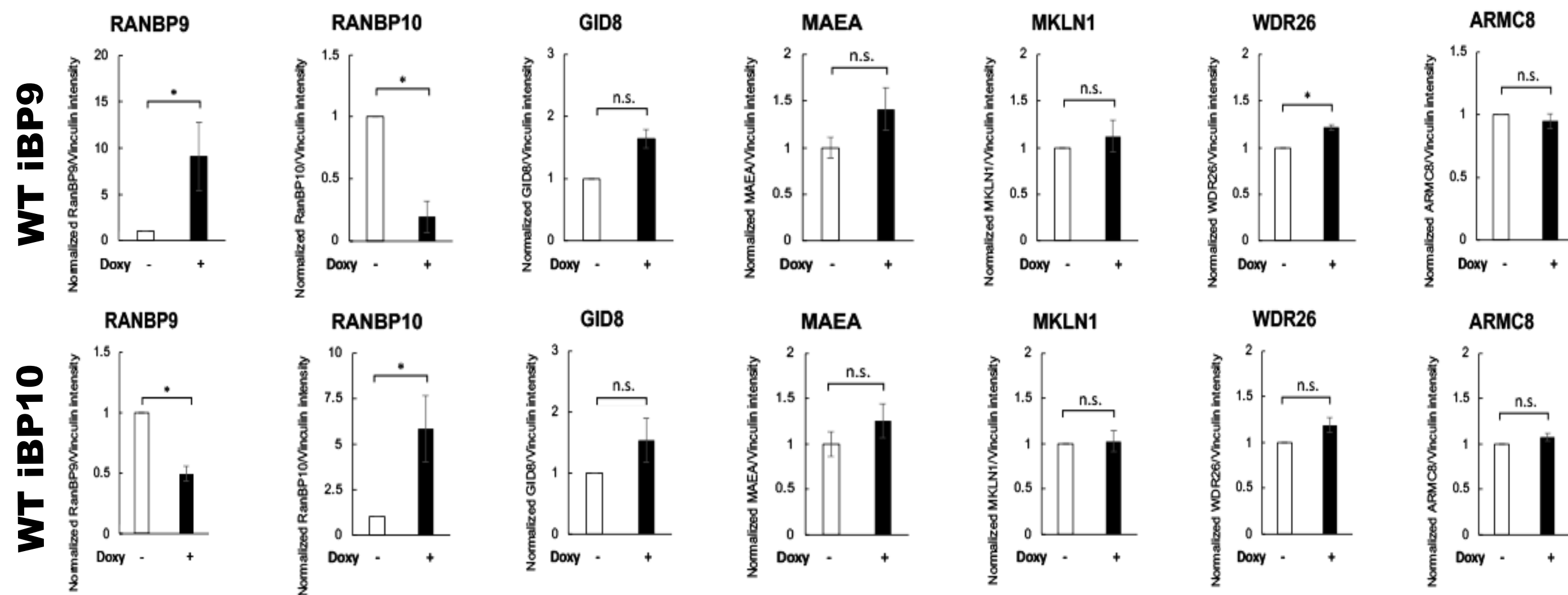

C

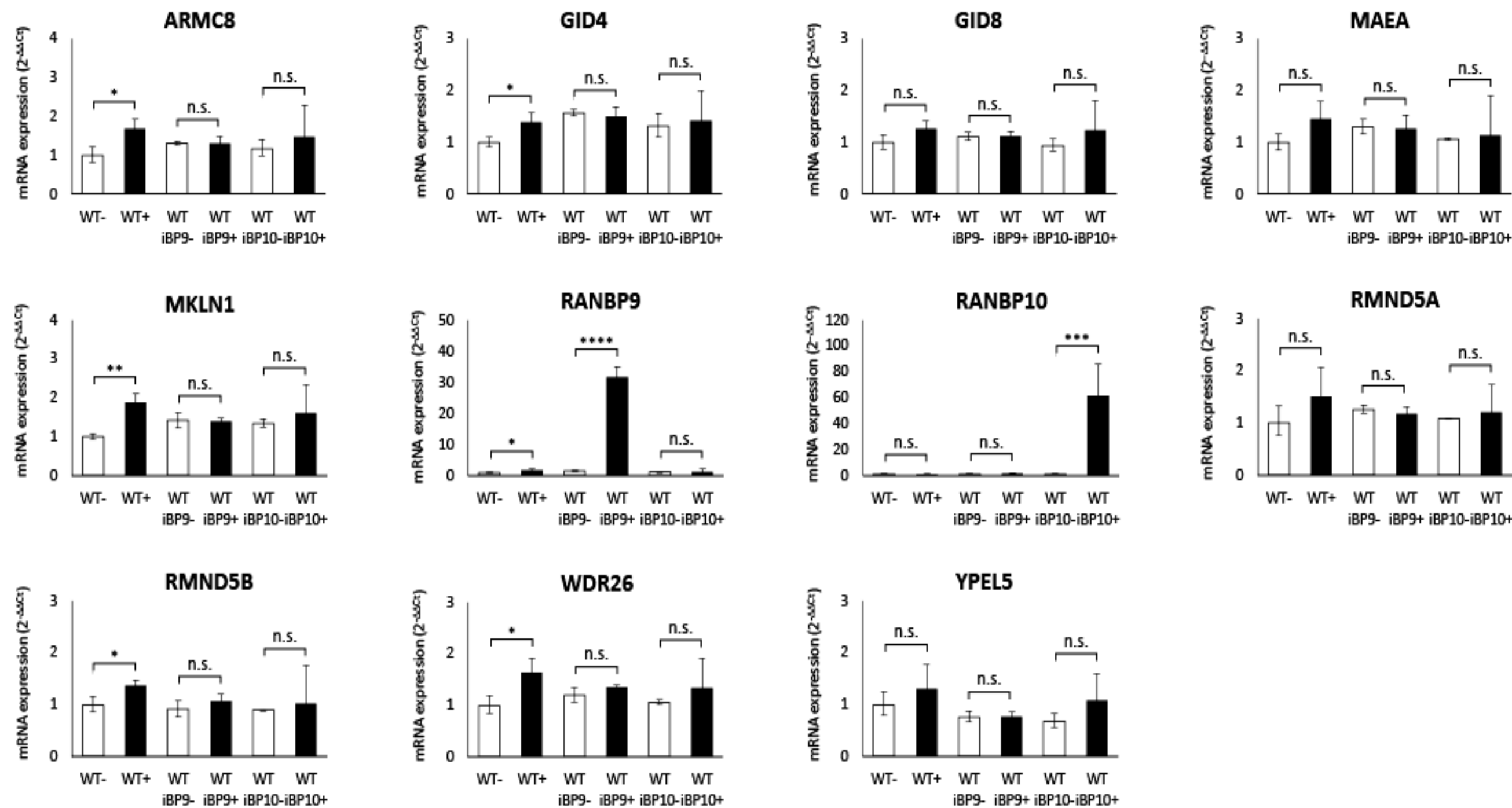

Supplement: Supplementary file 3 — Supplementary Material 3. Supplementary Fig. 3. (A) RANBP9 and RANBP10 cross-regulate each other’s expression. Total cell lysates from Scorpin WT A549, A549 iBP9, and A549 iBP10 cells together with Scorpin DKO A549 cells (used as a negative control) were probed by WB for the expression of the indicated CTLH proteins before and after exposure to Doxy at 1 mg/mL for 24 h. The vertical lines on the right side of the panels represent blots from the same gel. Vinculin was used as a loading control for each blot. ImageJ version 1.53t (https://imagej.nih.gov/ij/) was used for the quantitation of the RANBP9 and RANBP10 band intensities. (B) Quantification of the CTLH protein band intensity in A549 WT cells shown in Supplementary Fig. 3A. ImageJ version 1.53t (https://imagej.nih.gov/ij/) was used to quantify the intensity of the bands shown in Fig. 3A. The absolute values were normalized to the intensity of the vinculin band in each blot. The statistical significance of the differences was assessed by two-way ANOVA using GraphPad Prism. * p = 0.05; ** p = 0.01. (C) Measurement of CTLH complex transcript levels in Scorpin WT A549 inducible cells shown in Supplementary Fig. 3A. RNA was extracted from the A549 cell line shown in Supplementary Fig. 3A. RT‒PCR was performed to measure the transcript levels of CTLH proteins as reported in alphabetical order. The statistical significance of the differences was assessed via two-way ANOVA using GraphPad PRISM. * p = 0.05; ** p = 0.01; *** p = 0.001; **** p = 0.0001. [file 13046_2025_3491_MOESM3_ESM.pdf]

A

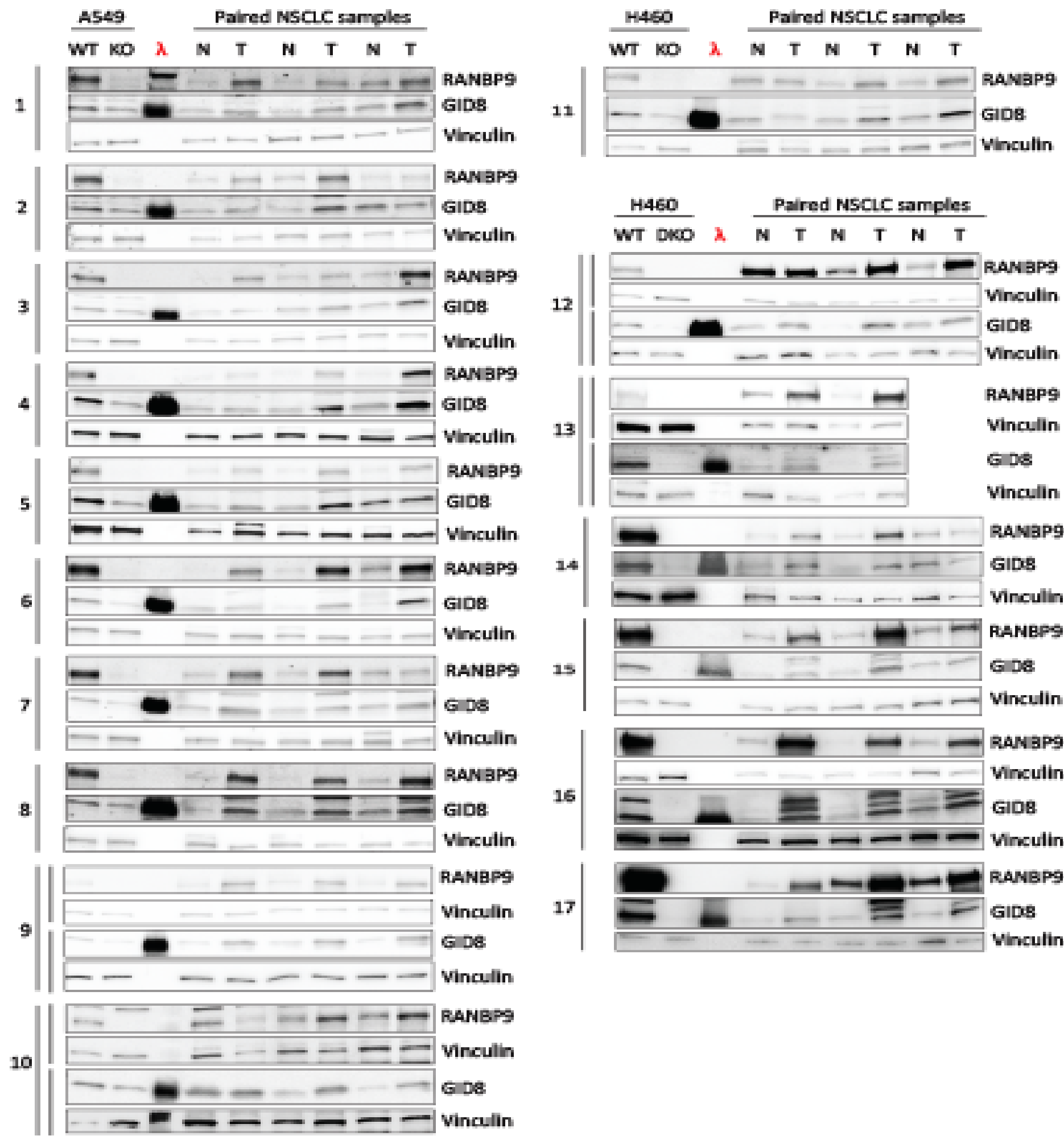

B

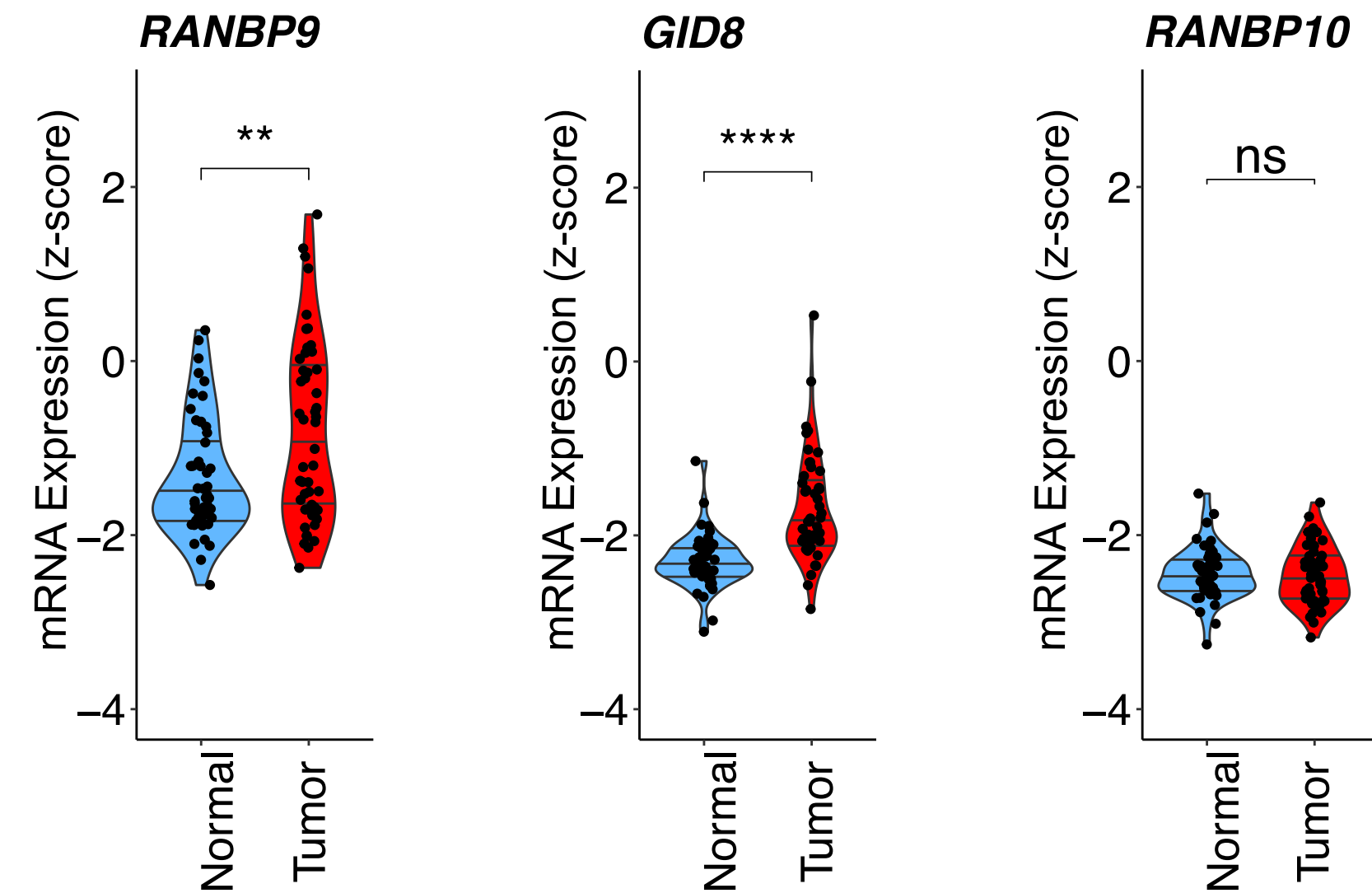

C

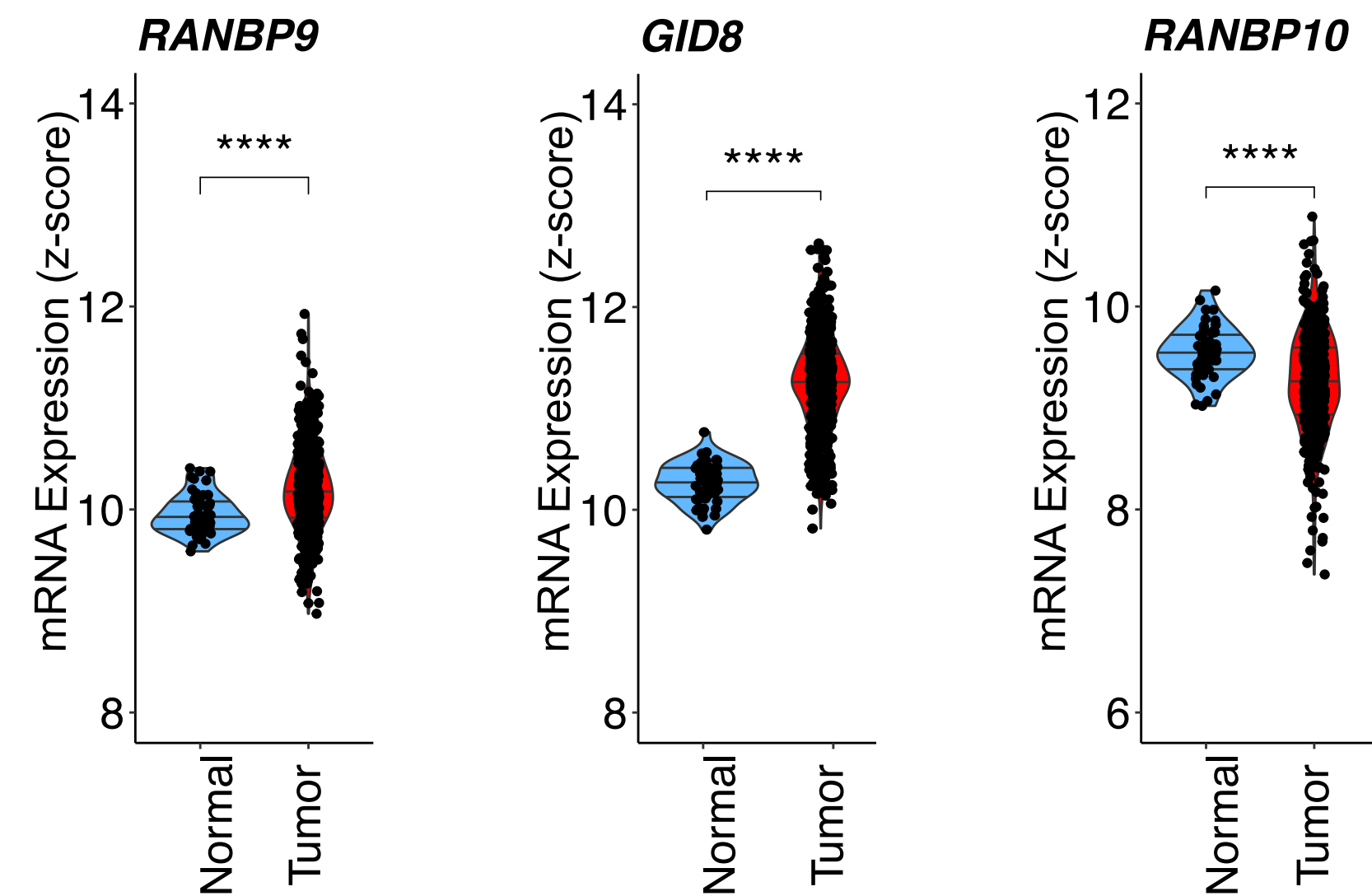

Supplement: Supplementary file 4 — Supplementary Material 4. Supplementary Fig. 4. RANBP9 and GID8 protein expression is significantly upregulated in NSCLC T vs N. (A) Seventeen (17) blots were generated with protein extracts from 50 NSCLC frozen tumors with matched normal adjacent tissues. For each blot, 3 different pairs of tumors (T) and matched controls (N) were probed for the presence of RANBP9, GID8, and vinculin (loading control). Blot number 13 shows only two tumors and matched controls. In lanes one and two, Scorpin WT A549 and A549 BP9 KO (blots 1 to 11) or Scorpin WT and BP9 KO H460 (blots 12 to 17) were used as positive and negative controls, respectively. ImageJ version 1.53t (https://imagej.nih.gov/ij/) was used to quantify the intensity of the bands. The statistical significance of the differences between N and T was assessed via two-way ANOVA. **** = p ≤ 0.0001 via RStudio. (B) GID8 and RANBP9 transcripts are upregulated, whereas RanBP10 mRNA is downregulated in the TCGA NSCLC cohort compared with the matched normal cohort. Quantitation of mRNA expression in LUAD (B) and LUSQ (C) samples from the TCGA database revealed that both the RANBP9 and GID8 mRNAs are significantly overexpressed, whereas the RANBP10 transcript is significantly downregulated. Data from https://gdac.broadinstitute.org/. **** p < 0.0001; ** p < 0.01; n.s. = not statistically significant. [file 13046_2025_3491_MOESM4_ESM.pdf]

# LUAD

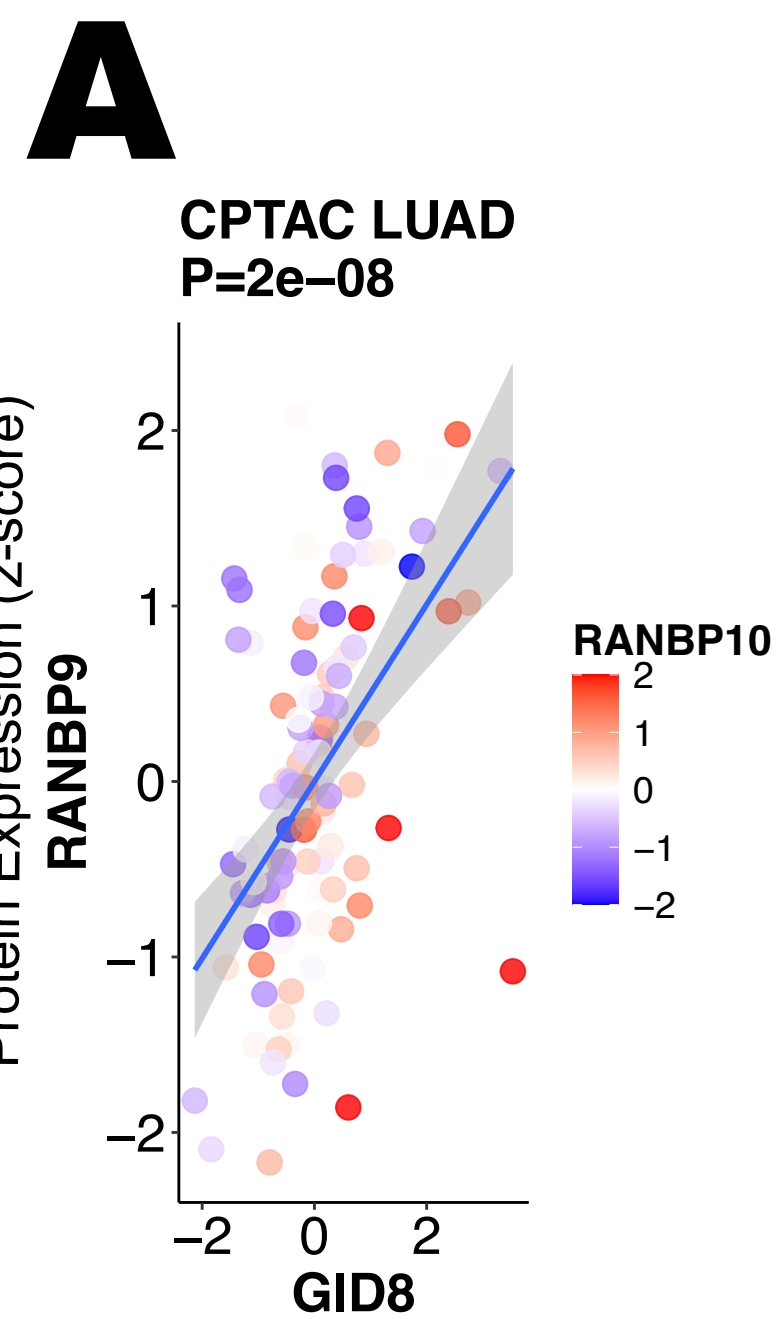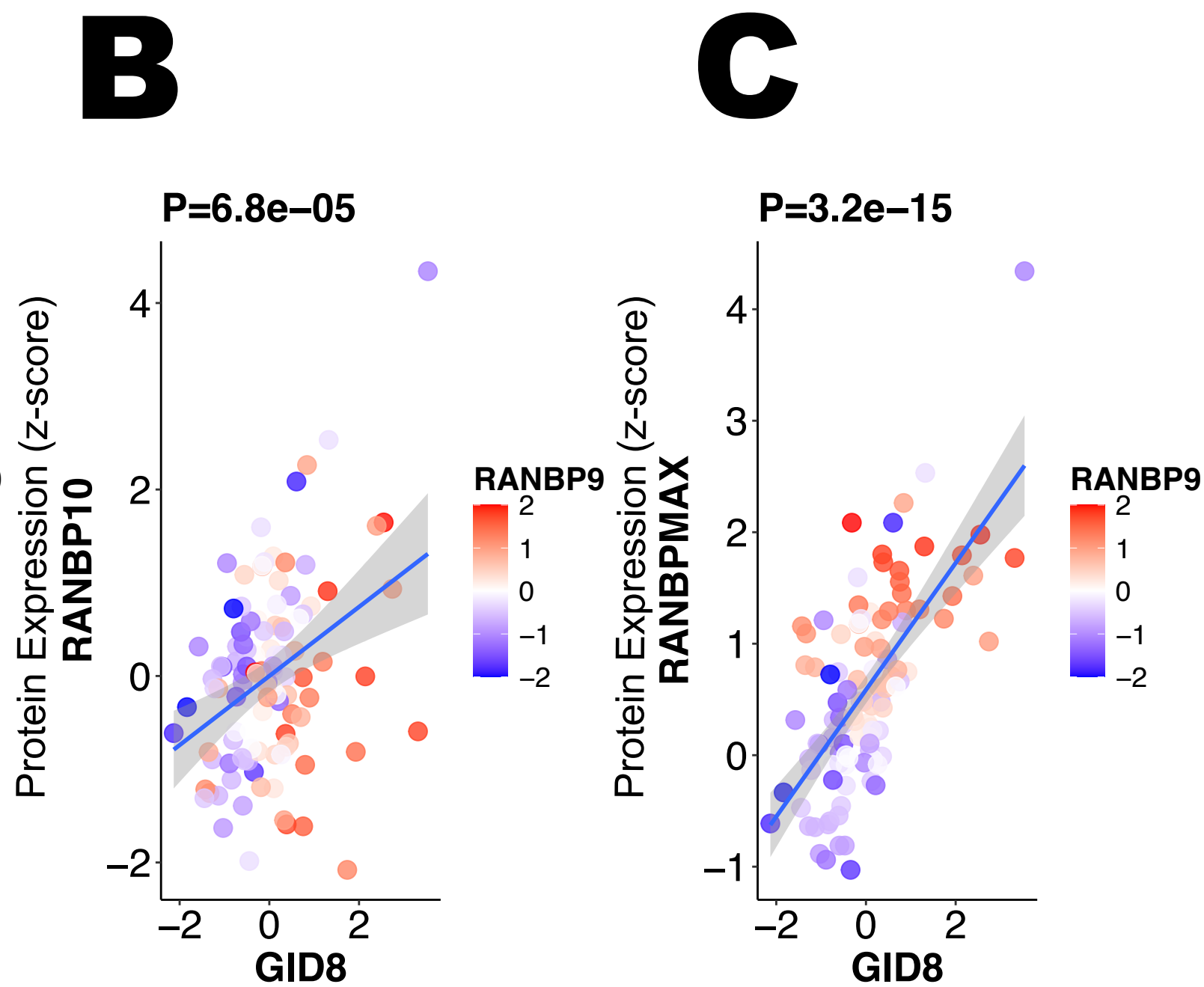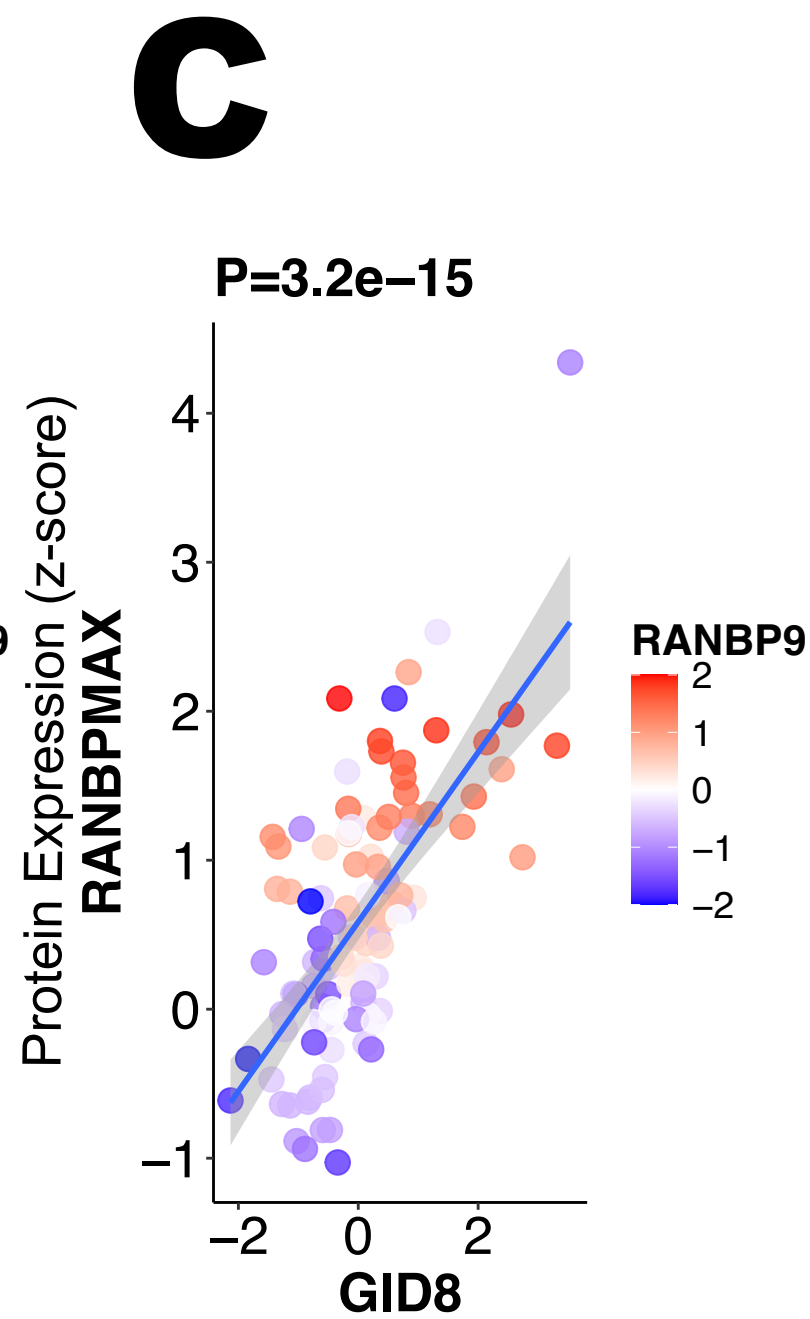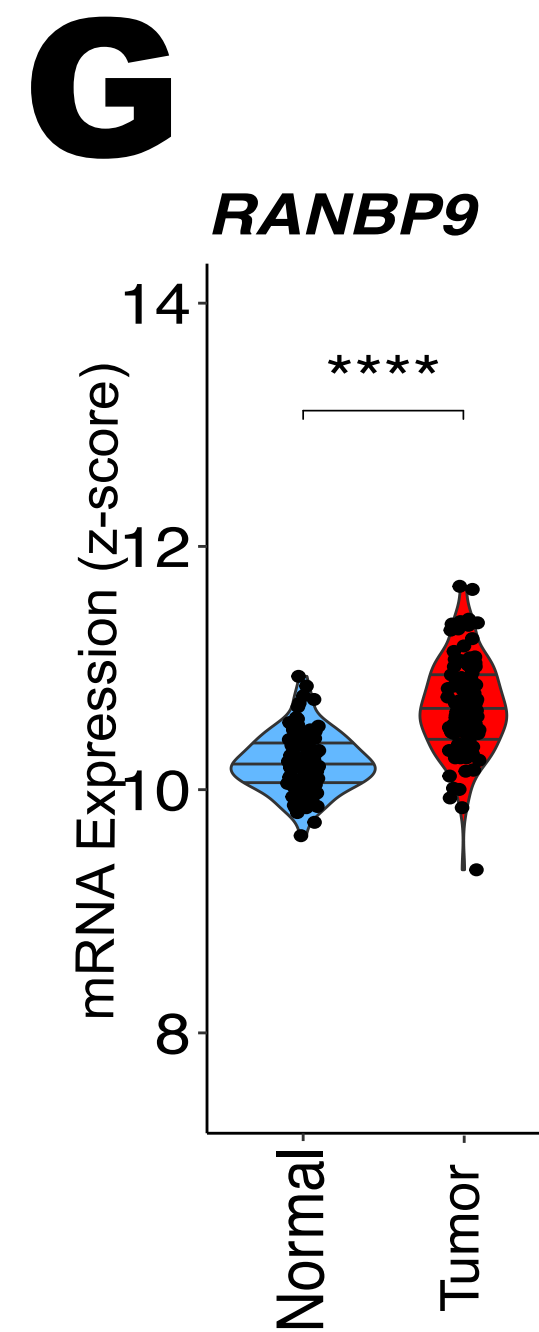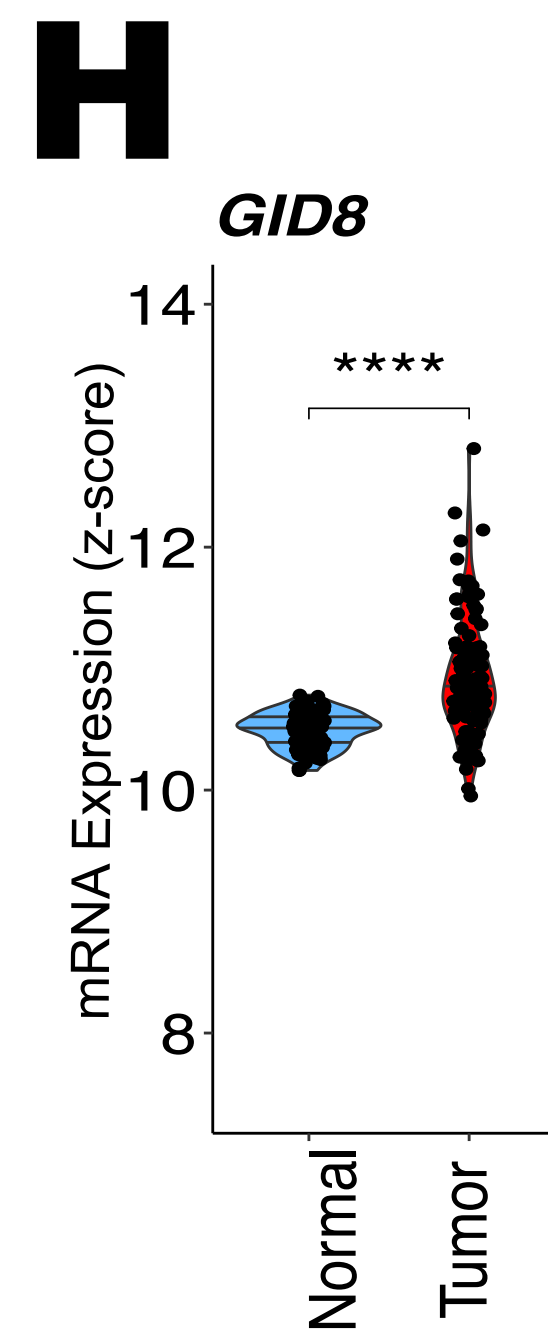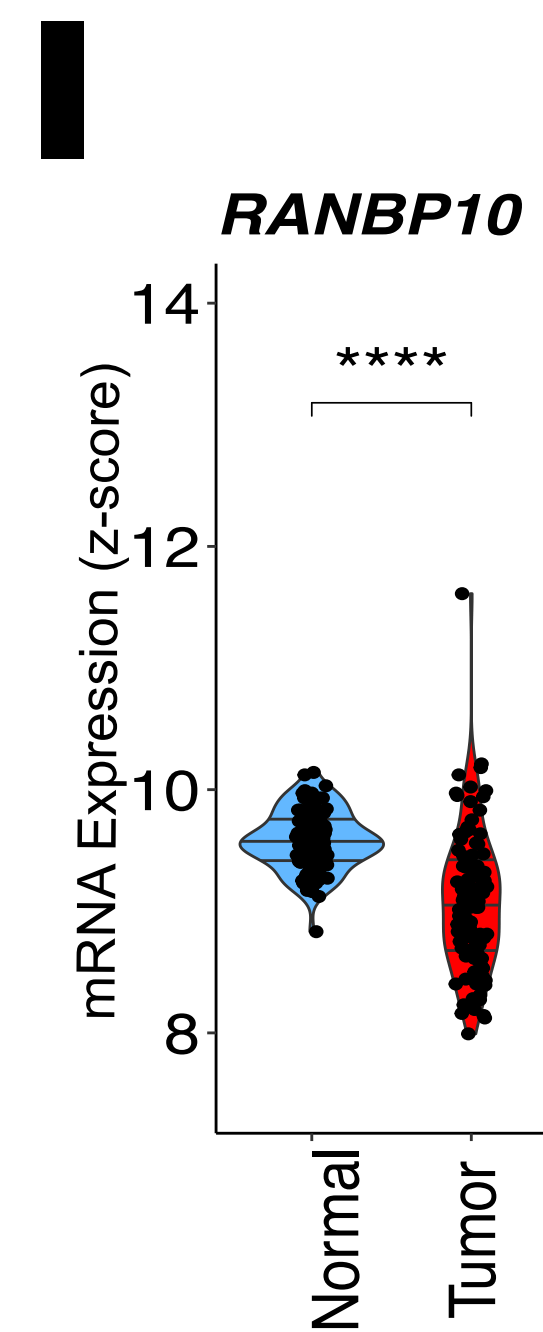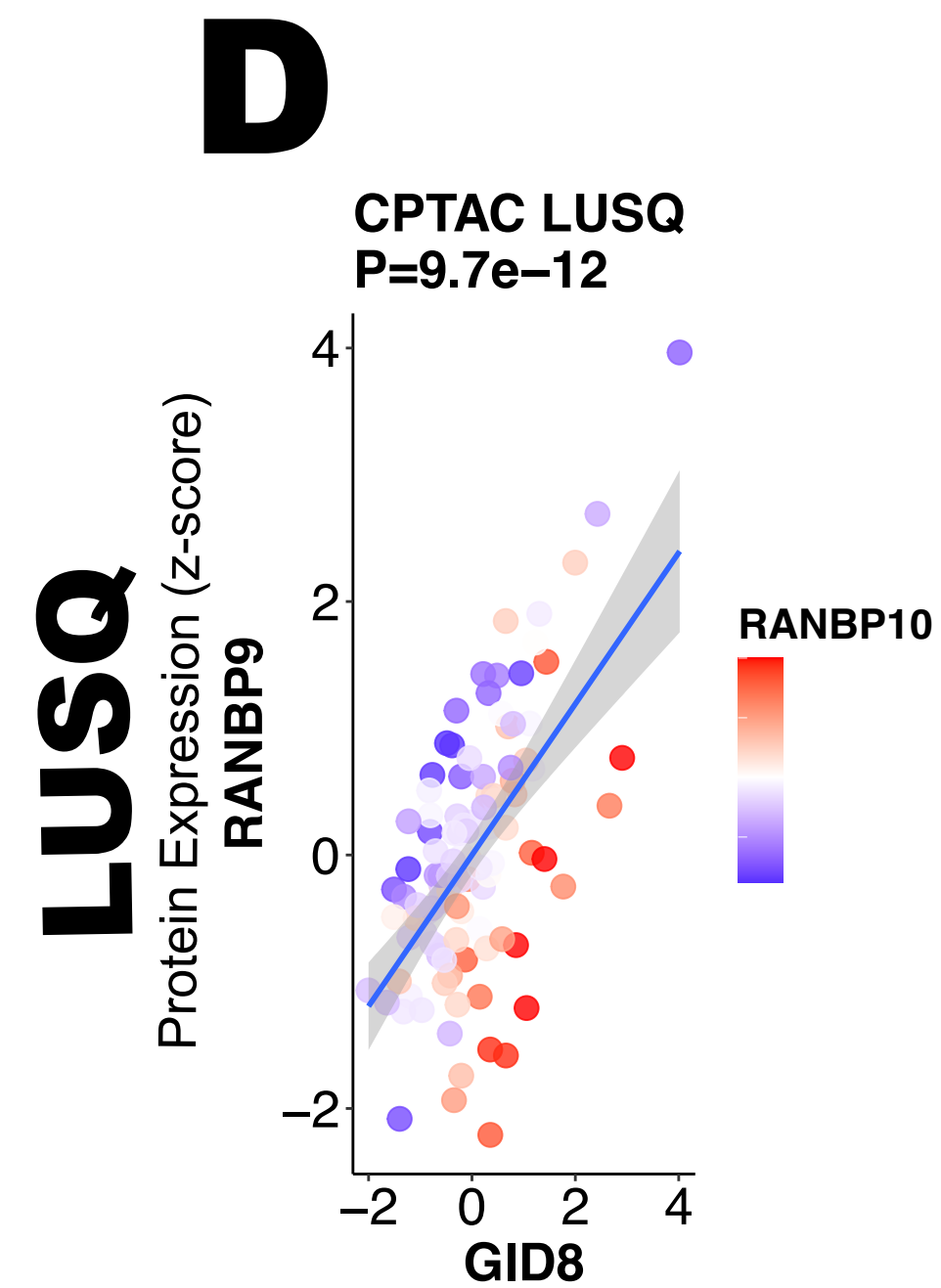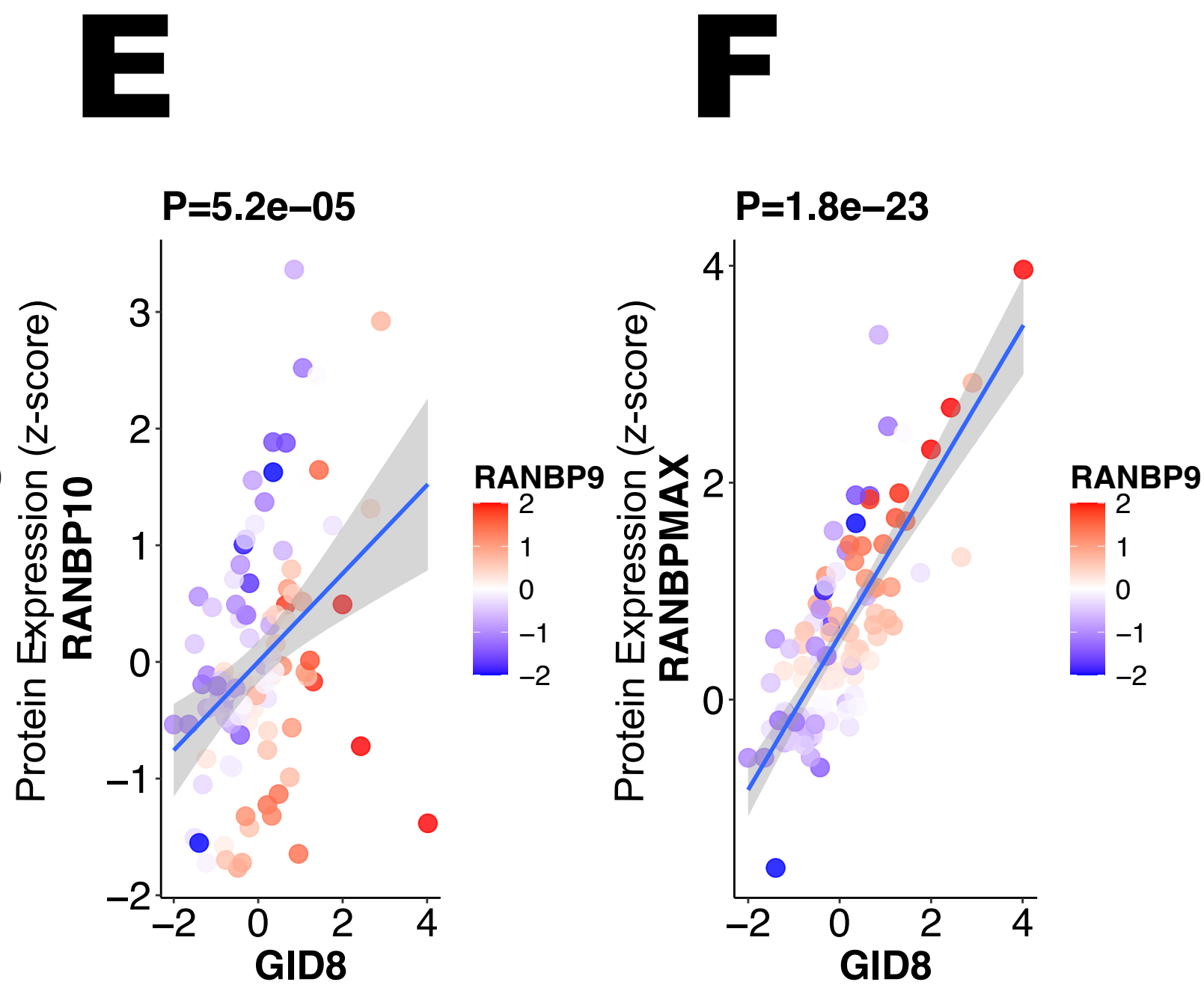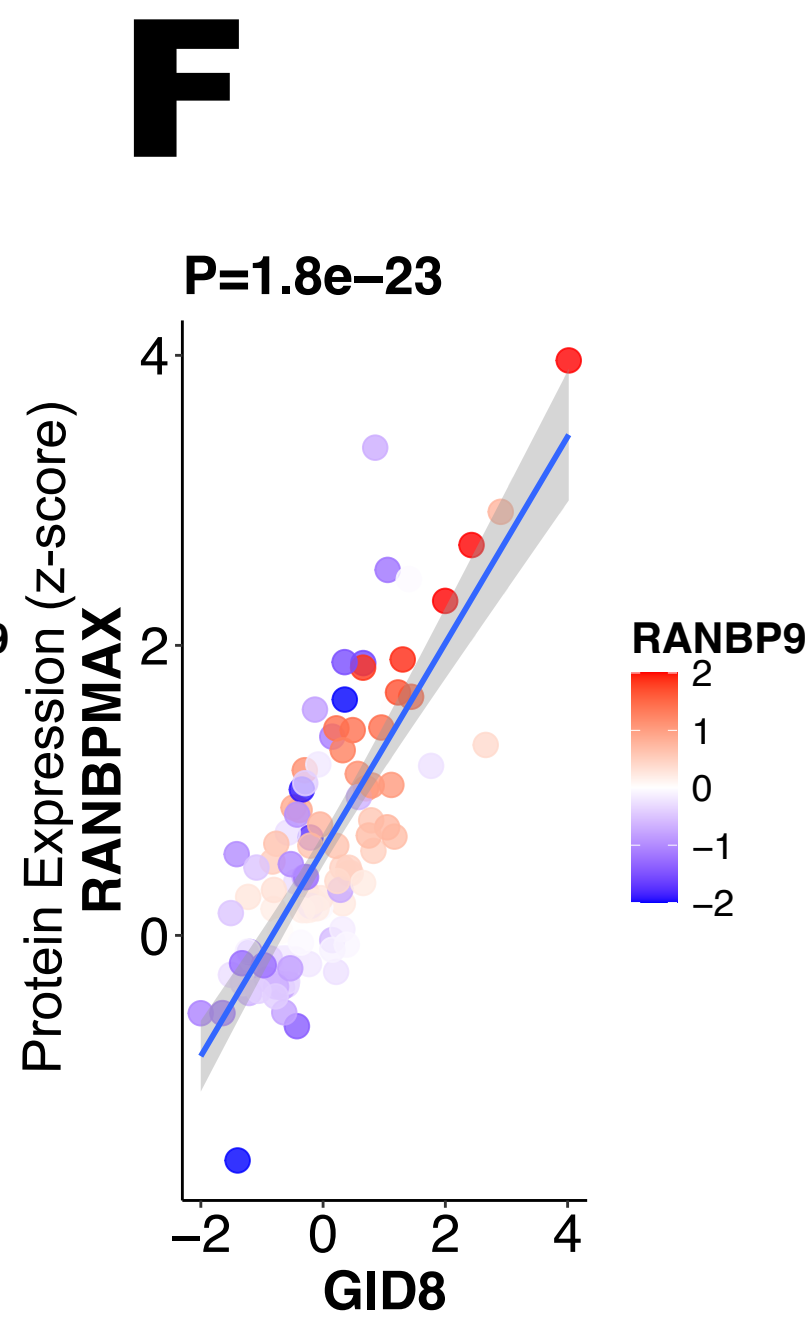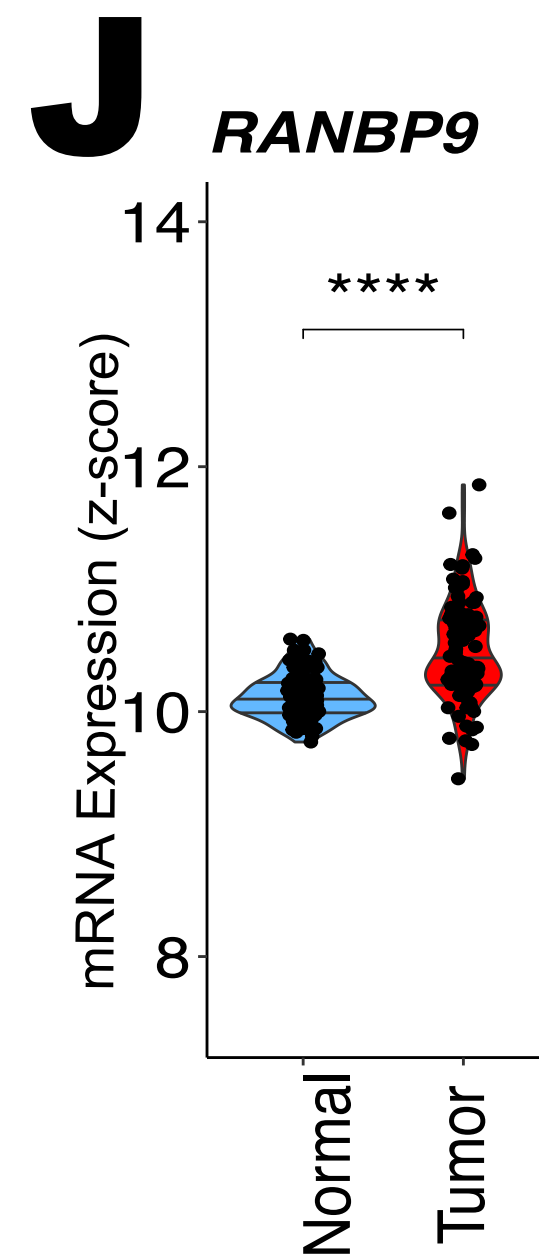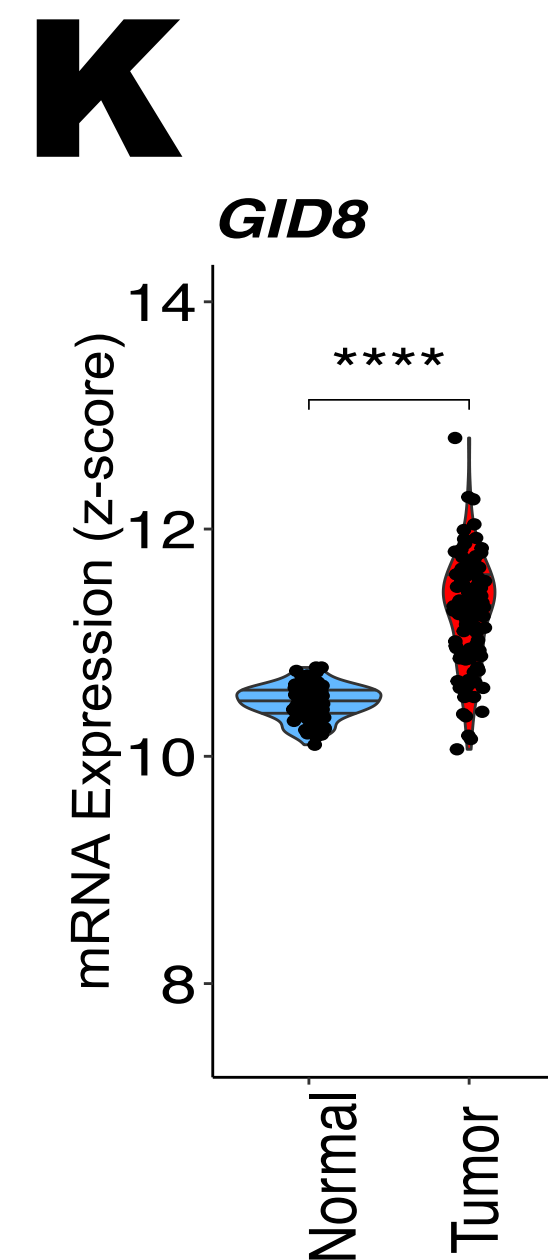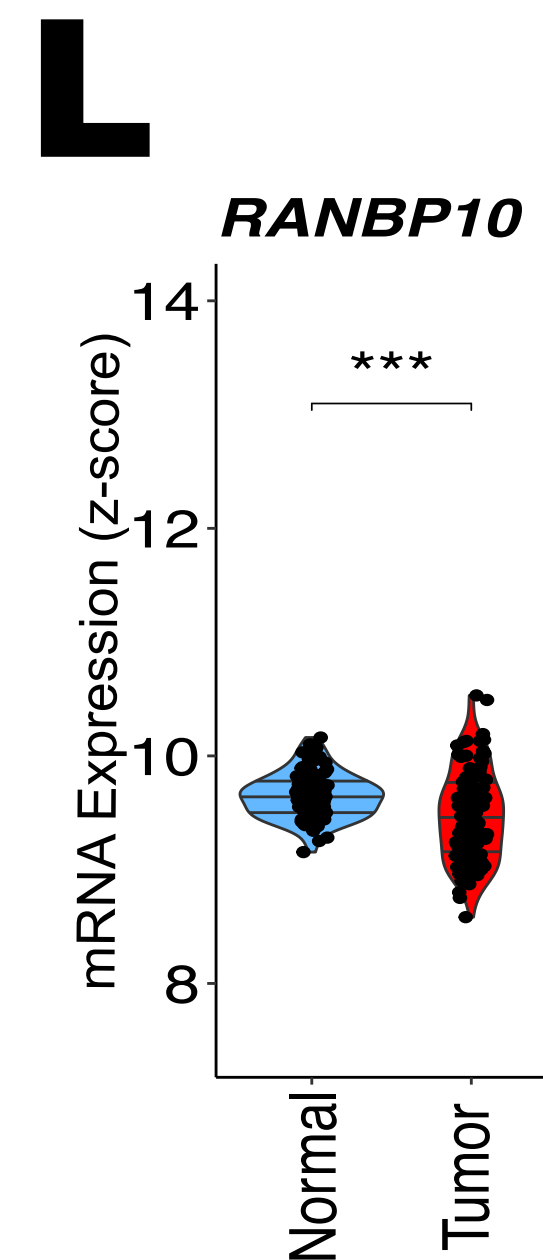

Supplement: Supplementary file 5 — Supplementary Material 5. Supplementary Fig. 5. RANBP9 and GID8 are overexpressed, whereas RanBP10 is downregulated in the CPTAC collection of both LUAD and LUSQ tumors compared with matched normal adjacent tissue. The analysis of the LUAD and LUSQ CPTAC data shows that both the RANBP9 (A, D) and RANBP10 (B, E) proteins significantly correlate with their binding partner GID8. The correlation between the Scorpins and GID8 becomes particularly significant when the maximal expression of either one of the two (RANBPMAX) is used (C, F), illustrating the stoichiometric relationship of the complex. The dots represent single tumors, and the color indicates the level of expression per the color scale shown. When points are below and to the right of the trendline, this indicates high GID8 expression with low RANBP9 expression, which has ‘red’ expression of RANBP10, and vice versa for RANBP10 vs RANBP9. P value from linear regression performed in RStudio. The analysis of the LUAD and LUSQ CPTAC data revealed that both RANBP9 (G, J) and GID8 mRNAs (H, K) are upregulated, whereas the RANBP10 transcript (I, L) is downregulated. The data were downloaded from https://kb.linkedomics.org/. The statistical analysis was performed via RStudio. **** p < 0.001; *** p = 0.001. [file 13046_2025_3491_MOESM5_ESM.pdf]

**A****LUAD**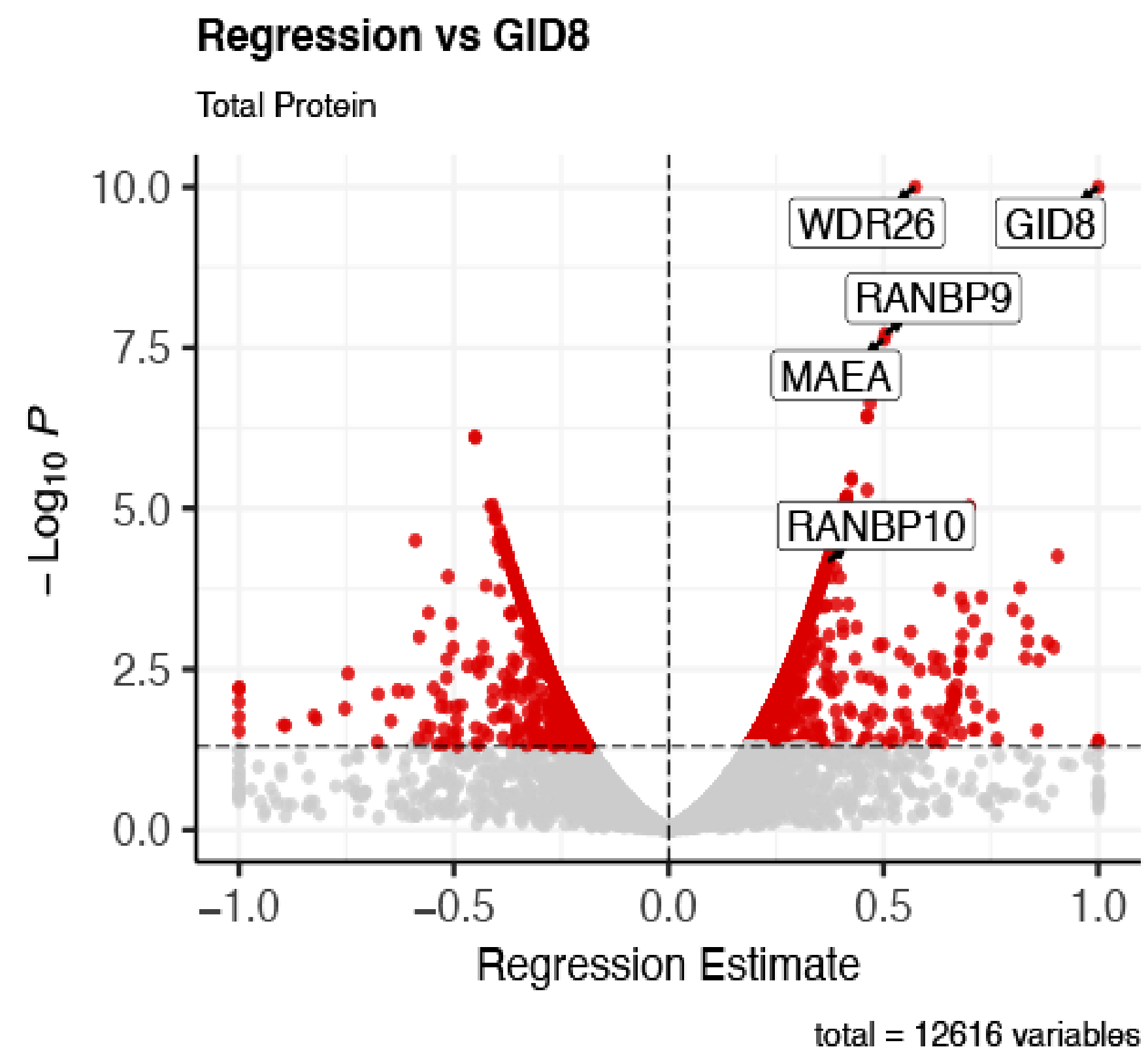**B****LUSQ**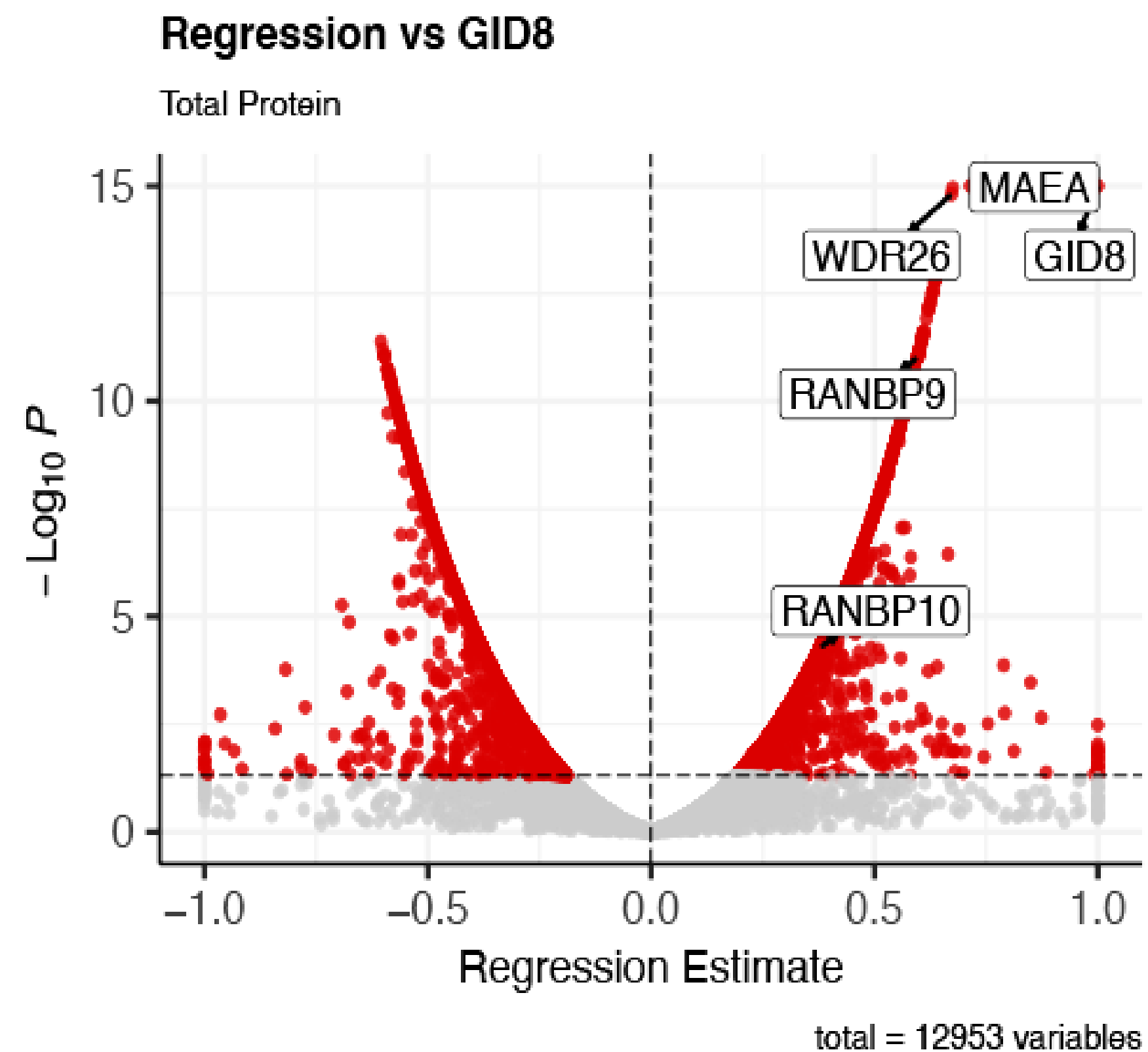

Supplement: Supplementary file 6 — Supplementary Material 6. Supplementary Fig. 6. RANBP9 and RANBP10 expression was positively correlated with GID8 expression in both the LUAD and LUSQ CPTAC datasets. Volcano plots illustrating the linear regression analysis of proteins related to GID8 in the LUAD (A) and LUSQ (B) CPTAC data collections. Protein expression was converted to a Z score, and regression analysis was performed in RStudio. Unadjusted log10-transformed p values on the y-axis are plotted against regression estimates. Values exceeding the plotted ranges are shown at the corresponding maximum or minimum values. The CTLH family members RANBP9, RANBP10, GID8, MAEA, and WRD26 are labeled. [file 13046_2025_3491_MOESM6_ESM.pdf]

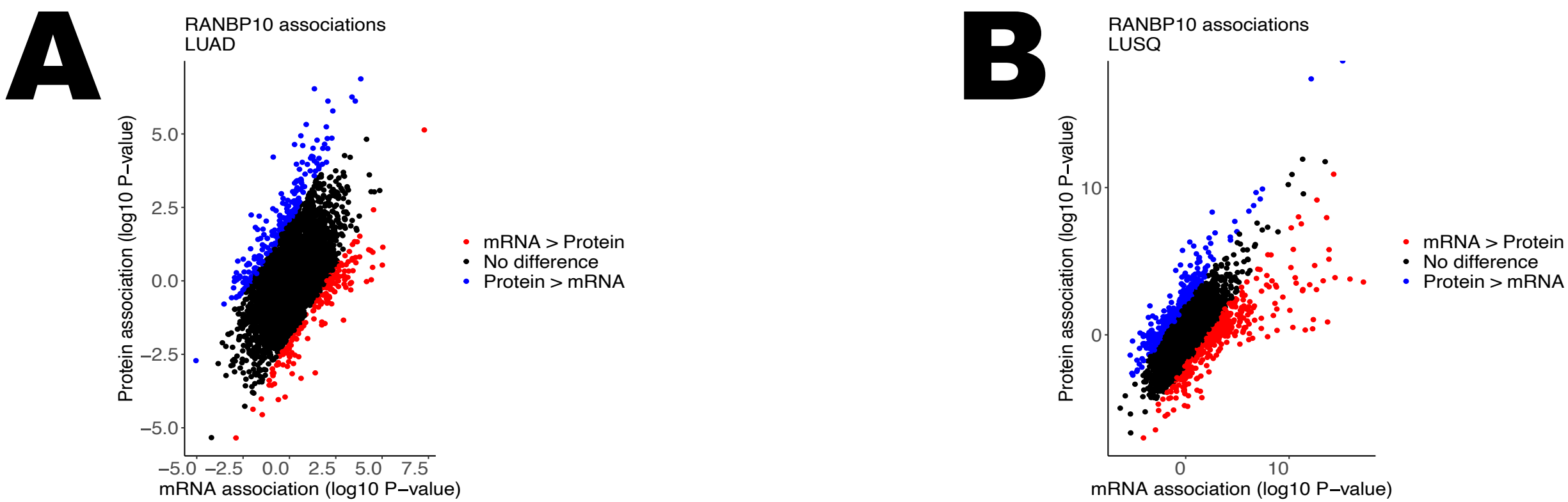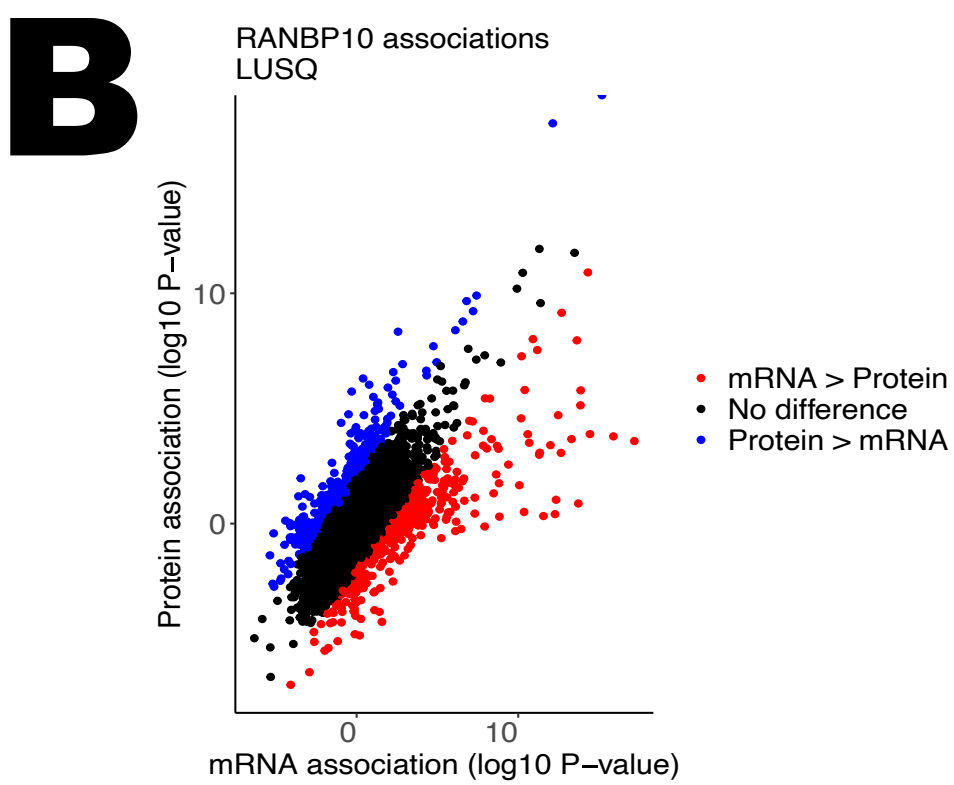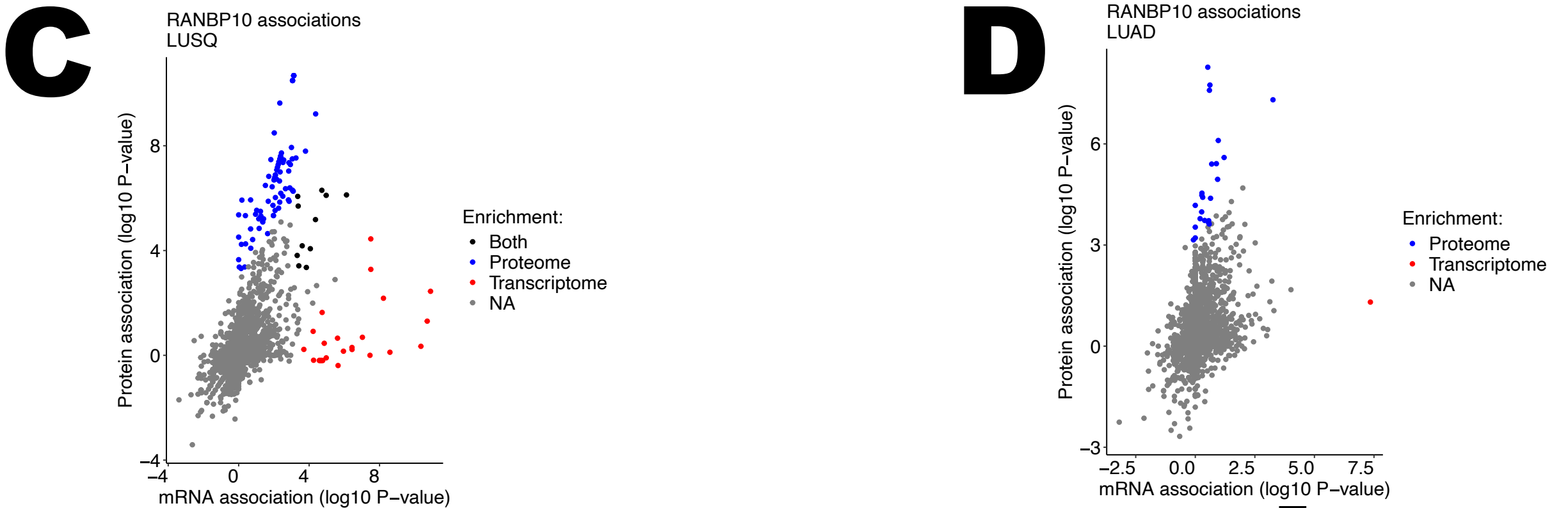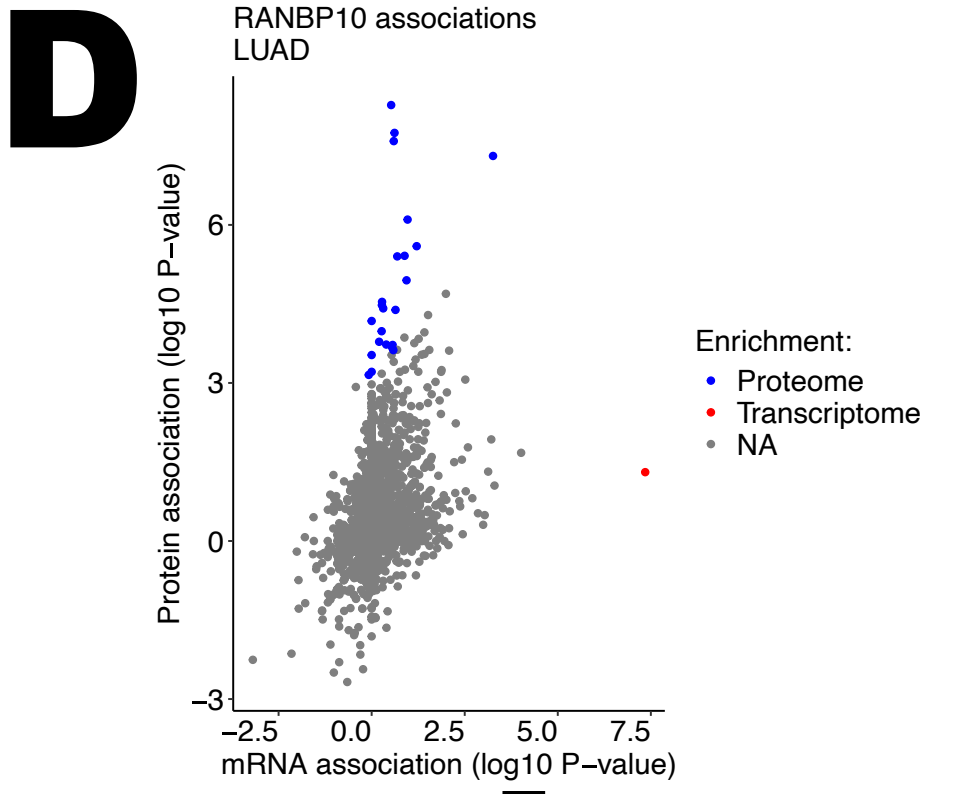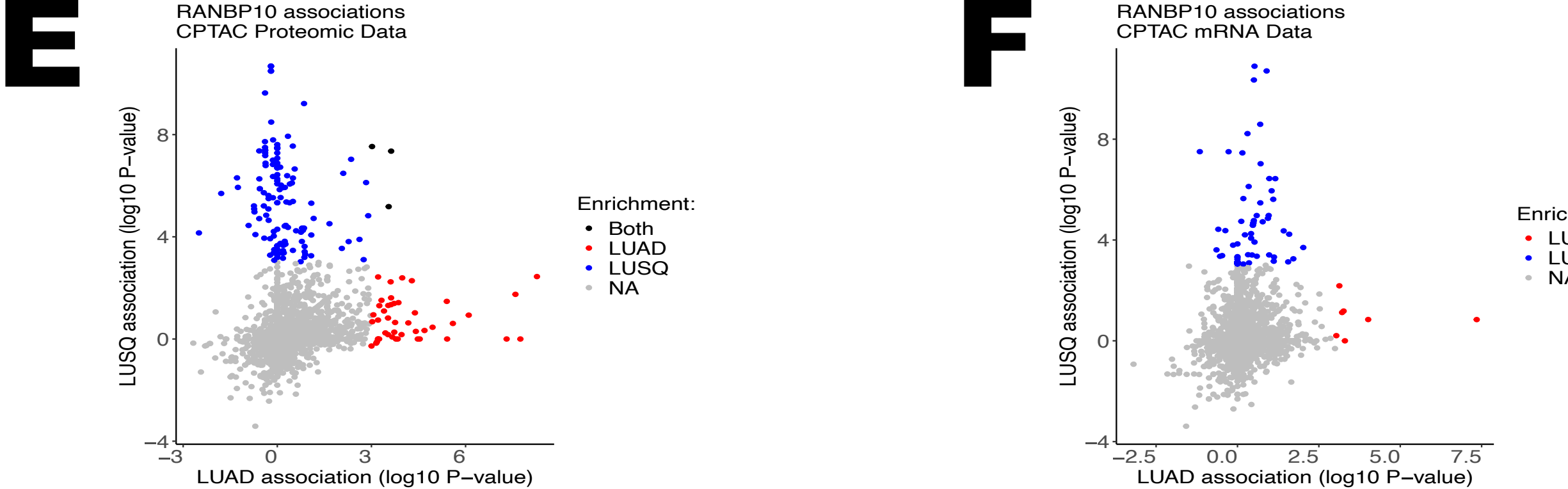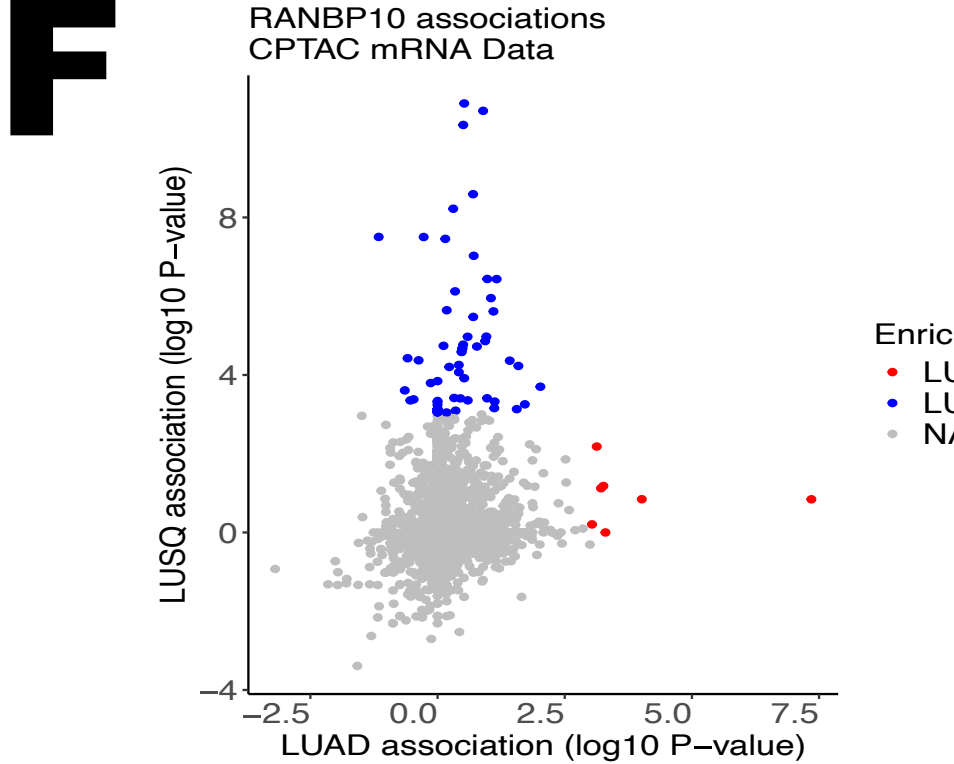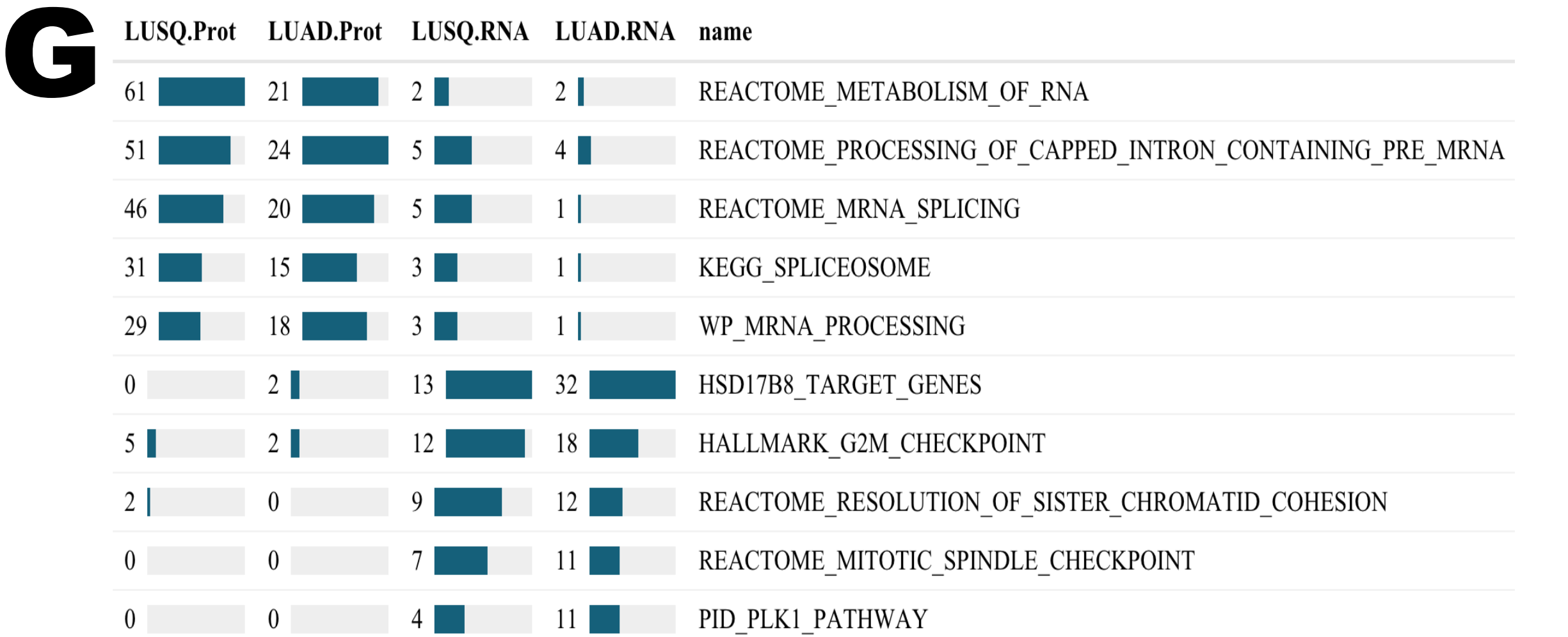

Supplement: Supplementary file 10 — Supplementary Material 10. [file 13046_2025_3491_MOESM10_ESM.pdf]
