# Supplementary material for: RANBP9 and RANBP10 cooperate in regulating non-small cell lung cancer proliferation
Source: J Exp Clin Cancer Res. 2025 Aug 29;44:259. doi: 10.1186/s13046-025-03491-8 (PMC12395873; doi:10.1186/s13046-025-03491-8)
Supplement: Supplementary file 7 — Supplementary Material 7. Supplementary Fig. 7. RANBP9 expression is correlated with cell proliferation according to the CPTAC NSCLC data. The results of the top five gene set enrichment analyses (GSEAs) positively associated with RANBP9 in the CPTAC LUAD (A) and LUSQ (B), positively associated with RANBP10 in the CPTAC LUAD (C) and LUSQ (D), negatively associated with RANBP9 in the CPTAC LUAD (E) and LUSQ (F), and negatively associated with RANBP10 in the CPTAC LUAD (G) and LUSQ (H) datasets were downloaded from the WEB-basedGEne SeT AnaLysis Toolkit (WebGestalt: webgestalt.org). The GSEA results are listed from the top left to the bottom right in descending order of the normalized enrichment score (NES). The LUAD and LUSQ data were used to perform a Metascape analysis for enrichment of Gene Ontology (GO) terms (https://metascape.org; Zhou et al. Nature Comm., 2019) 52. Analysis of proteins positively correlated with RANBP9 expression in LUAD (I) and LUSQ (J), with RANBP10 expression in LUAD (K) and LUSQ (L), or negatively correlated with RANBP9 expression in LUAD (M) and LUSQ (N), and negatively correlated with RANBP10 expression in LUAD (O) and LUSQ (P). Compared with RANBP9, the overexpression of RANBP10 causes different changes in the NSCLC proteome, downregulating several proliferation-associated proteins. (A) Experimental outline of the in vitro experiment used to study the proteome upon Scorpin induction. Scorpin WT iBP9 and iBP10 cell lines were exposed to 1 mg/mL Doxy for 24 h. Quadruplicates of total cell lysates were harvested and processed for analysis via mass spectrometry. (B) Heatmaps showing RANBP9-selective or (C) RANBP10-selective proteins that are differentially expressed after Doxy treatment, corresponding to Fig. 8D. Proteins were considered to be positively associated with RANBP9 or RANBP10 if they were significantly different between the Doxy-treated and control conditions and significantly different in expression vs the opposite Scorpin memb [file 13046_2025_3491_MOESM7_ESM.pdf]

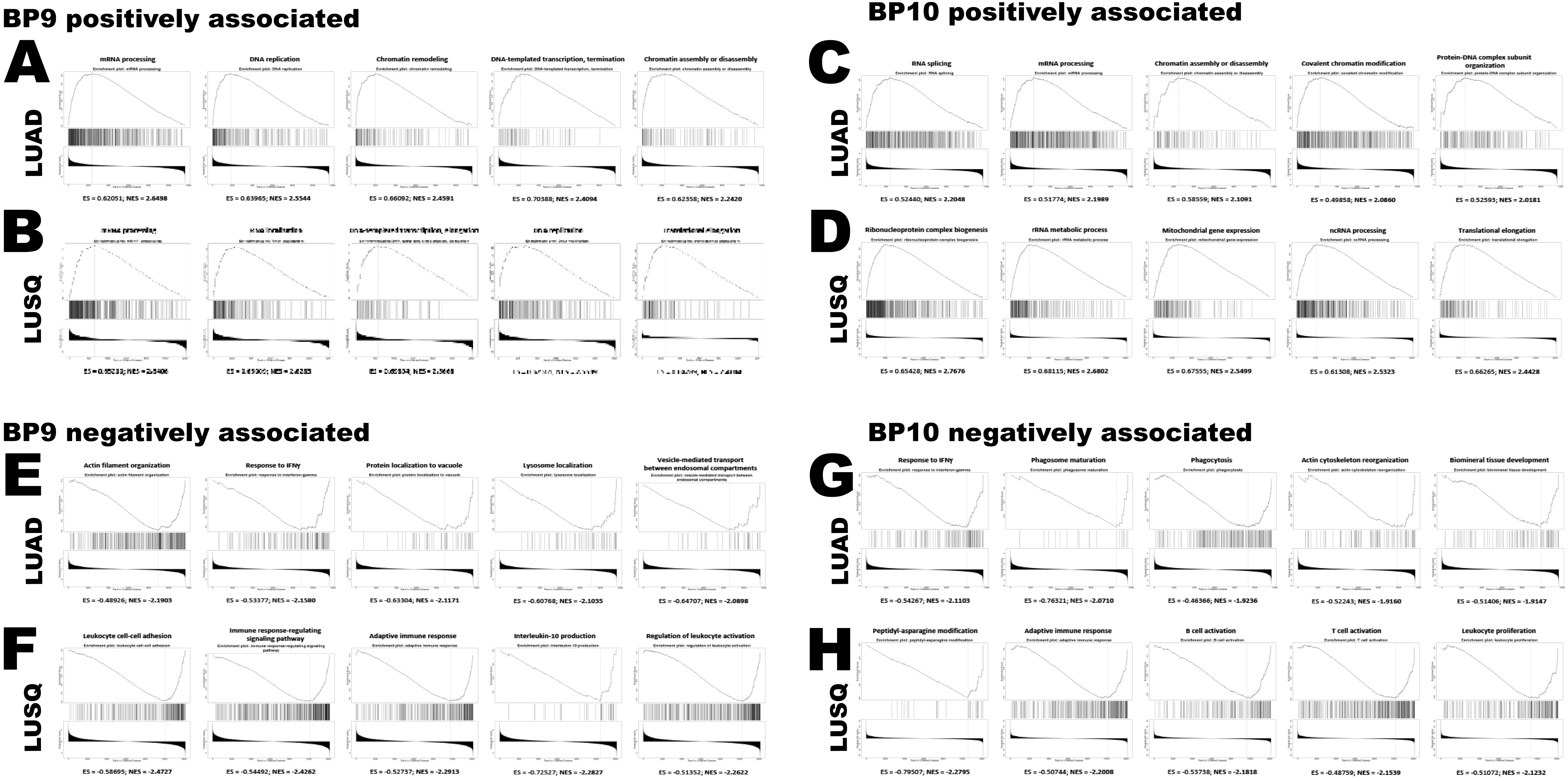
BP9 positively associated
A
LUAD
mRNA processing
ES = 0.62051; NES = 2.6498
DNA replication
ES = 0.63965; NES = 2.5544
Chromatin remodeling
ES = 0.66092; NES = 2.4591
DNA-templated transcription, termination
ES = 0.70388; NES = 2.4094
Chromatin assembly or disassembly
ES = 0.62358; NES = 2.2420
B
LUSQ
BP10 positively associated
C
LUAD
RNA splicing
ES = 0.52440; NES = 2.2048
mRNA processing
ES = 0.51774; NES = 2.1989
Chromatin assembly or disassembly
ES = 0.58559; NES = 2.1091
Covalent chromatin modification
ES = 0.49858; NES = 2.0860
Protein-DNA complex subunit organization
ES = 0.52593; NES = 2.0181
D
LUSQ
Ribonucleoprotein complex biogenesis
ES = 0.65428; NES = 2.7676
rRNA metabolic process
ES = 0.68115; NES = 2.6802
Mitochondrial gene expression
ES = 0.67555; NES = 2.5499
ncRNA processing
ES = 0.61308; NES = 2.5323
Translational elongation
ES = 0.66265; NES = 2.4428
BP9 negatively associated
E
LUAD
Actin filament organization
ES = -0.48926; NES = -2.1903
Response to IFNγ
ES = -0.53377; NES = -2.1580
Protein localization to vacuole
ES = -0.63304; NES = -2.1171
Lysosome localization
ES = -0.60768; NES = -2.1035
Vesicle-mediated transport between endosomal compartments
ES = -0.64707; NES = -2.0898
F
LUSQ
Leukocyte cell-cell adhesion
ES = -0.58695; NES = -2.4727
Immune response-regulating signaling pathway
ES = -0.54492; NES = -2.4262
Adaptive immune response
ES = -0.52737; NES = -2.2913
Interleukin-10 production
ES = -0.72527; NES = -2.2827
Regulation of leukocyte activation
ES = -0.51352; NES = -2.2622
BP10 negatively associated
G
LUAD
Response to IFNγ
ES = -0.54267; NES = -2.1103
Phagosome maturation
ES = -0.76321; NES = -2.0710
Phagocytosis
ES = -0.46366; NES = -1.9236
Actin cytoskeleton reorganization
ES = -0.52243; NES = -1.9160
Biomineral tissue development
ES = -0.51406; NES = -1.9147
H
LUSQ
Peptidyl-asparagine modification
ES = -0.79507; NES = -2.2795
Adaptive immune response
ES = -0.50744; NES = -2.2008
B cell activation
ES = -0.53738; NES = -2.1818
T cell activation
ES = -0.48759; NES = -2.1539
Leukocyte proliferation
ES = -0.51072; NES = -2.1232

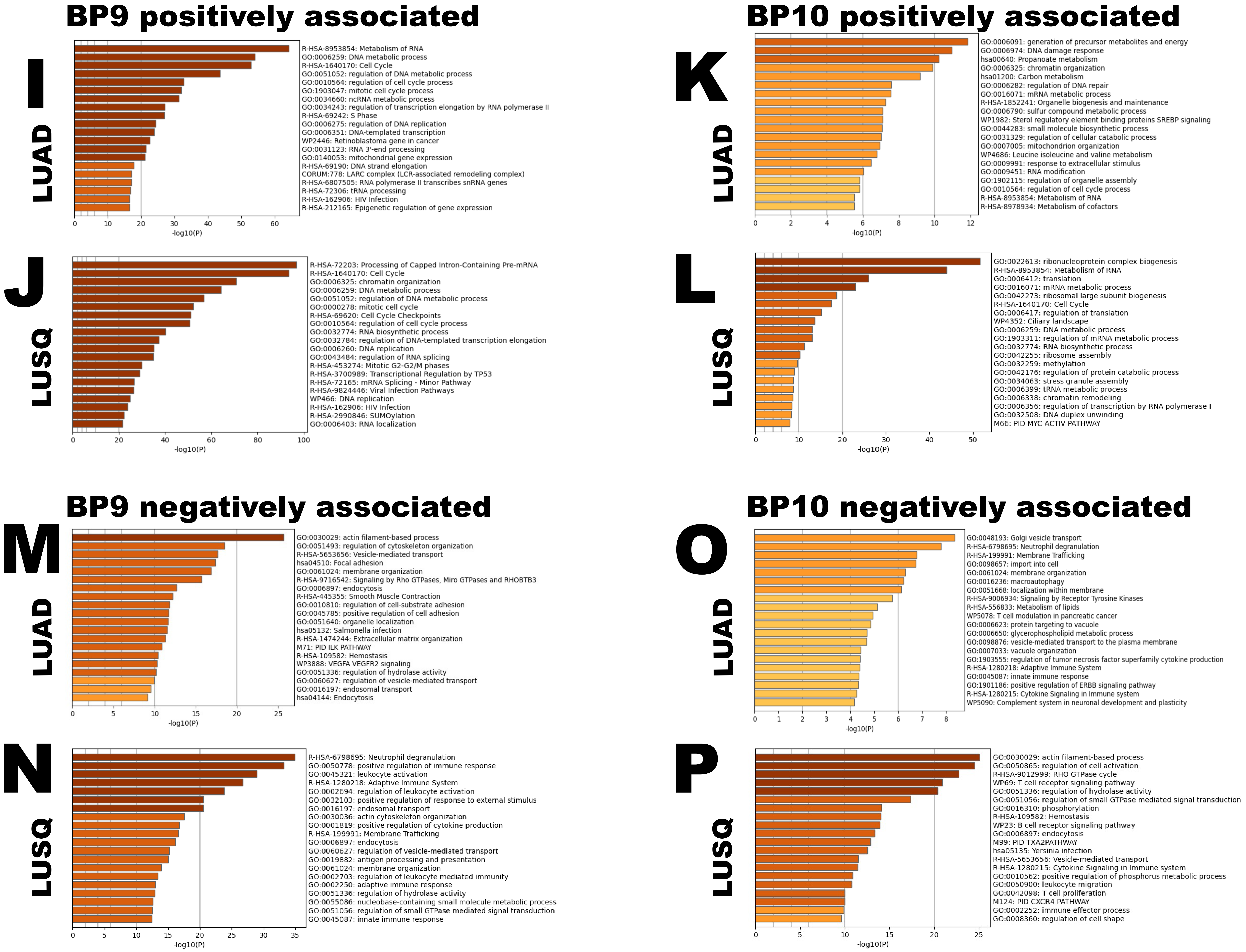
BP9 positively associated
I
LUAD
R-HSA-8953854: Metabolism of RNA
GO:0006259: DNA metabolic process
R-HSA-1640170: Cell Cycle
GO:0051052: regulation of DNA metabolic process
GO:0010564: regulation of cell cycle process
GO:1903047: mitotic cell cycle process
GO:0034660: ncRNA metabolic process
GO:0034243: regulation of transcription elongation by RNA polymerase II
R-HSA-69242: S Phase
GO:0006275: regulation of DNA replication
GO:0006351: DNA-templated transcription
WP2446: Retinoblastoma gene in cancer
GO:0031123: RNA 3'-end processing
GO:0140053: mitochondrial gene expression
R-HSA-69190: DNA strand elongation
CORUM:778: LARC complex (LCR-associated remodeling complex)
R-HSA-6807505: RNA polymerase II transcribes snRNA genes
R-HSA-72306: tRNA processing
R-HSA-162906: HIV Infection
R-HSA-212165: Epigenetic regulation of gene expression
-log10(P)
J
LUSQ
R-HSA-72203: Processing of Capped Intron-Containing Pre-mRNA
R-HSA-1640170: Cell Cycle
GO:0006325: chromatin organization
GO:0006259: DNA metabolic process
GO:0051052: regulation of DNA metabolic process
GO:0000278: mitotic cell cycle
R-HSA-69620: Cell Cycle Checkpoints
GO:0010564: regulation of cell cycle process
GO:0032774: RNA biosynthetic process
GO:0032784: regulation of DNA-templated transcription elongation
GO:0006260: DNA replication
GO:0043484: regulation of RNA splicing
R-HSA-453274: Mitotic G2-G2/M phases
R-HSA-3700989: Transcriptional Regulation by TP53
R-HSA-72165: mRNA Splicing - Minor Pathway
R-HSA-9824446: Viral Infection Pathways
WP466: DNA replication
R-HSA-162906: HIV Infection
R-HSA-2990846: SUMOylation
GO:0006403: RNA localization
-log10(P)
BP10 positively associated
K
LUAD
GO:0006091: generation of precursor metabolites and energy
GO:0006974: DNA damage response
hsa00640: Propanoate metabolism
GO:0006325: chromatin organization
hsa01200: Carbon metabolism
GO:0006282: regulation of DNA repair
GO:0016071: mRNA metabolic process
R-HSA-1852241: Organelle biogenesis and maintenance
GO:0006790: sulfur compound metabolic process
WP1982: Sterol regulatory element binding proteins SREBP signaling
GO:0044283: small molecule biosynthetic process
GO:0031329: regulation of cellular catabolic process
GO:0007005: mitochondrion organization
WP4686: Leucine isoleucine and valine metabolism
GO:0009991: response to extracellular stimulus
GO:0009451: RNA modification
GO:1902115: regulation of organelle assembly
GO:0010564: regulation of cell cycle process
R-HSA-8953854: Metabolism of RNA
R-HSA-8978934: Metabolism of cofactors
-log10(P)
L
LUSQ
GO:0022613: ribonucleoprotein complex biogenesis
R-HSA-8953854: Metabolism of RNA
GO:0006412: translation
GO:0016071: mRNA metabolic process
GO:0042273: ribosomal large subunit biogenesis
R-HSA-1640170: Cell Cycle
GO:0006417: regulation of translation
WP4352: Ciliary landscape
GO:0006259: DNA metabolic process
GO:1903311: regulation of mRNA metabolic process
GO:0032774: RNA biosynthetic process
GO:0042255: ribosome assembly
GO:0032259: methylation
GO:0042176: regulation of protein catabolic process
GO:0034063: stress granule assembly
GO:0006399: tRNA metabolic process
GO:0006338: chromatin remodeling
GO:0006356: regulation of transcription by RNA polymerase I
GO:0032508: DNA duplex unwinding
M66: PID MYC ACTIV PATHWAY
-log10(P)
BP9 negatively associated
M
LUAD
GO:0030029: actin filament-based process
GO:0051493: regulation of cytoskeleton organization
R-HSA-5653656: Vesicle-mediated transport
hsa04510: Focal adhesion
GO:0061024: membrane organization
R-HSA-9716542: Signaling by Rho GTPases, Miro GTPases and RHOBTB3
GO:0006897: endocytosis
R-HSA-445355: Smooth Muscle Contraction
GO:0010810: regulation of cell-substrate adhesion
GO:0045785: positive regulation of cell adhesion
GO:0051640: organelle localization
hsa05132: Salmonella infection
R-HSA-1474244: Extracellular matrix organization
M71: PID ILK PATHWAY
R-HSA-109582: Hemostasis
WP3888: VEGFA VEGFR2 signaling
GO:0051336: regulation of hydrolase activity
GO:0060627: regulation of vesicle-mediated transport
GO:0016197: endosomal transport
hsa04144: Endocytosis
-log10(P)
N
LUSQ
R-HSA-6798695: Neutrophil degranulation
GO:0050778: positive regulation of immune response
GO:0045321: leukocyte activation
R-HSA-1280218: Adaptive Immune System
GO:0002694: regulation of leukocyte activation
GO:0032103: positive regulation of response to external stimulus
GO:0016197: endosomal transport
GO:0030036: actin cytoskeleton organization
GO:0001819: positive regulation of cytokine production
R-HSA-199991: Membrane Trafficking
GO:0006897: endocytosis
GO:0060627: regulation of vesicle-mediated transport
GO:0019882: antigen processing and presentation
GO:0061024: membrane organization
GO:0002703: regulation of leukocyte mediated immunity
GO:0002250: adaptive immune response
GO:0051336: regulation of hydrolase activity
GO:0055086: nucleobase-containing small molecule metabolic process
GO:0051056: regulation of small GTPase mediated signal transduction
GO:0045087: innate immune response
-log10(P)
BP10 negatively associated
O
LUAD
GO:0048193: Golgi vesicle transport
R-HSA-6798695: Neutrophil degranulation
R-HSA-199991: Membrane Trafficking
GO:0098657: import into cell
GO:0061024: membrane organization
GO:0016236: macroautophagy
GO:0051668: localization within membrane
R-HSA-9006934: Signaling by Receptor Tyrosine Kinases
R-HSA-556833: Metabolism of lipids
WP5078: T cell modulation in pancreatic cancer
GO:0006623: protein targeting to vacuole
GO:0006650: glycerophospholipid metabolic process
GO:0098876: vesicle-mediated transport to the plasma membrane
GO:0007033: vacuole organization
GO:1903555: regulation of tumor necrosis factor superfamily cytokine production
R-HSA-1280218: Adaptive Immune System
GO:0045087: innate immune response
GO:1901186: positive regulation of ERBB signaling pathway
R-HSA-1280215: Cytokine Signaling in Immune system
WP5090: Complement system in neuronal development and plasticity
-log10(P)
P
LUSQ
GO:0030029: actin filament-based process
GO:0050865: regulation of cell activation
R-HSA-9012999: RHO GTPase cycle
WP69: T cell receptor signaling pathway
GO:0051336: regulation of hydrolase activity
GO:0051056: regulation of small GTPase mediated signal transduction
GO:0016310: phosphorylation
R-HSA-109582: Hemostasis
WP23: B cell receptor signaling pathway
GO:0006897: endocytosis
M99: PID TXA2PATHWAY
hsa05135: Yersinia infection
R-HSA-5653656: Vesicle-mediated transport
R-HSA-1280215: Cytokine Signaling in Immune system
GO:0010562: positive regulation of phosphorus metabolic process
GO:0050900: leukocyte migration
GO:0042098: T cell proliferation
M124: PID CXCR4 PATHWAY
GO:0002252: immune effector process
GO:0008360: regulation of cell shape
-log10(P)
