# Supplementary material for: RANBP9 and RANBP10 cooperate in regulating non-small cell lung cancer proliferation
Source: J Exp Clin Cancer Res. 2025 Aug 29;44:259. doi: 10.1186/s13046-025-03491-8 (PMC12395873; doi:10.1186/s13046-025-03491-8)
Supplement: Supplementary file 8 — Supplementary Material 8. Supplementary Fig. 8. Compared with RANBP9, the overexpression of RANBP10 causes different changes in the NSCLC ubiquitylome, which includes proliferation-associated proteins. (A) Experimental outline of the in vitro experiment used to study the ubiquitylome upon Scorpin induction. Scorpin WT iBP9 and iBP10 cell lines were exposed to 1 mg/mL doxycycline for 24 h and to the proteasome inhibitor MG132 (10 mg/mL) for the last 4 h. Quadruplicates of total cell lysates were harvested and processed for analysis via mass spectrometry. B-E) Metascape analysis of ubiquitylated proteins in iA549 cells associated positively with RANBP9 (B), negatively with RANBP9 (C), positively with RANBP10 (D), and negatively with RANBP10 (E). The analysis was performed at https://metascape.org (Zhou et al. Nature Comm., 2019) 52. (F) Bar graph reporting the number of proliferation-associated proteins (PAPs 53) found in total (gray bar), in the list of proteins positively associated with RANBP10 (blue bar), and in the list of proteins positively associated with RANBP9 (red bar), setting the statistical threshold at p = 0.1. This latter group was further analyzed for the presence of specific PPA ubiquitylations that were increased in positive association with RANBP9 (RANBP9UbiqUp), in negative association with RANBP10 (RANBP10UbiquDown) in negative association with RANBP9 (RANBP9UbiquDown) and in positive association with RANBP10 (RANBP10UbiquUp). Fisher’s test was used to assess the statistical significance of the findings. OR = odds ratio. G) Proliferation associated proteins coimmunoprecipitate with RANBP10. V5-tagged endogenous RANBP9 expression was induced using Doxy at 10 ng/mL. After 24 h 1 mg of total cell lysate was immunoprecipitated by primary antibodies to pull-down POLR2B, PABPC1, CACYBP, MCM5, and MCM7. Rabbit IgG pull-down was used as negative control, and GID8 pull-down was used as positive control. The presence of RANBP9 was detected by HRP-conjugate [file 13046_2025_3491_MOESM8_ESM.pdf]

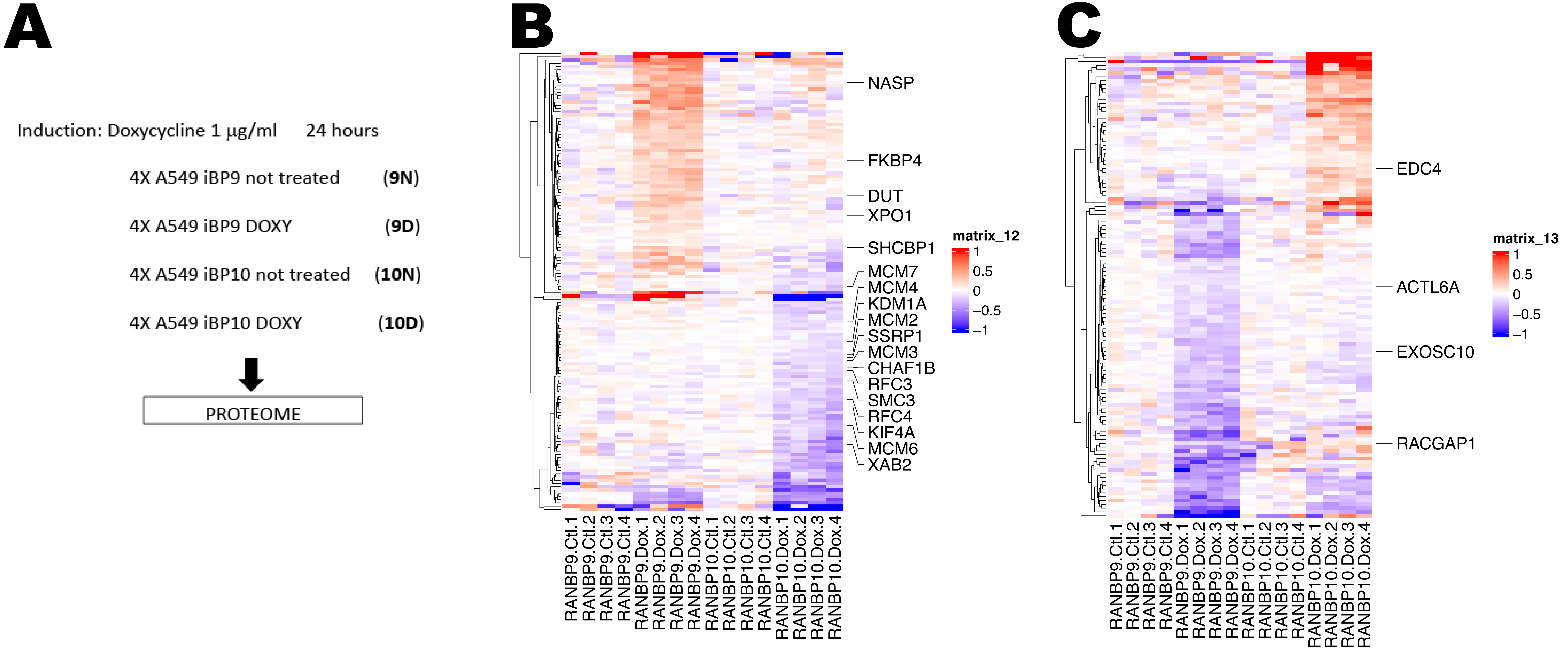
A
Induction: Doxycycline 1 µg/ml 24 hours
4X A549 iBP9 not treated (9N)
4X A549 iBP9 DOXY (9D)
4X A549 iBP10 not treated (10N)
4X A549 iBP10 DOXY (10D)
PROTEOME
B
NASP
FKBP4
DUT
XPO1
SHCBP1
MCM7
MCM4
KDM1A
MCM2
SSRP1
MCM3
CHAF1B
RFC3
SMC3
RFC4
KIF4A
MCM6
XAB2
matrix_12
1
0.5
0
−0.5
−1
RANBP9.Ctl.1
RANBP9.Ctl.2
RANBP9.Ctl.3
RANBP9.Ctl.4
RANBP9.Dox.1
RANBP9.Dox.2
RANBP9.Dox.3
RANBP9.Dox.4
RANBP10.Ctl.1
RANBP10.Ctl.2
RANBP10.Ctl.3
RANBP10.Ctl.4
RANBP10.Dox.1
RANBP10.Dox.2
RANBP10.Dox.3
RANBP10.Dox.4
C
EDC4
ACTL6A
EXOSC10
RACGAP1
matrix_13
1
0.5
0
−0.5
−1
RANBP9.Ctl.1
RANBP9.Ctl.2
RANBP9.Ctl.3
RANBP9.Ctl.4
RANBP9.Dox.1
RANBP9.Dox.2
RANBP9.Dox.3
RANBP9.Dox.4
RANBP10.Ctl.1
RANBP10.Ctl.2
RANBP10.Ctl.3
RANBP10.Ctl.4
RANBP10.Dox.1
RANBP10.Dox.2
RANBP10.Dox.3
RANBP10.Dox.4
