# Supplementary material for: RANBP9 and RANBP10 cooperate in regulating non-small cell lung cancer proliferation
Source: J Exp Clin Cancer Res. 2025 Aug 29;44:259. doi: 10.1186/s13046-025-03491-8 (PMC12395873; doi:10.1186/s13046-025-03491-8)
Supplement: Supplementary file 9 — Supplementary Material 9. Supplementary Fig. 9. RANBP9 regulates key oncogenic pathways preferentially at protein or at transcript level. Analysis of individual mRNAs vs. proteins from CPTAC LUAD (A) and LUSQ (B) showing the linear regression vs. RANBP10 protein. The p-value of two orders of magnitude established arbitrarily using the numeric difference defines genes having a stronger mRNA (red) or protein (blue) association with RANBP10. Pathway analysis in LUAD (C) and LUSQ (D) shows that certain pathways have a stronger RANBP10 association with proteomic (blue) rather than transcriptomic (red) data while other have the opposite trend. The comparison of proteomic of LUAD vs LUSQ (E) and transcriptomic (F) analyses fails to show concordance between the two different CPTAC lung cancer collections. G) Key pathways showing the RANBP9 top 5 predominantly mRNA associations and top 5 predominantly proteomic associations. The RANBP9 proteomic associations focus strongly on RNA metabolism including the spliceosome. [file 13046_2025_3491_MOESM9_ESM.pdf]

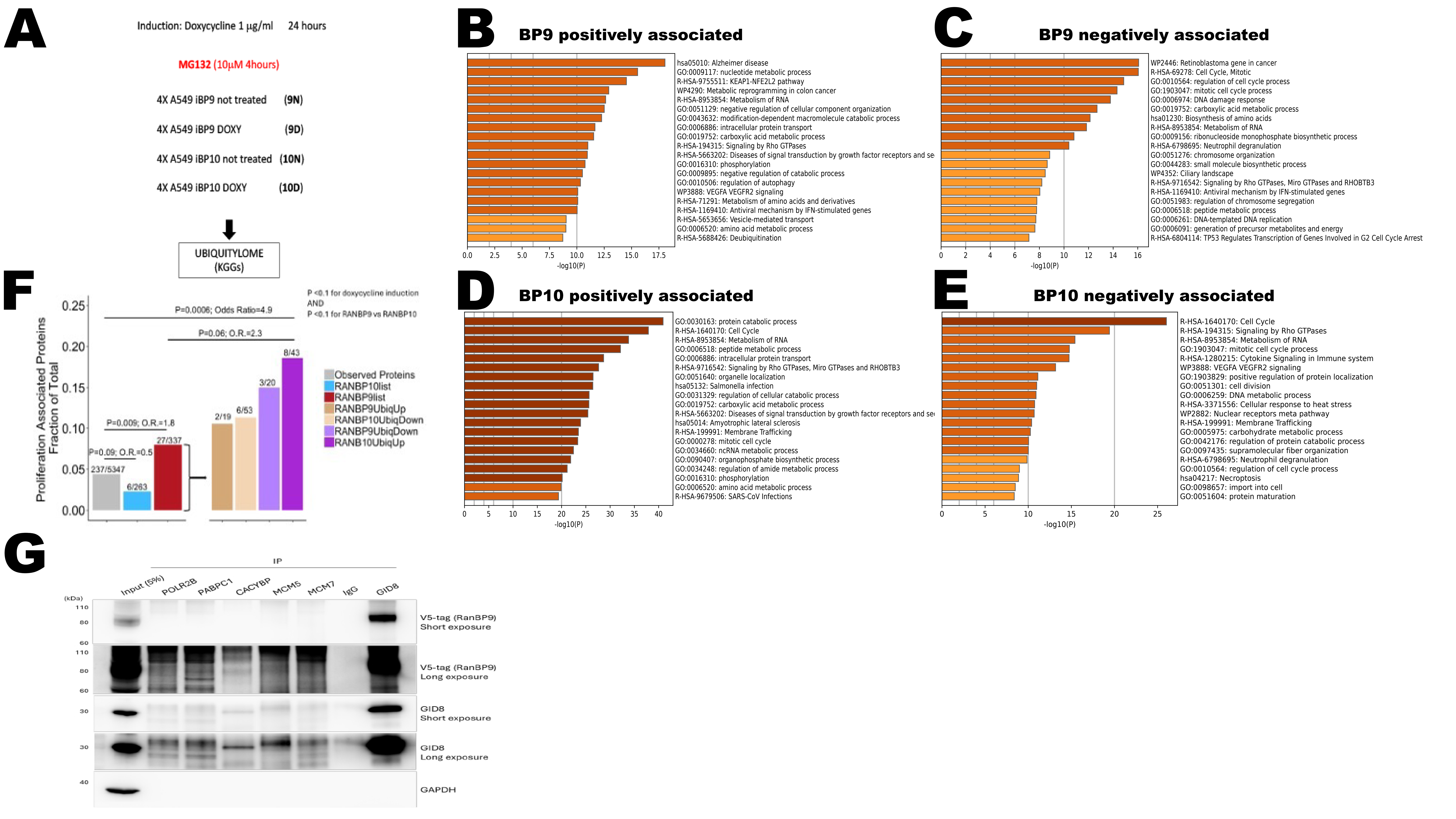
A
Induction: Doxycycline 1 μg/ml 24 hours
MG132 (10μM 4hours)
4X A549 iBP9 not treated (9N)
4X A549 iBP9 DOXY (9D)
4X A549 iBP10 not treated (10N)
4X A549 iBP10 DOXY (10D)
UBIQUITYLOME (KGGs)
B
BP9 positively associated
hsa05010: Alzheimer disease
GO:0009117: nucleotide metabolic process
R-HSA-9755511: KEAP1-NFE2L2 pathway
WP4290: Metabolic reprogramming in colon cancer
R-HSA-8953854: Metabolism of RNA
GO:0051129: negative regulation of cellular component organization
GO:0043632: modification-dependent macromolecule catabolic process
GO:0006886: intracellular protein transport
GO:0019752: carboxylic acid metabolic process
R-HSA-194315: Signaling by Rho GTPases
R-HSA-5663202: Diseases of signal transduction by growth factor receptors and se
GO:0016310: phosphorylation
GO:0009895: negative regulation of catabolic process
GO:0010506: regulation of autophagy
WP3888: VEGFA VEGFR2 signaling
R-HSA-71291: Metabolism of amino acids and derivatives
R-HSA-1169410: Antiviral mechanism by IFN-stimulated genes
R-HSA-5653656: Vesicle-mediated transport
GO:0006520: amino acid metabolic process
R-HSA-5688426: Deubiquitination
-log10(P)
C
BP9 negatively associated
WP2446: Retinoblastoma gene in cancer
R-HSA-69278: Cell Cycle, Mitotic
GO:0010564: regulation of cell cycle process
GO:1903047: mitotic cell cycle process
GO:0006974: DNA damage response
GO:0019752: carboxylic acid metabolic process
hsa01230: Biosynthesis of amino acids
R-HSA-8953854: Metabolism of RNA
GO:0009156: ribonucleoside monophosphate biosynthetic process
R-HSA-6798695: Neutrophil degranulation
GO:0051276: chromosome organization
GO:0044283: small molecule biosynthetic process
WP4352: Ciliary landscape
R-HSA-9716542: Signaling by Rho GTPases, Miro GTPases and RHOBTB3
R-HSA-1169410: Antiviral mechanism by IFN-stimulated genes
GO:0051983: regulation of chromosome segregation
GO:0006518: peptide metabolic process
GO:0006261: DNA-templated DNA replication
GO:0006091: generation of precursor metabolites and energy
R-HSA-6804114: TP53 Regulates Transcription of Genes Involved in G2 Cell Cycle Arrest
-log10(P)
D
BP10 positively associated
GO:0030163: protein catabolic process
R-HSA-1640170: Cell Cycle
R-HSA-8953854: Metabolism of RNA
GO:0006518: peptide metabolic process
GO:0006886: intracellular protein transport
R-HSA-9716542: Signaling by Rho GTPases, Miro GTPases and RHOBTB3
GO:0051640: organelle localization
hsa05132: Salmonella infection
GO:0031329: regulation of cellular catabolic process
GO:0019752: carboxylic acid metabolic process
R-HSA-5663202: Diseases of signal transduction by growth factor receptors and se
hsa05014: Amyotrophic lateral sclerosis
R-HSA-199991: Membrane Trafficking
GO:0000278: mitotic cell cycle
GO:0034660: ncRNA metabolic process
GO:0090407: organophosphate biosynthetic process
GO:0034248: regulation of amide metabolic process
GO:0016310: phosphorylation
GO:0006520: amino acid metabolic process
R-HSA-9679506: SARS-CoV Infections
-log10(P)
E
BP10 negatively associated
R-HSA-1640170: Cell Cycle
R-HSA-194315: Signaling by Rho GTPases
R-HSA-8953854: Metabolism of RNA
GO:1903047: mitotic cell cycle process
R-HSA-1280215: Cytokine Signaling in Immune system
WP3888: VEGFA VEGFR2 signaling
GO:1903829: positive regulation of protein localization
GO:0051301: cell division
GO:0006259: DNA metabolic process
R-HSA-3371556: Cellular response to heat stress
WP2882: Nuclear receptors meta pathway
R-HSA-199991: Membrane Trafficking
GO:0005975: carbohydrate metabolic process
GO:0042176: regulation of protein catabolic process
GO:0097435: supramolecular fiber organization
R-HSA-6798695: Neutrophil degranulation
GO:0010564: regulation of cell cycle process
hsa04217: Necroptosis
GO:0098657: import into cell
GO:0051604: protein maturation
-log10(P)
F
Proliferation Associated Proteins Fraction of Total
P=0.0006; Odds Ratio=4.9
P=0.06; O.R.=2.3
P=0.009; O.R.=1.8
P=0.09; O.R.=0.5
P <0.1 for doxycycline induction
AND
P <0.1 for RANBP9 vs RANBP10
237/5347
6/263
27/337
2/19
6/53
3/20
8/43
Observed Proteins
RANBP10list
RANBP9list
RANBP9UbiqUp
RANBP10UbiqDown
RANBP9UbiqDown
RANB10UbiqUp
G
IP
Input (5%)
POLR2B
PABPC1
CACYBP
MCM5
MCM7
IgG
GID8
(kDa)
V5-tag (RanBP9) Short exposure
V5-tag (RanBP9) Long exposure
GID8 Short exposure
GID8 Long exposure
GAPDH
